# Time trends in adolescent depressive symptoms from 2010 to 2019 in Norway: real increase or artifacts of measurements?

**DOI:** 10.1017/S0033291724002447

**Published:** 2024-10

**Authors:** Sondre Aasen Nilsen, Kjell Morten Stormark, Lasse Bang, Geir Scott Brunborg, Marit Larsen, Kyrre Breivik

**Affiliations:** 1 Regional Centre for Child and Youth Mental Health and Child Welfare, NORCE Norwegian Research Centre, Bergen, Norway; 2 Department of Child Health and Development, Norwegian Institute of Public Health, Oslo, Norway; 3 Department of Clinical Neuroscience, Karolinska Institutet, Stockholm, Sweden

**Keywords:** adolescence, depressive symptoms, measurement, psychometrics, time trends

## Abstract

**Background:**

Whether the recent rise in adolescent self-reported depressive symptoms is influenced by changing reporting behavior is much debated. Most studies use observed sum scores to document trends but fail to assess whether their measures are invariant across time, a prerequisite for meaningful inferences about change. We examined whether measurement noninvariance, indicative of changing perceptions and reporting of symptoms, may influence the assessment of time trends in adolescent depressive symptoms.

**Methods:**

Data stem from the nationwide repeated cross-sectional Ungdata-surveys (2010–2019) of 560 712 responses from adolescents aged 13 to 19 years. Depressive symptoms were measured with the Kandel and Davies' six-item Depressive Mood Inventory. Using structural equation modeling, we examined measurement invariance across time, gender and age, and estimated the consequences of noninvariance on cross-cohort time trends.

**Results:**

Across most conditions, the instrument was found measurement invariant across time. The few noninvariant parameters detected had negligible impact on trend estimates. From 2014, latent mean depressive symptom scores increased among girls. For boys, a *U* shaped pattern was detected, whereby an initial decrease in symptoms was followed by an increase from 2016. Larger issues of *noninvariance* were found across age in girls and between genders.

**Conclusions:**

From a measurement perspective, the notion that changed reporting of symptoms has been an important driver of secular trends in depressive symptoms was not supported. Thus, other causes of these trends should be considered. However, noninvariance across age (in girls) and gender highlights that depressive symptoms are not necessarily perceived equivalently from early to late adolescence and across gender.

## Introduction

Studies from Western high-income countries have reported an increase in self-reported depressive and internalizing symptoms among adolescents in the last decade, particularly among adolescent girls (Daly, 2022; Högberg, Strandh, & Hagquist, 2020; Keyes, Gary, O'Malley, Hamilton, & Schulenberg, 2019; Potrebny et al., 2024; Thorisdottir, Asgeirsdottir, Sigurvinsdottir, Allegrante, & Sigfusdottir, 2017; Twenge, Joiner, Rogers, & Martin, 2018). These trends are worrisome, as depression is one of the leading causes of disability-adjusted life years in adolescence (Vos et al., 2020).

Several drivers have been proposed for these trends, including the rise of social media, and increased academic, social, and appearance-related pressures. Studies have also examined changes in socioeconomic, family, and peer relationship variables as putative causes (for a comprehensive review, see Armitage, Collishaw, & Sellers, 2024). However, none of these factors has been found to sufficiently explain the increase in symptom scores.

A distinct alternative hypothesis, and a challenge to time trend research, is that adolescents may have changed how they report symptoms (Armitage et al., 2024). For one, greater knowledge and openness toward mental health may have heightened the ability and willingness to report symptoms (Collishaw, 2015). If so, the trends in self-reported depressive symptoms could reflect a shift toward more accurate symptom reporting. A related view is that common mental states have become more medicalized, lowering the threshold to report normal fluctuations in mood and cognition as pathological (Brinkmann, 2014, 2016). Unifying these perspectives, the *prevalence inflation hypothesis* posits that improved recognition and overinterpretation of symptoms may spiral together, fueling the rise in symptom scores reported in many studies (see Foulkes & Andrews, 2023). Variants of these perspectives are often discussed in trend studies (Collishaw, 2015; Keyes et al., 2019; Pitchforth et al., 2019; Potrebny et al., 2019; van Vuuren, Uitenbroek, Van der Wal, & Chinapaw, 2018). Attention to key principles of psychological measurement provides one pathway for empirically examining such questions. However, few studies have conducted such investigations.

The concept of latent variables plays a pivotal role for the measurement of psychological constructs (e.g. Bollen, 2002; Borsboom, Mellenbergh, & Van Heerden, 2003). By not being directly observable, research has to rely on measuring observable indicators assumed to be manifest expressions of the construct of interest. For depressive symptom measures, each item is usually assumed to be reflective of a unidimensional latent depressive factor (Fried et al., 2016), one of many hypotheses about measurements that may be tested through latent variable modeling, such as factor models within structural equation modeling (SEM; Ziegler & Hagemann, 2015). Thus, latent variable models provide the means to bridge theoretical constructs with empirical data to test hypotheses about the structure and properties of psychological measurements.

To draw valid inferences about differences in scores between groups or across measurement occasions, an essential assumption is measurement invariance (MI; also called *absence of differential item functioning* [*DIF*]). When a measure is invariant, it measures the same construct with the same structure across groups or measurement occasions, demonstrating that the items and the underlying latent construct are interpreted the same way by those groups or over time (see Chen, 2008; Schmitt & Kuljanin, 2008; Van de Schoot, Lugtig, & Hox, 2012; Van De Schoot, Schmidt, De Beuckelaer, Lek, & Zondervan-Zwijnenburg, 2015). For example, a depression measure is invariant across gender if a man and a woman with the same level of depression have the same likelihood of obtaining a given observed score on that scale (Gunn, Grimm, & Edwards, 2020). Conversely, *noninvariance* is indicated when individuals from different groups or measurement occasions with the same standing on the latent construct have *different* likelihoods of obtaining a given score. This *measurement bias* (see Millsap, 2011, p. 47) suggests that items are perceived differently across conditions, which may distort inferences about group differences or temporal changes in the underlying construct (Maassen et al., 2023).

MI provides a framework to examine if secular trends in adolescent depressive symptoms are influenced by an improved accuracy or overreporting of symptoms. Both these hypotheses posit that while adolescents' latent levels have remained stable, their observed scores have risen, falsely suggesting that the prevalence of symptoms has increased. Within the multigroup confirmatory factor analysis approach (MG-CFA), this may be tested by probing the equivalence of item thresholds, factor loadings, and intercepts when the items are ordinal (Wu & Estabrook, 2016). In brief, these steps examine whether each item contributes to the latent factor similarly and whether the level of the latent construct at which a given response option is chosen is the same across groups or measurement occasions. Hence, invariance testing can provide insights into whether differences in depressive symptom scores over time reflect a true difference in the latent construct or from changes in respondents' perception and symptom reporting (Brown, 2015).

Despite the longstanding recognition of MI (Meredith, 1964; Millsap, 2011), there appears to be an underuse of invariance testing within psychological science (see Maassen et al., 2023). To examine current research practices among trend studies into adolescent depressive or internalizing symptoms, we reviewed 20 contemporary studies published since 2017 (see Appendix 1). Of these, 11 discussed the possibility the reported trends could be biased by changing reporting of symptoms, warning about the possible risk of *measurement noninvariance*. Still, only two reported efforts to examine MI across time.

A UK study concluded that changing reporting could not account for the rise in adolescent emotional symptoms, after achieving partial invariance across four birth cohorts (McElroy, Tibber, Fearon, Patalay, & Ploubidis, 2022). However, this study missed out on the opportunity to examine how the detected noninvariant parameters affected the trend estimates and was based on parent informants. A Finnish study found that the item *feeling depressed* was noninvariant across time (1994–2014) in a measure of psychosomatic symptoms among 15-year olds (Hagquist, Välimaa, Simonsen, & Suominen, 2017). Resolving this noninvariant parameter led to stronger trend estimates, highlighting that noninvariance may also deflate trends. In Norway, an older study reported that the Kandel and Davies's Depressive Mood Inventory (as used in the present study) was invariant across three time points (1992, 2002, 2010) (von Soest & Wichstrøm, 2014). However, none of these studies had good coverage of the time period of the increase in depressive symptoms reported from 2010 and onwards.

The repeated cross-cohort design is informative for studying population-level changes over time (Armitage et al., 2024; Collishaw, 2015). However, as also noted by Hagquist et al. (2017), identical instruments and standardized data collections seem to be the major criteria when evaluating their validity, whereas invariance of measures across cohorts is rarely mentioned. Moreover, many studies compare trends across age and gender, which further necessitates MI between age- and gender groups, an assumption that may not hold (e.g. Black, Humphrey, Panayiotou, & Marquez, 2024; Burdzovic Andreas & Brunborg, 2017). Given this poor attention to the fundamentals of measurement, it is worrisome that most previous research relies on observed sum scores to document time-trends. Besides the issue of measurement (non)invariance, sum scores also assume that all items contribute an equal amount of information to the construct being measured. If not justified, the use of sum scores can severely affect the validity and reliability of findings (see McNeish & Wolf, 2020). These oversights are concerning, as trend research may guide policy and health services.

Against this background, this study aimed to examine whether changing reporting of symptoms may influence the assessment of trends in adolescent depressive symptoms. By drawing on first principles of psychological measurement, we investigate the extent to which measurement noninvariance may bias cross-cohort time trends in symptom scores and potentially explain the rise in depressive symptoms among Norwegian adolescents. To achieve this, we used data from the large-scale Ungdata-surveys with more than half-a-million respondents, the most widely referenced source of the rising trends in adolescent depressive symptoms in Norway. In doing so, a broader aim was to raise awareness among researchers about the importance of assessing the factorial invariance of measures used to document trends in adolescent mental health and well-being.

## Methods

### Design and procedure

Data stem from the Ungdata-surveys, a national data collection scheme at the municipal level in Norway. Ungdata comprises a broad set of themes including mental and physical health, living conditions, leisure time activities, and lifestyle behaviors (see http://www.ungdata.no/English). Since 2010, most municipalities have participated. The target population is all junior high (ages 13–15) and high school students (ages 16–18). Data were collected using electronic questionnaires during class time. The annual average response rate amongst participating municipalities ranged from 75–88%.

### Sample

Our sample comprised ten consecutive data collections from 2010 to 2019 (*N* = 628 678, from 414 municipalities). In this sample, 3.7% had missing on age, 3.3% on gender, and missing across the depressive symptom items ranged from 5.4% to 5.9% (4.7% had missing on all items). In total, 13% had missing on one or more of the variables included in this study. For age and gender, missing data were primarily due to privacy concerns, as age was not assessed in small municipalities (< 300 responses). For analysis pooling all age groups (including missing on age), the sample comprised 580 345 responses from 409 municipalities (excluding those with missing on gender and all depressive symptom items). For analyses based on gender by age groups, the sample was restricted to 560 712 responses from 343 municipalities. In both samples, 51% were girls, and the mean age was 15.01 (s.d. = 1.53). Item distributions were highly similar between the two samples.

### Measures

#### Gender and age

Gender was measured by self-report (boys, girls). We used grade as a proxy for age, as age was not explicitly assessed. In Norway, grade attendance is organized by age, with grade 8 corresponding to age 13 and grade 13 corresponding to age 18. In survey years 2010 to 2012, there were few responses from adolescents aged 17 and 18 (*n* = 35–515) compared to other age groups and for these age groups in other survey years (*n* = 763 to 10 278). For our main analyses, we therefore collapsed 17- and 18-year-olds into one age category but kept 17 and 18 as distinct age categories in analyses pooling all survey years together.

*Depressive symptoms* were measured by Kandel and Davies's six-item Depressive Mood Inventory (DMI; Kandel & Davies, 1982), derived from the Hopkins Symptom Checklist (Derogatis, Lipman, Rickels, Uhlenhuth, & Covi, 1974). The DMI measures depressive symptoms during the preceding week with the following items: (1) *Felt that everything is a struggle,* (2) *Had sleep problems,* (3) *Felt unhappy, sad or depressed*, (4) *Felt hopelessness about the future*, (5) *Felt stiff or tense*, and (6) *Worried too much about things*. The items are rated on a four-point scale from (1) ‘Not been affected at all’ to (4) ‘Been affected a great deal’. Items are averaged to produce a mean score ranging from 1 to 4. A previous study based on Ungdata-2017 found support for the unidimensionality of the scale, but noted that items 2 and 6 were noninvariant between boys and girls (Kleppang, Steigen, & Finbråten, 2020). However, multidimensionality of the scale has been suggested in a study from Canada (Brunet et al., 2014).

### Statistical analyses

Measurement invariance (MI) is usually assessed in an item response theory (IRT) framework or a structural equation modeling (SEM) framework. In this study, we focus on the SEM framework using ordinal multigroup confirmatory factor analysis (MG-CFA). The MG-CFA approach is common within most areas of psychology (Putnick & Bornstein, 2016). By properly modeling the threshold structure of ordinal items, the ordinal MG-CFA is similar to the polytomous IRT model (Kim & Yoon, 2011). We start our investigation by examining the dimensionality of the DMI, as ensuring an adequately specified factor structure is a necessary first step and may also provide information about severe violations of measurement invariance across groups and time. We then, as detailed below, focus our investigation on invariance across time by gender and age groups, and invariance across age and gender. Finally, we explicitly model the consequences that changing reporting of symptoms may have for trend estimates.

### Dimensionality assessment

We examined the dimensionality of the DMI by fitting 1-factor CFAs (see below for details of estimation and model fit) and by exploratory factor analyses (EFA) using parallel analysis (O'Connor, 2000). We generated 200 parallel datasets for each parallel analysis using 95% eigenvalue percentiles. We also examined the ratio of first-to-second eigenvalues where a ratio > 4 is supportive of essential unidimensionality (Slocum-Gori & Zumbo, 2011). The CFAs and EFAs were conducted on the pooled sample stratified by gender, by gender stratified by survey year, and by age groups stratified by gender. For the parallel analysis, we also assessed dimensionality for each age group at each survey year by gender, to examine signs of changing dimensionality of the scale across time by age.

### Measurement invariance (MI) analyses

We examined MI using MG-CFA across three dimensions: time (10 survey years), gender (two groups), and age (five groups). Given the complexity of assessing MI across 100 groups (time × gender × age) in one model, we deliberately focused on smaller combinations of groups that considered jointly, offers an overview of most potential issues with MI that may arise by the combination of time, age, and gender.

Our two first set of investigations focused on the question about potential changing perceptions and reporting of symptoms across time:

1. MI was assessed separately for boys and girls across survey years, using survey year as a grouping variable in the MG-CFA models. These analyses provided an initial assessment of whether indications of measurement noninvariance would suggest that either boys or girls aged 13–18 perceive or respond differently to the items across time.
2. Subsequently, we assessed MI for each age group across survey years, separately for boys and girls. These analyses examined potential changes in responding over time separately for different age groups. In other words, whether younger or older adolescents' girls or boys have changed their perceptions of the items over time.

We then proceeded to examine MI across age and gender groups (steps 3–4). The aim of these analyses were to investigate potential gender or age differences in how the items were perceived, and whether comparing time trends across age or gender groups could be biased by measurement noninvariance:

1. MI between age groups by gender on the pooled sample (across all survey years) and at each survey year was examined. In order to compare trends between age groups, the measure should be invariant between all age groups at each survey year.
2. We examined MI between boys and girls on the pooled sample and for each survey year, to assess whether comparing latent means between boys and girls at a given year and across time could be influenced by gender noninvariance.

### Trends and practical significance of violation of invariance

If achieving full or partial scalar invariance, trends in latent means of the DMI were assessed by gender and age. The trends were centered using 2014 as the reference year. To examine the practical significance of any noninvariance detected on trend estimates, we compared differences in latent mean trends when accounting for (i.e. freeing) and not accounting for (i.e. fixing) noninvariant parameters (Oberski, Vermunt, & Moors, 2015). To quantify the magnitude of noninvariance, we also calculated the *differences in mean and covariance structures* (*d*_MACS_) effect size (Nye & Drasgow, 2011b). As tentative guidelines, *d*_MACS_ between 0.20–0.40 may be considered small, 0.40–0.70 as medium, and 0.70 or greater as large effect sizes (Nye, Bradburn, Olenick, Bialko, & Drasgow, 2019).

### Model estimation, procedure of invariance testing, and evaluating model fit

We used a mean- and variance-adjusted weighted least squares (WLSMV) estimation suitable for ordinal items (Flora & Curran, 2004). We did *not* rely on the traditional fit index cutoffs to assess model fit (i.e. comparative fit index [CFI] > 0.95 and root mean square error of approximation [RMSEA] < 0.06; Hu & Bentler, 1999 [HB]) as these were derived from simulations of a three-factor model with 15 continuously measured items using maximum likelihood estimation (McNeish, 2023; Nye & Drasgow, 2011a). Instead, we used dynamic fit indices (DFI) for ordinal one-factor models (McNeish, 2023; McNeish & Wolf, 2023). The DFI-method is a simulation-based approach that answers what HBs' recommendations would have been, had they simulated a model corresponding to the characteristics of the data and model tested (sample size, factor structure, number of items etc.). The DFI yields cut- offs corresponding to three levels of model fit: Level 0 (perfect fit; no model misspecification), Level 1 (close fit; misspecification equal in magnitude to that of HBs' cut offs) and Level 2 (misspecification twice in magnitude to that of HB). We chose Level 1 as a cut-off for all models. Missing data was excluded by pairwise deletion.

Evaluation of MI with ordinal indicators is less straightforward than with continuous indicators, due to the indeterminacy in the location and scale of the latent response variable. Our strategy is therefore based on recent advances in invariance testing with ordinal items, as suggested by Wu and Estabrook (2016). That is, using delta parameterization, we sequentially probed invariance with respect to (1); thresholds; (2) factor loadings; and (3) latent response intercepts (scalar invariance). Only when scalar invariance is met are comparisons of differences in the latent means possible. If either step failed, we planned to identify the location of the measurement issues and examine whether partial invariance was feasible. We accepted partial invariance if no more than half (3/6) of the parameters were needed to be released (Steenkamp & Baumgartner, 1998).

To determine the equivalence of nested models, we drew on simulations examining the performance of delta (Δ) fit indices under similar conditions as the present study; six item unidimensional scale with ordinal items across many groups (10–20) with large group sample sizes (*n* = 600–6000 per group), suggesting a cutoff of ΔRMSEA ⩽ 0.01 in conjunction with a ΔCFI ⩾ −0.004 for equal threshold and loading invariance (Rutkowski & Svetina, 2017). As that study did not examine equal intercept invariance (in par with Wu & Estabrook, 2016), and as we were more worried of missing consequential noninvariant parameters than detecting inconsequential ones, we chose the stricter ΔCFI ⩾ −0.002 as the main cut-off, as proposed by Meade, Johnson, and Braddy (2008). Given the large sample size, we did not rely on the Δχ^2^ (Bentler & Bonett, 1980).

### Sensitivity and robustness analyses

In Ungdata 2010, the response options of the DMI were reversed compared to the other survey years, potentially affecting the properties of the instrument that year. The main invariance analyses across time by gender and age groups were therefore re-examined by excluding year 2010. We also conducted MI analyses comparing one earlier survey year to the latest survey year, as signs of noninvariance might be hidden when comparing many groups. We chose 2013 as the early survey year, as the sample size and participation rate from senior high school students were lower for survey years 2010–2012, and selected three age groups (13, 15, and 17–18 year olds).

Ungdata has a three-level nesting structure, with individual responses (level 1) nested in municipality- years (level 2) nested in municipalities (level 3). Using observed item scores as outcome measures, intercept only multilevel models were conducted to gauge how strong the clustering effects of the DMI-items were at these higher units, using data from all survey years. The intraclass correlation coefficients (ICCs) for each item were very low for girls (municipality-years ICC range: 0.009–0.031, municipality ICC range: 0.003–0.008) and boys (municipality-years ICC range: 0.008–0.017, municipality ICC range: 0.001–0.005). Given the weak clustering effects at these higher units and the challenge of addressing this clustering within the MG-CFA framework when using WLSMV estimation for ordinal data, we chose to not pursue this any further. The low ICCs suggest that clustering effects would likely have a small impact on the precision of the standard errors.

Pairwise deletion was used to retain as much information possible in the analyses, as using multiple imputation for all analyses became too computationally demanding given the sample size, ordinal nature of the data, and the sheer number of models tested. However, we checked the robustness of our main models assessing measurement invariance across survey years by gender and age groups, using multiple imputation. A description of our imputation strategy is given in the online Supplementary Materials (p. 44).

Analyses were performed using R version 4.2.2 for Mac. Data preparations, visualizations, and formal analyses were conducted using the R-packages *tidyverse* (Wickham et al., 2019), *lavaan* (Rosseel, 2012), *semTools* (Jorgensen, Pornprasertmanit, Schoemann, & Rosseel, 2020), *psych* (Revelle, 2022), dynamic (Wolf & McNeish, 2023), and *dmacs* (Dueber, 2023), lme4 (Bates, Mächler, Bolker, & Walker, 2015), and mice (van Buuren & Groothuis-Oudshoorn, 2011). A vignette with R-code demonstrating the main analyses is available on the Open Science Framework (OSF; https://osf.io/pxabg/). Significance level (*α*) was set at *α* = 0.05.

## Results

Descriptive characteristics of the depressive symptom items by gender and survey year, *excluding* respondents with missing information about age, are shown in Table 1 (see online Supplementary Table [ST] S1 for the equivalent table *including* those with missing on age). Descriptively, depressive symptoms increased over time for girls, and to a lesser extent among boys.

**Table 1.** Descriptive characteristics of the depressive symptom items by gender and survey year

Girls	2010, *N* = 7295	2011, *N* = 3202	2012, *N* = 11 552	2013, *N* = 39 449	2014, *N* = 21 278	2015, *N* = 33 619	2016, *N* = 32 105	2017, *N* = 47 513	2018, *N* = 33 643	2019, *N* = 55 527
Age	14.40 (1.22)	14.46 (1.23)	14.74 (1.19)	14.70 (1.40)	14.89 (1.55)	15.10 (1.60)	14.93 (1.44)	15.13 (1.60)	15.24 (1.66)	15.30 (1.62)
1. Struggle	2.40 (0.99)	2.25 (0.95)	2.40 (0.98)	2.38 (0.98)	2.40 (1.00)	2.47 (1.02)	2.47 (1.02)	2.53 (1.01)	2.54 (1.02)	2.57 (1.01)
Skew/kurtosis^a^	0.20/−1.00	0.36/−0.77	0.16/−0.99	0.19/−0.97	0.16/−1.03	0.08/−1.11	0.08/−1.11	0.02/−1.09	0.01/−1.12	−0.03/−1.09
(% missing)	(0.5%)	(0.4%)	(0.5%)	(0.6%)	(0.6%)	(0.6%)	(0.6%)	(0.7%)	(0.9%)	(0.9%)
2. Sleep problems	2.08 (0.98)	2.01 (0.96)	2.16 (0.99)	2.11 (0.97)	2.11 (0.98)	2.13 (0.99)	2.13 (0.99)	2.19 (1.00)	2.22 (1.01)	2.27 (1.02)
Skew/kurtosis	0.55/−0.71	0.63/−0.58	0.44/−0.86	0.50/−0.74	0.50/−0.77	0.49/−0.82	0.49/−0.81	0.43/−0.89	0.38/−0.96	0.34/−0.99
(% missing)	(0.3%)	(0.4%)	(0.4%)	(0.4%)	(0.4%)	(0.4%)	(0.3%)	(0.5%)	(0.6%)	(0.5%)
3. Depressed	2.03 (0.98)	2.03 (0.97)	2.12 (0.99)	2.13 (1.01)	2.08 (1.02)	2.10 (1.02)	2.11 (1.03)	2.19 (1.03)	2.21 (1.03)	2.28 (1.03)
Skew/kurtosis	0.62/−0.65	0.61/−0.64	0.49/−0.82	0.49/−0.86	0.55/−0.84	0.52/−0.87	0.51/−0.91	0.40/−1.00	0.38/−1.02	0.30/−1.05
(% missing)	(0.6%)	(0.4%)	(0.5%)	(0.6%)	(0.5%)	(0.5%)	(0.6%)	(0.6%)	(0.8%)	(0.7%)
4. Hopeless	1.89 (0.99)	1.83 (0.97)	1.96 (1.01)	1.97 (1.02)	1.99 (1.04)	2.05 (1.05)	2.03 (1.06)	2.10 (1.06)	2.15 (1.07)	2.18 (1.07)
Skew/kurtosis	0.82/−0.46	0.91/−0.27	0.71/−0.65	0.70/−0.71	0.66/−0.82	0.59/−0.92	0.62/−0.90	0.51/−1.02	0.45/−1.09	0.41/−1.11
(% missing)	(0.6%)	(0.7%)	(0.7%)	(0.7%)	(0.7%)	(0.7%)	(0.7%)	(0.8%)	(0.9%)	(0.9%)
5. Tense	2.01 (0.98)	1.98 (0.95)	2.04 (0.98)	2.06 (0.98)	1.98 (0.97)	2.04 (0.99)	2.03 (1.00)	2.17 (1.01)	2.19 (1.03)	2.21 (1.03)
Skew/kurtosis	0.63/−0.65	0.64/−0.57	0.56/−0.75	0.54/−0.76	0.64/−0.64	0.57/−0.77	0.58/−0.79	0.39/−0.98	0.38/−1.03	0.35/−1.04
(% missing)	(0.9%)	(0.8%)	(1.1%)	(0.9%)	(1.0%)	(1.0%)	(1.0%)	(1.1%)	(1.4%)	(1.5%)
6. Worried	2.41 (1.02)	2.30 (0.99)	2.43 (1.01)	2.46 (1.02)	2.49 (1.03)	2.55 (1.04)	2.57 (1.05)	2.71 (1.02)	2.74 (1.03)	2.77 (1.02)
Skew/kurtosis	0.13/−1.11	0.24/−0.99	0.08/−1.09	0.06/−1.11	0.02/−1.14	−0.05/−1.17	−0.08/−1.19	−0.23/−1.09	−0.27/−1.08	−0.30/−1.05
(% missing)	(0.6%)	(0.8%)	(0.7%)	(0.7%)	(0.6%)	(0.7%)	(0.7%)	(0.8%)	(1.0%)	(0.8%)
Mean score	2.14 (0.77)	2.07 (0.76)	2.19 (0.78)	2.18 (0.79)	2.18 (0.79)	2.22 (0.81)	2.23 (0.82)	2.31 (0.81)	2.34 (0.82)	2.38 (0.81)
Skew/kurtosis	0.52/−0.47	0.64/−0.26	0.45/−0.54	0.47/−0.58	0.46/−0.62	0.40/−0.72	0.40/−0.75	0.29/−0.81	0.25/−0.83	0.21/−0.85
(% missing)	(2.2%)	(2.2%)	(2.4%)	(2.5%)	(2.4%)	(2.4%)	(2.5%)	(2.8%)	(3.2%)	(3.1%)

Boys	2010, *N* = 6872	2011, *N* = 3259	2012, *N* = 11 013	2013, *N* = 38 187	2014, *N* = 20 975	2015, *N* = 31 884	2016, *N* = 31 332	2017, *N* = 46 803	2018, *N* = 31 714	2019, *N* = 53 490
Age	14.33 (1.15)	14.51 (1.26)	14.74 (1.16)	14.68 (1.36)	14.85 (1.51)	15.07 (1.54)	14.90 (1.41)	15.05 (1.55)	15.17 (1.62)	15.26 (1.59)
1. Struggle	2.08 (0.95)	1.87 (0.88)	2.00 (0.94)	1.91 (0.90)	1.88 (0.90)	1.91 (0.92)	1.88 (0.91)	1.93 (0.93)	2.00 (0.96)	1.99 (0.95)
Skew/kurtosis^a^	0.57/−0.59	0.83/−0.03	0.65/−0.48	0.74/−0.24	0.78/−0.21	0.75/−0.34	0.79/−0.25	0.73/−0.38	0.65/−0.57	0.66/−0.53
(% missing)	(0.5%)	(0.5%)	(0.7%)	(0.7%)	(0.6%)	(0.6%)	(0.6%)	(0.7%)	(1.0%)	(0.9%)
2. Sleep problems	1.89 (0.94)	1.76 (0.87)	1.90 (0.94)	1.82 (0.90)	1.80 (0.89)	1.83 (0.91)	1.81 (0.90)	1.88 (0.92)	1.93 (0.95)	1.95 (0.96)
Skew/kurtosis	0.82/−0.29	0.97/ 0.15	0.78/−0.34	0.87/−0.08	0.90/−0.05	0.86/−0.15	0.91/−0.04	0.80/−0.27	0.74/−0.43	0.71/−0.49
(% missing)	(0.4%)	(0.4%)	(0.6%)	(0.6%)	(0.5%)	(0.6%)	(0.4%)	(0.7%)	(0.9%)	(0.6%)
3. Depressed	1.63 (0.84)	1.55 (0.80)	1.61 (0.82)	1.59 (0.81)	1.56 (0.81)	1.59 (0.83)	1.57 (0.82)	1.68 (0.87)	1.73 (0.91)	1.77 (0.92)
Skew/kurtosis	1.29/ 0.97	1.43/ 1.41	1.27/ 0.93	1.33/ 1.09	1.41/ 1.28	1.35/ 1.05	1.37/ 1.17	1.16/ 0.47	1.08/ 0.21	1.00/ 0.05
(% missing)	(0.6%)	(0.8%)	(0.8%)	(0.8%)	(0.6%)	(0.7%)	(0.7%)	(0.9%)	(1.1%)	(0.9%)
4. Hopeless	1.62 (0.87)	1.54 (0.82)	1.61 (0.86)	1.57 (0.84)	1.57 (0.85)	1.60 (0.87)	1.57 (0.85)	1.67 (0.91)	1.72 (0.94)	1.74 (0.95)
Skew/kurtosis	1.30/0.79	1.47/1.35	1.32/0.88	1.42/1.17	1.42/1.11	1.35/0.88	1.42/1.11	1.21/0.44	1.10/0.12	1.07/0.03
(% missing)	(0.6%)	(0.8%)	(0.9%)	(0.9%)	(0.7%)	(0.8%)	(0.8%)	(1.0%)	(1.2%)	(1.2%)
5. Tense	1.77 (0.89)	1.65 (0.82)	1.71 (0.85)	1.68 (0.84)	1.61 (0.80)	1.62 (0.81)	1.57 (0.78)	1.67 (0.83)	1.68 (0.86)	1.69 (0.86)
Skew/kurtosis	0.98/0.12	1.17/0.75	1.04/0.31	1.09/0.47	1.21/0.84	1.20/0.73	1.28/0.98	1.10/0.44	1.12/0.41	1.09/.36
(% missing)	(0.8%)	(0.9%)	(1.1%)	(1.1%)	(1.0%)	(1.1%)	(1.1%)	(1.3%)	(1.7%)	(1.7%)
6. Worried	1.91 (0.96)	1.75 (0.90)	1.87 (0.94)	1.81 (0.91)	1.81 (0.92)	1.86 (0.95)	1.83 (0.94)	1.97 (0.98)	2.01 (1.00)	2.04 (1.01)
Skew/kurtosis	0.77/−0.44	0.98/0.01	0.78/−0.43	0.88/−0.18	0.88/−0.21	0.81/−0.39	0.86/−0.30	0.65/−0.68	0.60/−0.80	0.57/−0.83
(% missing)	(0.8%)	(0.8%)	(1.0%)	(1.0%)	(0.8%)	(1.0%)	(0.9%)	(1.0%)	(1.3%)	(1.1%)
Mean score	1.81 (0.70)	1.68 (0.65)	1.78 (0.68)	1.73 (0.66)	1.70 (0.65)	1.73 (0.68)	1.70 (0.66)	1.80 (0.70)	1.84 (0.72)	1.86 (0.73)
Skew/kurtosis	0.97/0.57	1.15/1.04	0.99/0.65	1.08/0.89	1.13/1.00	1.08/0.77	1.12/0.89	0.97/0.42	0.91/0.24	0.88/0.15
(% missing)	(2.2%)	(2.9%)	(2.9%)	(2.6%)	(2.5%)	(2.7%)	(2.4%)	(2.9%)	(3.5%)	(3.3%)

a Excess kurtosis shown for all items.

All results presented as mean (s.d.) unless otherwise stated. Items scored on a scale ranging from 1 (‘not at all affected’) to 4 (“extremely affected).

### Unidimensionality of the depressive symptom scale

The DMI was found to be sufficiently unidimensional in all groups, after allowing one pair of correlated residuals. Model fit estimates and dynamic fit indices (DFIs) from the 1-factor CFAs by gender, gender by survey year, and age groups by gender are shown in online ST S2, S3. Pooling all survey years, fit measures were similar between girls (CFI = 0.995, RMSEA [90% CI] 0.067 [0.066–0.068]) and boys (CFI = 0.994, RMSEA [90% CI] 0.064 [0.063–0.065]). Factor loadings (*λ*) were strong and similar for the items, with a mean [M] of 0.79 (standard deviation [s.d.] = 0.07, range: 0.67–0.85) for girls, and *λ*_M_ = 0.78 (s.d. = 0.08, range: 0.65–0.86) for boys. The internal consistency of the scale was high (Girls: omega [*ω*] = 0.89, Boys: *ω* *=* 0.87). By survey years and age groups, factor loadings, internal consistency and fit estimates were similar and fluctuated around the overall estimates reported above.

The DFI-cutoffs (Level 1) across models ranged from CFI: 0.994 to 0.996, RMSEA: 0.053 to 0.067, and SRMR: 0.021 to 0.026. Most models slightly exceeded one or more cutoffs. For most models, improvement in model fit could be obtained by allowing correlated error terms for item 3 (unhappy, sad or depressed) and item 4 (hopelessness). For a few models, however, most improvement could be obtained by allowing correlated residuals between item 2 (sleep problems) and 5 (stiff/tense) (boys years 2010-2011) or item 3 and 5 (girls 2010-2014). Allowing these modifications led to acceptable fit in all but two models (boys 2010, 2012), where RMSEA values remained slightly too high.

Results from the parallel analyses were consistent with the support of the one factor solution in the CFA (online Supplementary Figure [SF] S1–S4). Although two and three factors were suggested for certain age/gender/time combinations, one dominant factor emerged for all models, and the parallel plots were highly homogenous with no indication of changing dimensionality over time. Any additional factors explained very little variance and yielded no clear interpretational meaning. The ratio of first-to-second eigenvalue was > 4 across all EFAs, yielding further support to unidimensionality.

### Measurement invariance across survey years by gender

Pooling all age groups, the DMI was judged to be measurement invariant among boys and girls across survey years. That is, the fit of the baseline MG-CFA models was within the limits set by the DFI's after allowing the correlated error term between item 3 and 4. Sequentially fixing thresholds, loadings, and intercepts did not lead to increased model misfit (**Δ**CFI < 0.002; Table 2). Factor loadings of all items were also highly similar across survey years (online SF S5). Hence, our first line of inquiry did *not* find evidence of changed reporting of symptoms across time.

**Table 2.** Model fit and measurement invariance by survey years for boys and girls

Model	χ^2^ (*df*)	*p*	CFI	RMSEA (90% CI)	TLI	SRMR	ΔCFI	ΔRMSEA
Girls								
Baseline model	12 288.161 (90)	<0.001	0.995	0.068 (0.067–0.069)	0.991	0.021		
Baseline model^a^	6782.332(80)	<0.001	0.997	0.053 (0.052–0.054)	0.994	0.016		
Thresholds	7168.87 (134)	<0.001	0.997	0.042 (0.041–0.043)	0.997	0.016	−0.0001	−0.0111
Thresholds, loadings	5934.849 (179)	<0.001	0.997	0.033 (0.032–0.034)	0.998	0.016	0.0006	−0.0092
Thresholds, loadings, intercepts	9395.612 (224)	<0.001	0.996	0.037 (0.037–0.038)	0.997	0.016	−0.0015	0.0042
Boys								
Baseline model	10 705.745 (90)	<0.001	0.994	0.064 (0.063–0.065)	0.989	0.023		
Baseline model^a^	6400.831 (80)	<0.001	0.996	0.053 (0.052–0.054)	0.993	0.018		
Thresholds	6538.528 (134)	<0.001	0.996	0.041 (0.040–0.042)	0.996	0.018	−0.0001	−0.0117
Thresholds, loadings	5399.731 (179)	<0.001	0.997	0.032 (0.031–0.033)	0.997	0.018	0.0007	−0.0090
Thresholds, loadings, intercepts	8259.24 (224)	<0.001	0.995	0.035 (0.035–0.036)	0.997	0.019	−0.0017	0.0035

a Modeling correlated error terms between items 3 and 4.

*Note*. χ^2^, chi-square goodness-of-fit based on the Satorra–Bentler correction; df, degrees of freedom; CFI, comparative fit index; RMSEA, root mean square error of approximation; CI, confidence interval; TLI, Tucker Lewis Index; SRMR, standardized root mean squared residual; **Δ** CFI/RMSEA, change in CFI/RMSEA between models.

### Measurement invariance by age groups across survey years, by gender

MI was also evident for most age groups across survey years (online STS4, S5). For girls, scalar invariance was detected for all ages except for 15-year-olds, where freeing intercepts of item 6 (*worried*) was needed to achieve partial scalar invariance. For boys, scalar invariance was achieved for 13- and 17–18-year-olds. For those aged 14 to 16, partial scalar invariance was achieved after freeing intercepts of item 5 (*stiff/tense*). Having achieved partial- or full scalar invariance, we proceeded to examining trends in latent means.

### Time trends in depressive symptoms by age and gender

**Figure 1.**
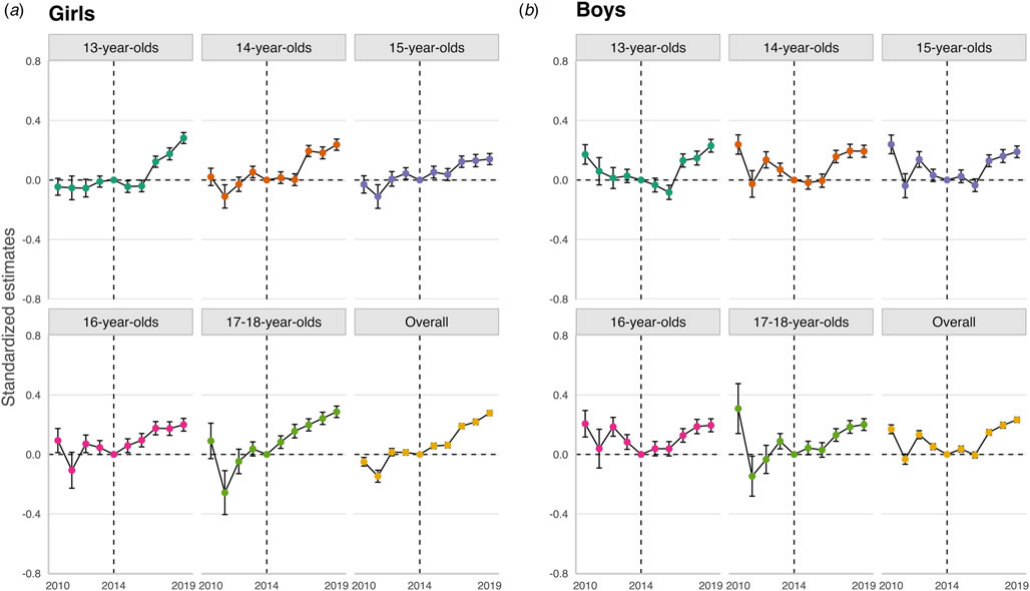
Time trends in latent mean depressive symptom scores among girls (a) and boys (b) by age groups. *Note*. This figure shows trends in standardized latent mean scores of the depression inventory by gender and age groups from the age stratified multigroup confirmatory factor analyses. For girls aged 15 and boys aged 14–16, the latent means were derived from the partial scalar invariance models, freeing intercepts of item 5 (Boys) and item 6 (girls).The trends are centered using the year 2014 as reference (the dotted horizontal line). Thus, point estimates and associated error bars (95% confidence intervals) reflect the yearly deviation in latent means compared to 2014 expressed in standardized deviation units. Point estimates with error bars not crossing the dotted horizontal line are statistically significantly different from zero at *p* < 0.05.

Depressive symptoms generally increased over time, but the pattern varied according to gender and age. For girls, latent means were fairly stable from 2010 to 2014 in all age groups. For those aged 13 to 15, latent means increased from 2016 and onwards, whereas for 16–18-year-olds, the increase started from 2014. Compared to the reference year 2014, latent means were about 0.14 to 0.29 standard deviation units higher in 2019, depending on age. For boys, a u-shaped pattern emerged. Although latent means were significantly higher in 2019 compared to 2014 (with about 0.19 to 0.23 standard deviations), latent means were at about the same level in 2019 as in 2010 (see Fig. 1; online ST S6–S7 for details).

### The magnitude of noninvariance and consequences on time trends in depressive symptoms

For 15-year-old girls, the freely estimated intercept means of item 6 (worried) slightly *increased* from 2010 to 2019, indicating that girls became more likely to endorse this item over time given the same trait value on depressive symptoms. The *d*_MACS_ effect sizes of this noninvariant parameter were small across survey years (range 0.05 to 0.13), using 2010 as reference. For 14- to 16-year-old boys, intercept means of item 5 (Tense) slightly increased from 2010 to 2014, before decreasing to 2019. The associated *d*_MACS_ were also in boys small across all age groups (all ⩽0.2).

**Figure 2.**
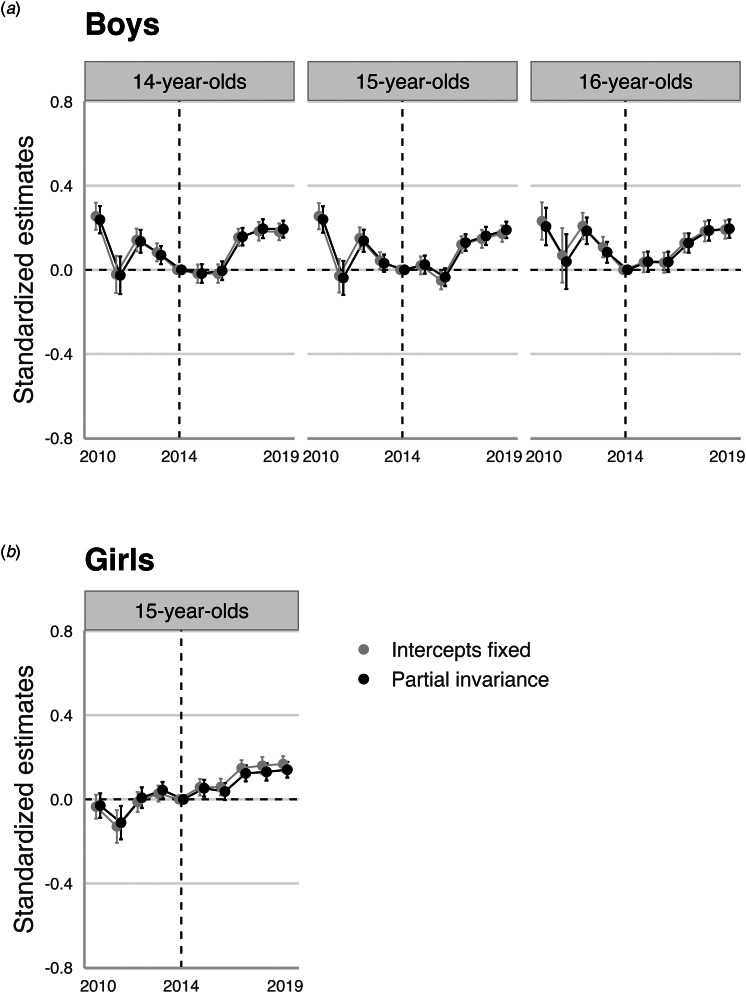
The impact of noninvariant intercepts for boys (a) and girls (b) on trend estimates. *Note.* This figure shows latent trend estimates comparing models accounting (i.e. partial scalar invariance, in black) and not accounting (i.e. intercepts fixed, in grey) for noninvariant intercepts of item 5 (tense) among 14–16-year old boys, and noninvariant intercepts of item 6 (worried) in 15-year-old girls. The trends are centered using the year 2014 as reference (the dotted horizontal line). Thus, point estimates and associated error bars (95% confidence intervals) reflect the yearly deviation in latent means compared to 2014 expressed in standardized deviation units. Point estimates with error bars not crossing the dotted horizontal line are statistically significantly different from zero at *p* < 0.05.

The practical consequences of these noninvariant intercepts on trend estimates are displayed in Fig. 2. As shown, not accounting for the noninvariant parameters yielded highly similar trends as when accounted for (i.e. *partial invariance model*), and latent means only differed by about 0.01 to 0.03 standard deviation units across survey years (see online ST S8–S15).

### Measurement invariance between age groups across survey years

For boys, scalar invariance was detected overall and at each survey year, indicating that comparing trends in latent means across age in boys were warranted. For girls, issues with *noninvariance* were found in all models. In most cases, partial scalar invariance was achieved after freeing intercepts of item 2 (sleeping problems) and item 3 (unhappy/depressed) or freeing only intercepts of item 3 (3/10 survey years; online ST S16–S18). The intercept means of these two items were lower for older adolescent. The *d*_MACS_ suggested a small to medium effect sizes of the noninvariance, with the largest effect sizes found between 13- and 18-year-olds.

**Figure 3.**
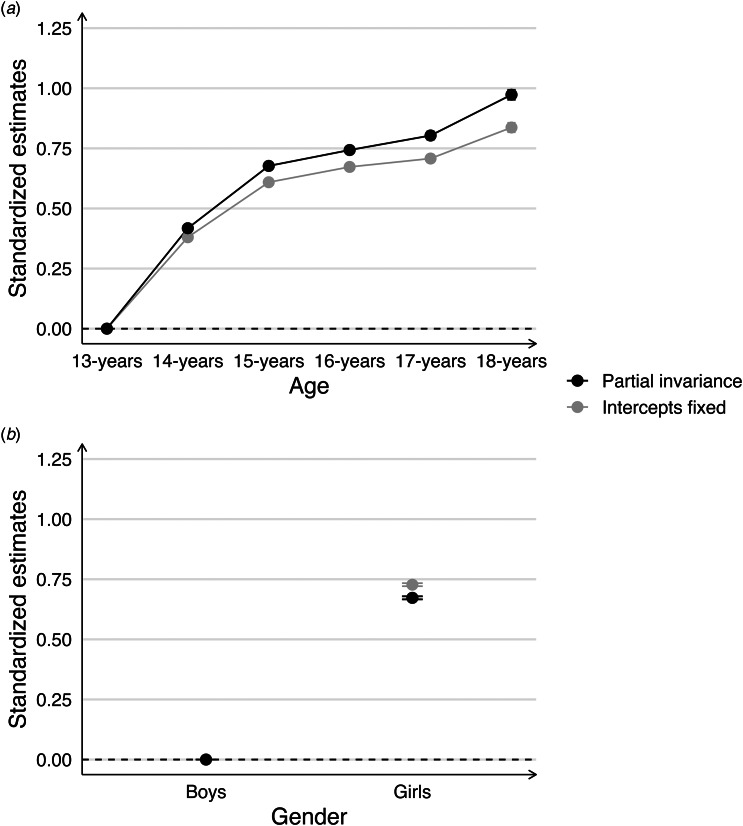
The impact of noninvariance on latent means across age in girls (a) and gender (b) on the pooled sample. *Note*. Panel **A** shows latent means in depression score by age groups for girls comparing partial scalar invariance model (freeing intercepts of item 2 and 3) and a fixed model where all item intercepts are forced to be equal. The reference group for both models were 13-year olds. Panel **B** shows latent means in depression scores by gender (reference Boys) comparing partial scalar invariance model (freeing intercepts of item 6) and a fixed model where all item intercepts are forced to be equal. Each point with associated 95% confidence interval thus represent the difference in latent mean scores expressed in standardized units between each age group and the reference group in the two models.

The practical consequences of these noninvariant parameters on the pooled sample are shown in Fig. 3. Not accounting for this noninvariance deflated differences in latent means, particularly between the youngest and the oldest adolescents (~0.14 standard deviation units). However, the magnitude of this noninvariance was fairly stable at each survey year, suggesting that differences in how these items were perceived between age groups among girls had not changed much across time (online ST S19–S31; SF S6).

### Measurement invariance by gender and survey years

Finally, we assessed MI by gender and by gender and time, to gauge whether measurement noninvariance between boys and girls could influence comparisons of trends between the genders. In brief, freeing intercepts of item 6 (*worries*) and for selected years item 2 (*sleep problems*) were needed to achieve *partial* scalar invariance. Girls had a higher intercept means on item 6 but lower intercept means on item 2. Not accommodating these invariant parameters slightly inflated differences in latent means by about 0.05 standard deviation units overall (Fig. 3a, online ST S32–S34). Similar estimates were obtained at each survey year with no systematic changes across time (online ST S35–S37, SF S7).

### Sensitivity and robustness analyses

Excluding year 2010 from the analyses yielded the same conclusion for the main MI analyses across survey years by age and gender (online ST S38, S39). By removing year 2010, the trend became somewhat more linear for older adolescent girls and less curvilinear for boys (equivalent to the trend displayed in Fig. 1 excluding year 2010). The same general conclusion also held when comparing 2013 to 2019. For girls, invariance was achieved for all age groups. For boys, item 5 (tense/stiff) was flagged as noninvariant among 13- and 15-year olds, but had negligible impact on differences in latent means between the two years (online ST S40–S43).

The results of our main models (MI across survey years by gender and age groups) were highly similar when based on multiple imputed data, both in terms of fit indices and trends in latent mean scores across survey years (see online ST S44–S49 and SF S8, S9). Thus, we found no indications that missing data and the use of pairwise deletion posed a threat to the validity of the main findings reported.

## Discussion

Inspired by the debate of whether the recent rise in adolescent depressive symptoms may be influenced by changing reporting of symptoms, we assessed whether measurement noninvariance may influence the assessment of time trends in adolescent depressive symptoms from 2010 to 2019. An increase in depressive symptoms was evident among girls and boys from 2014/2016 and onwards. However, we found no systematic evidence of measurement noninvariance as a viable explanation for this increase in symptom scores, suggesting that the cross-cohort time trends represent a real change at the latent level. In contrast, larger issues of noninvariance by age and gender were detected, rendering comparisons of depressive symptoms across these groups more problematic.

For girls, the only evidence of noninvariance across time was detected for item 6 (*worried*), where 15-year-olds became *more* likely to report this symptom, at the same level of the latent construct. For boys, intercept means for item 5 (*stiff/tense*) decreased for 14- to 16-year-olds, indicating that they became *less* likely to report this symptom, given the same level on the latent construct. However, the magnitude of noninvariance of these two parameters was small and their practical consequences for trend estimates were trivial. Thus, although our findings indicated a slight change in meaning of these two items for these specific gender and age groups, we detected no clear evidence of changing reporting behavior as a viable explanation of the increase in latent scores from 2010 to 2019.

Very few trend studies have assessed the equivalence of their instrument across time. The study by McElroy et al. (2022) was one exception, which akin to ours, reported that *noninvariance* could not account for the rising trend in emotional symptoms. That study did, however, not examine the impact of the noninvariant parameters detected on trend estimates and was based on parent reports. Another study found that a measure of psychosomatic symptoms was noninvariant across time, mostly due to changing meaning of the item *feeling depressed* in girls (Hagquist et al., 2017). Our results also mostly align with an earlier Norwegian study, which reported the DMI to be invariant across three time points from 1992 to 2010 (von Soest & Wichstrøm, 2014). Differences in time frames and methodology impede a more fine-tuned comparison. However, all studies found an increase in symptoms among girls, even after accounting for issues of noninvariance. Notwithstanding, they also illustrate that equivalence of measures across time should not be assumed but examined using psychometric methods.

Larger measurement issues were detected across age in girls and between genders, where two items showed signs of noninvariance. If not accounted for, differences in depressive symptoms between younger and older adolescent girls were *underestimated*, and differences between genders slightly *overestimated*. The magnitude of noninvariance was, however, fairly stable across survey years, suggesting that differences in how these items were perceived by age (in girls) and gender, had not systematically changed. Thus, these noninvariant parameters mostly affected comparisons of overall levels of depressive symptoms rather than slopes of the time trend between these groups.

None of the reviewed trend studies examined invariance across age and gender, although trend comparisons by these groups were pivotal in most. However, based on the existing psychometric literature, noninvariance by age and gender should come as no surprise. For example, a recent large-scale study of five mental health and well-being measures in adolescence found that all showed noninvariant intercepts between age and gender groups (Black et al., 2024). Our results echo these findings and the conclusion that measurement invariance should be examined when the interest lies in comparing mental health between age and gender groups.

### Strengths and limitations

A strength of this study was the nationwide annually collected data with a high response rate. The data spanned from 2010 to 2019, making it well suited to investigate changing reporting behavior as an explanation of secular trends in adolescents self-reported depressive symptoms. Related, the large sample size provided a unique opportunity to examine this question across age and gender. Another significant strength was the thorough psychometric investigation using state-of-the-art methods, including dynamic rather than static fit indices cut-offs (McNeish, 2023), basing the MI-analyses on recent advances in ordinal invariance testing (Wu & Estabrook, 2016), and by explicitly assessing the consequences that noninvariance may have for group comparisons and trend estimates.

However, there are also some limitations. First, though we believe our results generalize well to the Norwegian adolescent population, the extent to which these results can be transferred to other measures of depressive symptoms and populations is unclear. That is, other instruments may have different properties and measurement issues. Likewise, differences in language and perceptions of symptoms means that one cannot automatically generalize these results to other countries – even if the same instrument is used. We therefore hope that future studies can perform similar investigations on other measures and in other contexts.

As psychometrics is a continuously evolving field, there are also some uncertainties and unresolved issues the reader should be aware of. For one, the performance of absolute and delta fit indices in assessing model fit are debated. We therefore chose rather strict criteria for inferring invariance, which seemed to work well, as we identified issues of noninvariance from small and inconsequential, to large and practically meaningful. Another issue is that under certain conditions, MI-testing may not work optimally. Notably, if the threshold for symptom reporting had changed equally across time for all items, noninvariance may go undetected (Little, 2000). Thus, we cannot exclude the possibility that some changes in perception and reporting of symptoms have occurred in ways we are unable to detect by altered psychometric properties of the instrument.

Despite the large sample, the number of participating municipalities and individual responses were lower for the earlier years of Ungdata (2010–2012), particularly for senior high school students. This impacts the precision of the estimates and may limit the generalizability of the trend estimates for these years. Moreover, due to the challenge of addressing for clustering effects within the MG-CFA framework with ordinal items, we did not correct for clustering of responses at the municipality-year and municipality level. Although this may have improved the precision of the standard errors of our analyses, we consider this to likely have minimal impact on our main results, as the clustering effects were very small for boys and girls.

Another potential limitation is that of missing data. Although we confirmed the robustness of our main results using multiple imputation, such a strategy still relies on the assumption that data is missing at random (MAR). If this assumption does not hold, for instance, if there is some unmodeled selection into missing data based on unobserved factors, there may be some bias in the estimates reported.

A final limitation, which this study shares with most cross-cohort time trend studies (Collishaw, 2015), is that we cannot distinguish between age, period, and cohort effects in explaining the rise in symptom scores observed. Studies of longitudinal birth cohorts assessed at different points in time are needed to better examine such questions.

## Conclusions

Similar to findings from many other Western countries, depressive symptom scores have increased among Norwegian adolescent girls from 2010 to 2019. We found no systematic evidence of measurement noninvariance, as indicative of changing perceptions and reporting of symptoms, to have had any meaningful impact on trend estimates. Hence, from a measurement perspective, the notion of increased openness toward mental health problems or increased medicalization as explanations of the trends, was not supported. Thus, the trends as reported here appear to represent a real change at the latent level. In parallel, as the results of this study may be dependent on context and measurement, we hope that future trend research perform psychometric evaluations of their own, as at present, equivalence of measures seem to be largely assumed rather than empirically tested. This point is further substantiated by the finding of *noninvariance* across gender and age, as comparing trends between boys and girls and across age seem to be of interest in many contemporary trend studies.

### Implications for research and public health

The validity of trend research relies on having comparable trait estimates, which requires a minimum partial invariance across measurement occasions (Widaman, Ferrer, & Conger, 2010). Thus, assessment of factor structure and measurement invariance is a vital step to obtain valid inferences of changes across cohorts. The tenets of latent variable models are well-known within the field of psychometrics, but seem to be less well-known within many subfields of psychology (Maassen et al., 2023; McNeish & Wolf, 2020), including adolescent epidemiology (Black et al., 2024; Hagquist et al., 2017). It is beyond the scope of this study to speculate why (but see Sharpe, 2013), but it is hoped that the present study may inspire future time trend research to pay closer attention to the psychometric properties including measurement invariance of the instrument used. Ultimately, this may also be of public health utility, as valid and reliable measures are a necessity in order to inform policy, health services, and potential preventive efforts.

This study has purposefully taken a measurement approach. Following this path, it would be interesting for future studies to perform similar investigations on other measures and contexts, as it is possible that the extent to which adolescents have changed how they report symptoms differs across countries and that the sensitivity to detect such changes depends on the pool of items used. Related, studies with access to a greater pool of items could also examine, for instance, whether noninvariance or DIF effects over time are more present in items assumed to be more stigmatizing than others. There are, however, other avenues for research as well. As discussed at length by Foulkes and Andrews (2023), this may include survey research as well as controlled experiments. For example, surveys could track whether changes in mental health awareness or stigma co-vary with trends in depressive symptoms, and controlled experiments may examine the effects of exposure to mental health awareness-raising information on symptom reporting. Ideally, such work should still consider the psychometric properties of the measures used and how they work across different groups and measurement occasions.

## Supporting information

Nilsen et al. supplementary material 1 Nilsen et al. supplementary material

Nilsen et al. supplementary material 2 Nilsen et al. supplementary material

## Supplementary material

The supplementary material for this article can be found at https://doi.org/10.1017/S0033291724002447
