# Supplementary material for: Time trends in adolescent depressive symptoms from 2010 to 2019 in Norway: real increase or artifacts of measurements?
Source: Psychol Med. 2024 Oct 7;54(14):3949–61. doi: 10.1017/S0033291724002447 (PMC11578914; doi:10.1017/S0033291724002447)
Supplement: Nilsen et al. supplementary material 2 — Nilsen et al. supplementary material [file S0033291724002447sup002.pdf]

Supplementary materials

Table of Contents

Supplementary Table 1. Descriptive characteristics by gender and survey years (including those with missing on age). 4

Unidimensionality assessment **6**

Supplementary Table 2. Model fit and dynamic fit indices by survey year for boys and girls 6

Supplementary Table 3. Model fit and dynamic fit indices by age groups for boys and girls 7

Supplementary Figure 1. Unidimensionality assessment: Parallel analysis by gender (pooled across survey years). 8

Supplementary Figure 2. Unidimensionality assessment: Parallel analysis by gender and survey years 9

Supplementary Figure 3. Unidimensionality assessment: Parallel analysis by age groups and time for girls. 10

Supplementary Figure 3 Continued. Unidimensionality assessment: Parallel analysis by age groups and time for girls. 11

Supplementary Figure 4. Unidimensionality assessment: Parallel analysis by age groups and time for boys. 12

Supplementary Figure 4 Continued. Unidimensionality assessment: Parallel analysis by age groups and time for boys. 13

Measurement invariance across time **14**

Supplementary Figure 5. Factor loadings and thresholds from baseline (configural) models for boys and girls by survey year 14

Supplementary Table 4. Measurement invariance across survey years by age groups for girls 15

Supplementary Table 5. Measurement invariance across survey years by age groups for boys 16

Supplementary Table 6. Trends in latent mean scores by age groups, girls 17

Supplementary Table 7. Trends in latent mean scores by age groups, boys 18

Supplementary Table 8. Intercept means and dmacs effect size of invariance, partial invariance model girls 15 year olds. 19

Table 9. Trends in latent mean scores among 15 year old girls. Comparing models not accounting (Intercepts fixed) and accounting (Partial invariance) for non-invariant intercepts of item 6. 19

Supplementary Table 10. Intercept means and d_{MACS} effect size of invariance, partial invariance model boys 14 year olds. 19

Supplementary Table 11. Trends in latent mean scores among 14 year old boys. Comparing models not accounting (Intercepts fixed) and accounting (Partial invariance) for non-invariant intercepts of item 5. 20

Supplementary Table 12. Intercept means and d_{MACS} effect size of invariance, partial invariance model boys 15 year olds. 20

Supplementary Table 13. Trends in latent mean scores among 15 year old boys. Comparing models not accounting (Intercepts fixed) and accounting (Partial invariance) for non-invariant intercepts of item 5 20

Supplementary Table 14. Intercept means and d_{MACS} effect size of invariance, partial invariance model boys 16 year olds. 21

Supplementary Table 15. Trends in latent mean scores among 16 year old boys. Comparing models not accounting (Intercepts fixed) and accounting (Partial invariance) for non-invariant intercepts of item 5 21

Measurement invariance across age groups **22**

Supplementary Table 16. Measurement invariance across age groups among boys and girls, pooled sample (2010 – 2019). 22

Supplementary Table 17. Measurement invariance across age groups by survey years; Girls 23

Supplementary Table 18. Measurement invariance across age groups by survey years; Boys 25

Supplementary Table 19. Intercept means and d_{MACS} effect size of noninvariant item 2 and 3, partial invariance model girls pooled sample. 27

Supplementary Table 20. Comparing latent means across age groups in Girls (pooled sample). Comparing models not accounting (Intercepts fixed) and accounting for (partial invariance) non-invariant intercepts of items 2 and 3. 27

Supplementary Table 21. Intercept means and d_{MACS} effect size of noninvariant item 3, partial invariance model girls 2010. 27

Supplementary Table 22. Intercept means and d_{MACS} effect size of noninvariant item 3, partial invariance model girls 2011. 28

Supplementary Table 23. Intercept means and d_{MACS} effect size of noninvariant item 3, partial invariance model girls 2012. 28

Supplementary Table 24. Intercept means and d_{MACS} effect size of noninvariant item 2 and 3, partial invariance model girls 2013. .... *28*

Supplementary Table 25. Intercept means and d_{MACS} effect size of noninvariant item 2 and 3, partial invariance model girls 2014. .... *28*

Supplementary Table 26. Intercept means and d_{MACS} effect size of noninvariant item 2 and 3, partial invariance model girls 2015. .... *29*

Supplementary Table 27. Intercept means and d_{MACS} effect size of noninvariant item 2 and 3, partial invariance model girls 2016. .... *29*

Supplementary Table 28. Intercept means and d_{MACS} effect size of noninvariant item 2 and 3, partial invariance model girls 2017. .... *29*

Supplementary Table 29. Intercept means and d_{MACS} effect size of noninvariant item 2 and 3, partial invariance model girls 2018. .... *29*

Supplementary Table 30. Intercept means and d_{MACS} effect size of noninvariant item 2 and 3, partial invariance model girls 2019. .... *30*

Supplementary Table 31. Latent means across age groups in Girls per survey year. Comparing models not accounting (Intercepts fixed) and accounting for (partial invariance) non-invariant parameters *30*

Supplementary Figure 6. The practical consequences of noninvariance on latent mean differences across age groups in girls, by survey year *32*

Gender invariance **33**

Supplementary Table 32. Measurement invariance across gender, pooled sample (across all survey years). *33*

Supplementary Table 33. Intercept means and d_{MACS} effect size of noninvariant intercepts of item 6, partial invariance model. *33*

Supplementary Table 34. Latent means by gender (pooled sample). Comparing models not accounting (Intercepts fixed) and accounting (partial invariance) for non-invariant parameters *33*

Supplementary Table 35. Measurement invariance across gender by survey year. *34*

Supplementary Table 36. Partial gender invariance across survey years: Intercept means and d_{MACS} effect size of noninvariant intercepts *37*

Supplementary Table 37. Latent means by gender per survey year. Comparing models not accounting (Intercepts fixed) and accounting (partial invariance) for non-invariant parameters *37*

Supplementary Figure 7. The practical consequences of non-invariance on differences in latent means between boys and girls, by survey year. *38*

Sensitivity analyses **39**

Supplementary Table 38. Sensitivity analyses: Measurement invariance across time by age groups among girls when excluding year 2010 .... *39*

Supplementary Table 39. Sensitivity analyses: Measurement invariance across time by age groups among boys when excluding year 2010. .... *40*

Supplementary Table 40. Sensitivity analyses: Measurement invariance comparing 2010 and 2019 among girls. *41*

Supplementary Table 42. Sensitivity analyses: Intercept means and d_{MACS} effect size of noninvariant intercepts of item 5, partial invariance model. Comparing 2013 and 2019 among 13 and 15 year old boys. *43*

Supplementary Table 43. Sensitivity analyses: Latent means among boys between years 2013 and 2019. Comparing models not accounting (Intercepts fixed) and accounting (partial invariance) for non-invariant parameters. *43*

Missing data assessment **44**

Supplementary Table 44. Sensitivity analyses: Comparing imputed item values with observed item values across time; ***Boys****.* *45*

Supplementary Table 45. Sensitivity analyses: Comparing imputed item values with observed item values across time; ***Girls.*** *46*

Supplementary Table 46. Sensitivity analyses: Measurement invariance across time by age groups using multiple imputed data: ***Girls*** *47*

Supplementary Table 47. Sensitivity analyses: Measurement invariance across time by age groups using multiple imputed data: ***Boys*** *48*

Supplementary Figure 8. Sensitivity analyses: Trends in latent mean scores based on multiple imputed data: ***Girls*** *49*

Supplementary Figure 9. Sensitivity analyses: Trends in latent mean scores based on multiple imputed data: ***Boys*** *50*

Supplementary Table 48. Latent means across survey years by age groups in ***Girls****, from models based on multiple imputed data. Comparing models not accounting (Intercepts fixed) and accounting (partial invariance) for non-invariant parameters.51*

Supplementary Table 49. Latent means across survey years by age groups in ***Boys****, from models based on multiple imputed data. Comparing models not accounting (Intercepts fixed) and accounting (partial invariance) for non-invariant parameters.52*

References *54*

Supplementary Table 1. Descriptive characteristics by gender and survey years (including those with missing on age).

**Girls**	2010, N = 8,739	2011, N = 5,690	2012, N = 11,976	2013, N = 40,199	2014, N = 21,811	2015, N = 34,085	2016, N = 32,652	2017, N = 48,637	2018, N = 34,332	2019, N = 56,542
Age	14.40 (1.22)	14.46 (1.23)	14.74 (1.19)	14.70 (1.40)	14.89 (1.55)	15.10 (1.60)	14.93 (1.44)	15.13 (1.60)	15.24 (1.66)	15.30 (1.62)
% missing	17%	44%	(3.5%)	(1.9%)	(2.4%)	(1.4%)	(1.7%)	(2.3%)	(2.0%)	(1.8%)
1. Struggle	2.38 (0.99)	2.25 (0.96)	2.40 (0.98)	2.38 (0.98)	2.40 (1.00)	2.46 (1.02)	2.47 (1.02)	2.53 (1.01)	2.54 (1.02)	2.57 (1.01)
Skew/curtosis	0.22/-0.97	0.36/-0.80	0.16/-0.99	0.19/-0.97	0.17/-1.03	0.09/-1.11	0.08/-1.11	0.02/-1.09	0.01/-1.12	-0.03/-1.09
(% missing)	(0.5%)	(0.4%)	(0.5%)	(0.6%)	(0.6%)	(0.6%)	(0.6%)	(0.7%)	(0.9%)	(0.9%)
2. Sleep problems	2.07 (0.97)	2.02 (0.96)	2.15 (0.99)	2.11 (0.97)	2.11 (0.98)	2.12 (0.99)	2.13 (0.99)	2.18 (1.00)	2.22 (1.01)	2.27 (1.02)
Skew/curtosis	0.56/-0.68	0.61/-0.61	0.44/-0.85	0.50/-0.74	0.50/-0.77	0.49/-0.81	0.49/-0.81	0.43/-0.89	0.39/-0.96	0.34/-0.98
(% missing)	(0.3%)	(0.4%)	(0.4%)	(0.4%)	(0.4%)	(0.4%)	(0.3%)	(0.5%)	(0.6%)	(0.5%)
3. Depressed	2.03 (0.97)	2.03 (0.97)	2.12 (0.99)	2.13 (1.01)	2.08 (1.02)	2.10 (1.02)	2.11 (1.03)	2.19 (1.03)	2.21 (1.03)	2.28 (1.03)
Skew/curtosis	0.63/-0.61	0.61/-0.63	0.49/-0.82	0.49/-0.87	0.55/-0.84	0.53/-0.87	0.52/-0.90	0.40/-1.00	0.38/-1.02	0.30/-1.05
(% missing)	(0.6%)	(0.4%)	(0.5%)	(0.6%)	(0.5%)	(0.5%)	(0.6%)	(0.6%)	(0.8%)	(0.7%)
4. Hopeless	1.89 (0.99)	1.83 (0.97)	1.96 (1.01)	1.97 (1.02)	1.99 (1.04)	2.04 (1.05)	2.03 (1.06)	2.10 (1.06)	2.14 (1.07)	2.18 (1.07)
Skew/curtosis	0.83/-0.44	0.91/-0.27	0.71/-0.66	0.70/-0.71	0.66/-0.81	0.59/-0.92	0.62/-0.90	0.51/-1.01	0.45/-1.08	0.41/-1.11
(% missing)	(0.6%)	(0.7%)	(0.7%)	(0.7%)	(0.7%)	(0.7%)	(0.7%)	(0.8%)	(0.9%)	(0.9%)
5. Tense	2.01 (0.97)	1.96 (0.95)	2.04 (0.98)	2.05 (0.98)	1.97 (0.97)	2.03 (0.99)	2.03 (1.00)	2.17 (1.01)	2.18 (1.03)	2.21 (1.03)
Skew/curtosis	0.63/-0.64	0.68/-0.53	0.56/-0.74	0.54/-0.76	0.65/-0.63	0.57/-0.76	0.58/-0.78	0.40/-0.97	0.38/-1.03	0.35/-1.03
(% missing)	(0.9%)	(0.9%)	(1.1%)	(0.9%)	(0.9%)	(1.0%)	(1.0%)	(1.1%)	(1.4%)	(1.5%)
6. Worried	2.40 (1.02)	2.31 (1.00)	2.43 (1.01)	2.45 (1.02)	2.49 (1.03)	2.55 (1.04)	2.57 (1.05)	2.71 (1.02)	2.74 (1.03)	2.77 (1.02)
Skew/curtosis	0.14/-1.09	0.25/-1.01	0.08/-1.09	0.06/-1.12	0.02/-1.14	-0.05/-1.17	-0.07/-1.19	-0.23/-1.09	-0.27/-1.09	-0.30/-1.05
(% missing)	(0.5%)	(0.7%)	(0.7%)	(0.7%)	(0.6%)	(0.7%)	(0.7%)	(0.8%)	(0.9%)	(0.8%)
Sum score	2.13 (0.77)	2.07 (0.76)	2.19 (0.78)	2.18 (0.79)	2.17 (0.79)	2.22 (0.81)	2.22 (0.82)	2.31 (0.81)	2.34 (0.82)	2.38 (0.81)
Skew/curtosis	0.53/-0.45	0.63/-0.30	0.45/-0.55	0.47/-0.58	0.46/-0.62	0.40/-0.71	0.40/-0.74	0.29/-0.81	0.25/-0.83	0.21/-0.85
(% missing)	(2.3%)	(2.2%)	(2.4%)	(2.5%)	(2.4%)	(2.4%)	(2.5%)	(2.8%)	(3.2%)	(3.1%)
**Boys**	2010, N = 8,374	2011, N = 5,835	2012, N = 11,464	2013, N = 39,027	2014, N = 21,607	2015, N = 32,429	2016, N = 31,993	2017, N = 47,978	2018, N = 32,420	2019, N = 54,555
Age	14.33 (1.15)	14.51 (1.26)	14.74 (1.16)	14.68 (1.36)	14.85 (1.51)	15.07 (1.54)	14.90 (1.41)	15.05 (1.55)	15.17 (1.62)	15.26 (1.59)
(% missing)	18%	44%	(3.9%)	(2.2%)	(2.9%)	(1.7%)	(2.1%)	(2.4%)	(2.2%)	(2.0%)
1. Struggle	2.08 (0.96)	1.86 (0.88)	2.00 (0.94)	1.91 (0.90)	1.88 (0.90)	1.90 (0.92)	1.88 (0.91)	1.93 (0.93)	1.99 (0.96)	1.98 (0.95)
Skew/curtosis	0.59/-0.59	0.81/-0.06	0.65 /-0.47	0.75/-0.24	0.79/-0.20	0.76/-0.34	0.79/-0.24	0.73/-0.37	0.65/-0.56	0.66/-0.53
(% missing)	(0.5%)	(0.5%)	(0.7%)	(0.7%)	(0.6%)	(0.6%)	(0.6%)	(0.8%)	(1.0%)	(0.9%)
2. Sleep problems	1.89 (0.95)	1.76 (0.87)	1.90 (0.93)	1.82 (0.90)	1.80 (0.89)	1.83 (0.90)	1.81 (0.90)	1.88 (0.92)	1.92 (0.95)	1.95 (0.96)

Skew/curtosis	0.83/-0.29	0.98/ 0.17	0.78 /-0.33	0.88/-0.08	0.91/-0.04	0.87/-0.14	0.91/-0.03	0.80/-0.26	0.75/-0.42	0.71/-0.48
(% missing)	(0.5%)	(0.5%)	(0.6%)	(0.6%)	(0.5%)	(0.6%)	(0.4%)	(0.7%)	(0.9%)	(0.6%)
3. Depressed	1.64 (0.85)	1.54 (0.78)	1.61 (0.82)	1.58 (0.81)	1.56 (0.81)	1.59 (0.83)	1.57 (0.82)	1.68 (0.87)	1.73 (0.90)	1.77 (0.92)
Skew/curtosis	1.29/ 0.93	1.44/ 1.49	1.28 / 0.94	1.33/ 1.10	1.41/ 1.30	1.35/ 1.07	1.38/ 1.17	1.16/ 0.49	1.08/ 0.22	1.01/ 0.05
(% missing)	(0.5%)	(0.8%)	(0.8%)	(0.8%)	(0.6%)	(0.7%)	(0.7%)	(0.9%)	(1.1%)	(0.9%)
4. Hopeless	1.63 (0.88)	1.52 (0.81)	1.61 (0.86)	1.57 (0.84)	1.56 (0.85)	1.60 (0.87)	1.57 (0.85)	1.66 (0.91)	1.72 (0.94)	1.74 (0.95)
Skew/curtosis	1.30/ 0.75	1.52/ 1.53	1.33 / 0.92	1.42/ 1.18	1.43/ 1.12	1.35/ 0.88	1.42/ 1.12	1.22/ 0.46	1.11/ 0.14	1.07/ 0.04
(% missing)	(0.6%)	(0.8%)	(1.0%)	(0.9%)	(0.7%)	(0.8%)	(0.8%)	(1.0%)	(1.3%)	(1.2%)
5. Tense	1.78 (0.90)	1.64 (0.80)	1.71 (0.85)	1.68 (0.83)	1.61 (0.80)	1.62 (0.81)	1.57 (0.78)	1.67 (0.83)	1.67 (0.85)	1.69 (0.85)
Skew/curtosis	0.97/ 0.09	1.14/ 0.70	1.04 / 0.32	1.09/ 0.47	1.21/ 0.83	1.20/ 0.73	1.28/ 0.99	1.10/ 0.46	1.12/ 0.42	1.09/ 0.37
(% missing)	(0.9%)	(0.9%)	(1.1%)	(1.1%)	(1.0%)	(1.1%)	(1.1%)	(1.3%)	(1.7%)	(1.7%)
6. Worried	1.90 (0.96)	1.73 (0.88)	1.86 (0.94)	1.80 (0.91)	1.81 (0.92)	1.85 (0.95)	1.83 (0.94)	1.97 (0.98)	2.01 (1.00)	2.04 (1.01)
Skew/curtosis	0.78/-0.43	1.00/ 0.08	0.79 /-0.41	0.89/-0.17	0.89/-0.19	0.82/-0.38	0.86/-0.30	0.65/-0.67	0.60/-0.79	0.57/-0.82
(% missing)	(0.8%)	(0.8%)	(1.0%)	(1.0%)	(0.8%)	(1.0%)	(0.9%)	(1.0%)	(1.3%)	(1.1%)
Sum score	1.81 (0.71)	1.67 (0.63)	1.78 (0.68)	1.73 (0.66)	1.70 (0.65)	1.73 (0.68)	1.70 (0.66)	1.79 (0.70)	1.84 (0.72)	1.86 (0.73)
Skew/curtosis	1.00/ 0.61	1.16/ 1.16	1.00 / 0.67	1.08/ 0.89	1.14/ 1.01	1.08/ 0.77	1.12/ 0.90	0.98/ 0.44	0.91/ 0.25	0.88/ 0.16
(% missing)	(2.3%)	(2.5%)	(3.0%)	(2.7%)	(2.5%)	(2.7%)	(2.5%)	(3.0%)	(3.5%)	(3.3%)

All results presented as Mean (SD) unless otherwise stated. Items scored on a scale ranging from 1 (“not at all affected”) to 4 (“extremely affected). *Excess kurtosis shown for all items.

Unidimensionality assessment

Supplementary Table 2. Model fit and dynamic fit indices by survey year for boys and girls

		Model fit						Dynamic fit		
Model	χ^2(df)	p	CFI	RMSEA (90%CI)	TLI	SRMR	ω	CFI	RMSEA	SRMR
**Girls**										
**Overall**	**11852.091(9)**	**<.001**	**0.995**	**0.067 (0.066, 0.068)**	**0.991**	**0.020**	**0.89**	**0.995**	**0.063**	**0.022**
*	6482.76(8)	<.001	0.997	0.052 (0.051, 0.053)	0.995	0.016	0.88			
2010	351.294(9)	<.001	0.994	0.066 (0.060, 0.072)	0.991	0.021	0.88	0.995	0.061	0.023
†	203.257(8)	<.001	0.997	0.053 (0.047, 0.059)	0.994	0.017	0.88			
2011	221.507(9)	<.001	0.995	0.064 (0.057, 0.072)	0.991	0.021	0.88	0.995	0.060	0.022
†	93.881(8)	<.001	0.998	0.043 (0.036, 0.052)	0.996	0.014	0.89			
2012	522.965(9)	<.001	0.994	0.069 (0.064, 0.074)	0.990	0.022	0.88	0.995	0.061	0.022
†	340.496(8)	<.001	0.996	0.059 (0.054, 0.064)	0.993	0.017	0.88			
2013	1745.016(9)	<.001	0.995	0.069 (0.067, 0.072)	0.991	0.022	0.89	0.996	0.062	0.022
†	975.775(8)	<.001	0.997	0.055 (0.052, 0.058)	0.994	0.016	0.89			
2014	933.298(9)	<.001	0.994	0.069 (0.065, 0.072)	0.990	0.023	0.88	0.995	0.063	0.023
†	555.349(8)	<.001	0.997	0.056 (0.052, 0.060)	0.993	0.019	0.88			
2015	1088.173(9)	<.001	0.996	0.059 (0.056, 0.062)	0.993	0.018	0.89	0.995	0.064	0.022
*	634.717(8)	<.001	0.998	0.048 (0.045, 0.051)	0.996	0.015	0.88			
2016	1261.531(9)	<.001	0.995	0.065 (0.062, 0.068)	0.992	0.020	0.89	0.995	0.063	0.022
*	612.976(8)	<.001	0.998	0.048 (0.045, 0.051)	0.996	0.015	0.88			
2017	2072.832(9)	<.001	0.995	0.069 (0.066, 0.071)	0.991	0.021	0.89	0.995	0.062	0.022
*	996.120(8)	<.001	0.997	0.050 (0.048, 0.053)	0.995	0.015	0.88			
2018	1540.722(9)	<.001	0.994	0.070 (0.067, 0.073)	0.990	0.022	0.88	0.995	0.062	0.022
*	740.030(8)	<.001	0.997	0.052 (0.049, 0.055)	0.995	0.016	0.87			
2019	2536.328(9)	<.001	0.994	0.070 (0.068, 0.073)	0.990	0.021	0.88	0.995	0.063	0.022
*	1196.328(8)	<.001	0.997	0.051 (0.049, 0.054)	0.995	0.015	0.87			
**Boys**										
**Overall**	**10554.007(9)**	**<.001**	**0.994**	**0.064 (0.063, 0.065)**	**0.989**	**0.023**	**0.87**	**0.995**	**0.058**	**0.024**
*	6300.689(8)		0.996	0.052 (0.051, 0.054)	0.993	0.018	0.86			
2010	425.104 (9)	<.001	0.992	0.074 (0.068, 0.080)	0.987	0.026	0.87	0.996	0.055	0.021
†	294.625(8)	<.001	0.995	0.065 (0.059, 0.072)	0.990	0.020	0.86			
2011	187.567 (9)	<.001	0.994	0.058 (0.051, 0.066)	0.989	0.024	0.85	0.994	0.057	0.026
†	139.12(8)	<.001	0.995	0.053 (0.045, 0.061)	0.991	0,019	0.84			
2012	650.167 (9)	<.001	0.990	0.079 (0.074, 0.084)	0.984	0.027	0.87	0.995	0.056	0.023
*	426.545(8)	<.001	0.994	0.068 (0.062, 0.073)	0.988	0,022	0.86			
2013	1785.844 (9)	<.001	0.991	0.071 (0.068, 0.074)	0.986	0.026	0.86	0.994	0.058	0.024
*	1066.322(8)	<.001	0.995	0.058 (0.055, 0.061)	0.990	0.020	0.85			
2014	757.319 (9)	<.001	0.993	0.062 (0.058, 0.066)	0.989	0.024	0.86	0.994	0.057	0.025
*	433.887(8)	<.001	0.996	0.050 (0.046, 0.054)	0,993	0.018	0.85			
2015	1092.52 (9)	<.001	0.994	0.061 (0.058, 0.064)	0.990	0.022	0.87	0.995	0.057	0.024
*	688.176(8)	<.001	0.996	0.051 (0.048, 0.054)	0.993	0.018	0.85			
2016	1066.447 (9)	<.001	0.994	0.061 (0.058, 0.064)	0.989	0.023	0.86	0.994	0.055	0.025
*	680.008(8)	<.001	0.996	0.051 (0.048, 0.055)	0.992	0.019	0.86			
2017	1679.465 (9)	<.001	0.994	0.062 (0.060, 0.065)	0.990	0.022	0.87	0.994	0.060	0.025
*	913.915(8)	<.001	0.997	0.049 (0.046, 0.051)	0.994	0.017	0.86			
2018	1120.005 (9)	<.001	0.994	0.062 (0.059, 0.065)	0.991	0.021	0.87	0.995	0.060	0.024
*	658.013(8)	<.001	0.997	0.050 (0.047, 0.053)	0.994	0.016	0.86			
2019	1968.673 (9)	<.001	0.994	0.063 (0.061, 0.066)	0.991	0.022	0.87	0.995	0.060	0.023
*	1100.093(8)	<.001	0.997	0.050 (0.048, 0.053)	0.994	0.016	0.86			

Note. χ^2= chi-square goodness-of-fit based on the Satorra-Bentler correction; df = degrees of freedom; CFI; comparative fit index; RMSEA = root mean square error of approximation; CI = confidence interval. TLI = Tucker Lewis Index. ω = coefficient omega. † Models accounting for one correlated error term between items 3 and 5 (girls), and items 2 and 5 (boys). * Models accounting for one correlated error term between item 3 and 4 (both boys and girls).

Supplementary Table 3. Model fit and dynamic fit indices by age groups for boys and girls

	Model fit							Dynamic fit		
Model	χ^2(df)	p	CFI	RMSEA (90%CI)	TLI	SRMR	ω	CFI	RMSEA	SRMR
**Boys**										
13 years	1679.817(9)	<.001	0.994	0.056(0.054, 0.059)	0.989	0.023	0.85	0.994	0.053	0.025
*	1065.441(8)	<.001	0.996	0.048(0.045, 0.050)	0.992	0.018	0.84			
14 years	2119.231(9)	<.001	0.992	0.064(0.062, 0.066)	0.987	0.025	0.86	0.994	0.056	0.025
*	1272.206(8)	<.001	0.995	0.053(0.050, 0.055)	0.992	0.019	0.85			
15 years	2207.311(9)	<.001	0.993	0.065(0.063, 0.067)	0.988	0.024	0.86	0.994	0.056	0.025
*	1199.486(8)	<.001	0.996	0.051(0.048, 0.053)	0.993	0.018	0.85			
16 years	2301.157(9)	<.001	0.993	0.071(0.068, 0.073)	0.988	0.024	0.87	0.995	0.060	0.024
*	1374.492(8)	<.001	0.996	0.058(0.055, 0.060)	0.992	0.019	0.86			
17-18 years	2213.012(9)	<.001	0.994	0.069(0.067, 0.072)	0.990	0.023	0.88	0.995	0.062	0.024
*	1402.687(8)	<.001	0.996	0.058(0.056, 0.061)	0.993	0.018	0.87			
**Girls**										
13 years	1832.671(9)	<.001	0.996	0.058(0.056, 0.060)	0.993	0.019	0.88	0.995	0.060	0.022
*	994.05(8)	<.001	0.998	0.045(0.043, 0.048)	0.996	0.014	0.87			
14 years	2269.207(9)	<.001	0.995	0.066(0.063, 0.068)	0.991	0.02	0.88	0.995	0.062	0.022
*	1126.466(8)	<.001	0.997	0.049(0.047, 0.051)	0.995	0.015	0.87			
15 years	2592.209(9)	<.001	0.994	0.070(0.068, 0.072)	0.990	0.022	0.88	0.995	0.063	0.022
*	1252.386(8)	<.001	0.997	0.051(0.049, 0.054)	0.994	0.016	0.87			
16 years	2615.073(9)	<.001	0.993	0.075(0.073, 0.078)	0.989	0.023	0.88	0.994	0.067	0.024
*	1382.58(8)	<.001	0.996	0.058(0.055, 0.061)	0.993	0.018	0.87			
17-18 years	2889.075(9)	<.001	0.993	0.075(0.073, 0.077)	0.988	0.024	0.88	0.994	0.066	0.024
*	1751.782(8)	<.001	0.996	0.062(0.060, 0.064)	0.992	0.019	0.87			

Note. χ^2= chi-square goodness-of-fit based on the Satorra-Bentler correction; df = degrees of freedom; CFI; comparative fit index; RMSEA = root mean square error of approximation; CI = confidence interval. TLI = Tucker Lewis Index. SRMR = standardized root mean squared residual; ω = omega coefficient. *Models accounting for one correlated error term between item 3 and 4. Dynamic fit: Dynamic fit cut-offs based on Level 1.

Supplementary Figure 1. Unidimensionality assessment: Parallel analysis by gender (pooled across survey years).

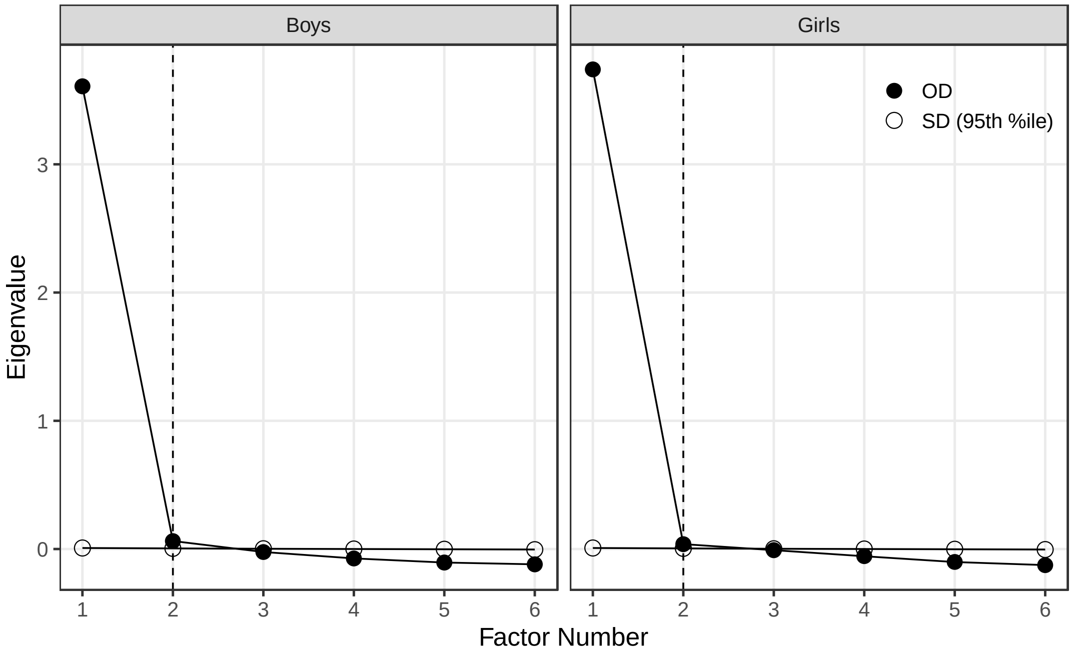

Note. OD = Observed data. SD = Simulated data. The dotted vertical line shows the recommended factors based on the parallel analyses.

Supplementary Figure 2. Unidimensionality assessment: Parallel analysis by gender and survey years

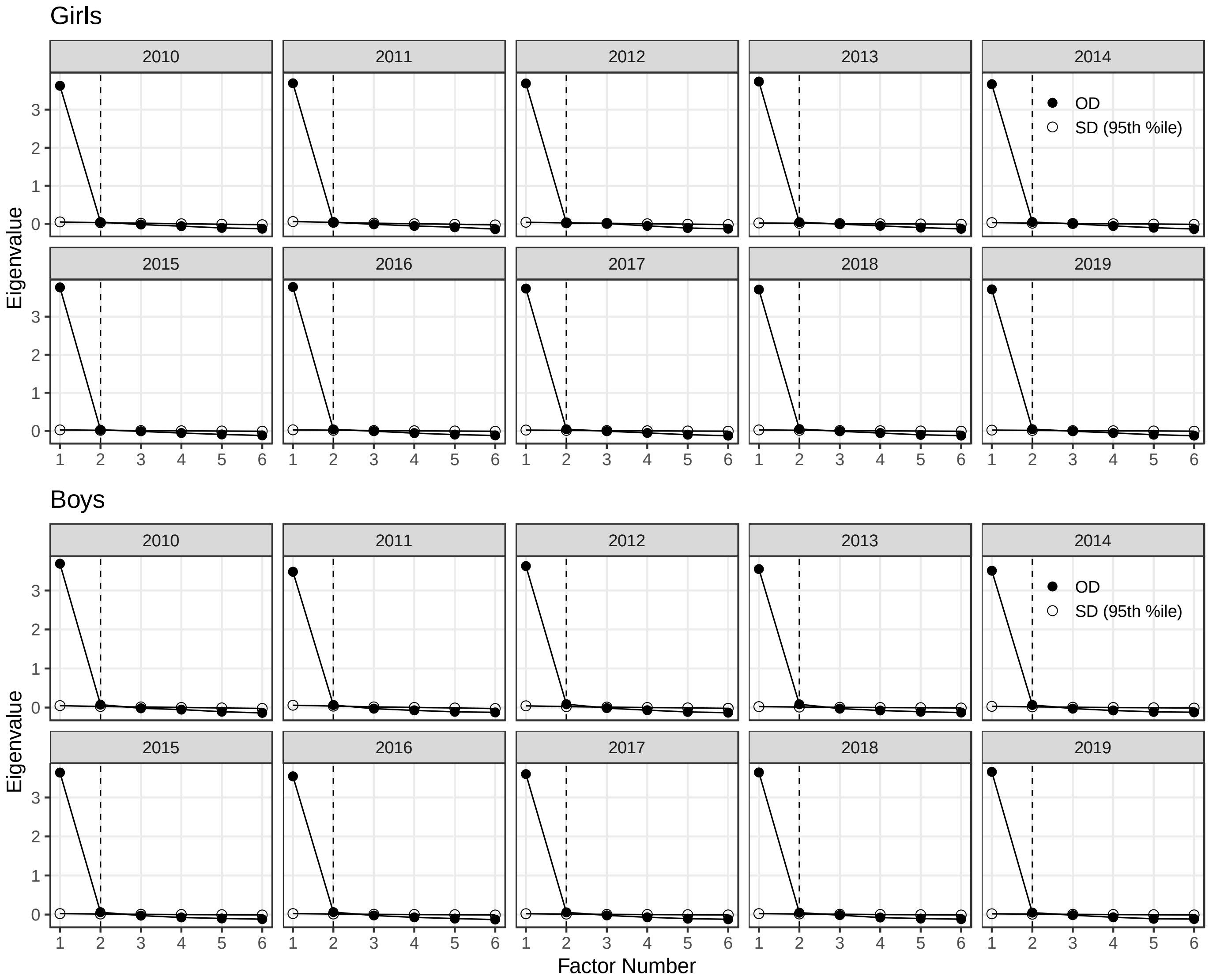

Note. OD = Observed data. SD = Simulated data. The dotted vertical line shows the recommended factors based on the parallel analyses.

Supplementary Figure 3. Unidimensionality assessment: Parallel analysis by age groups and time for girls.

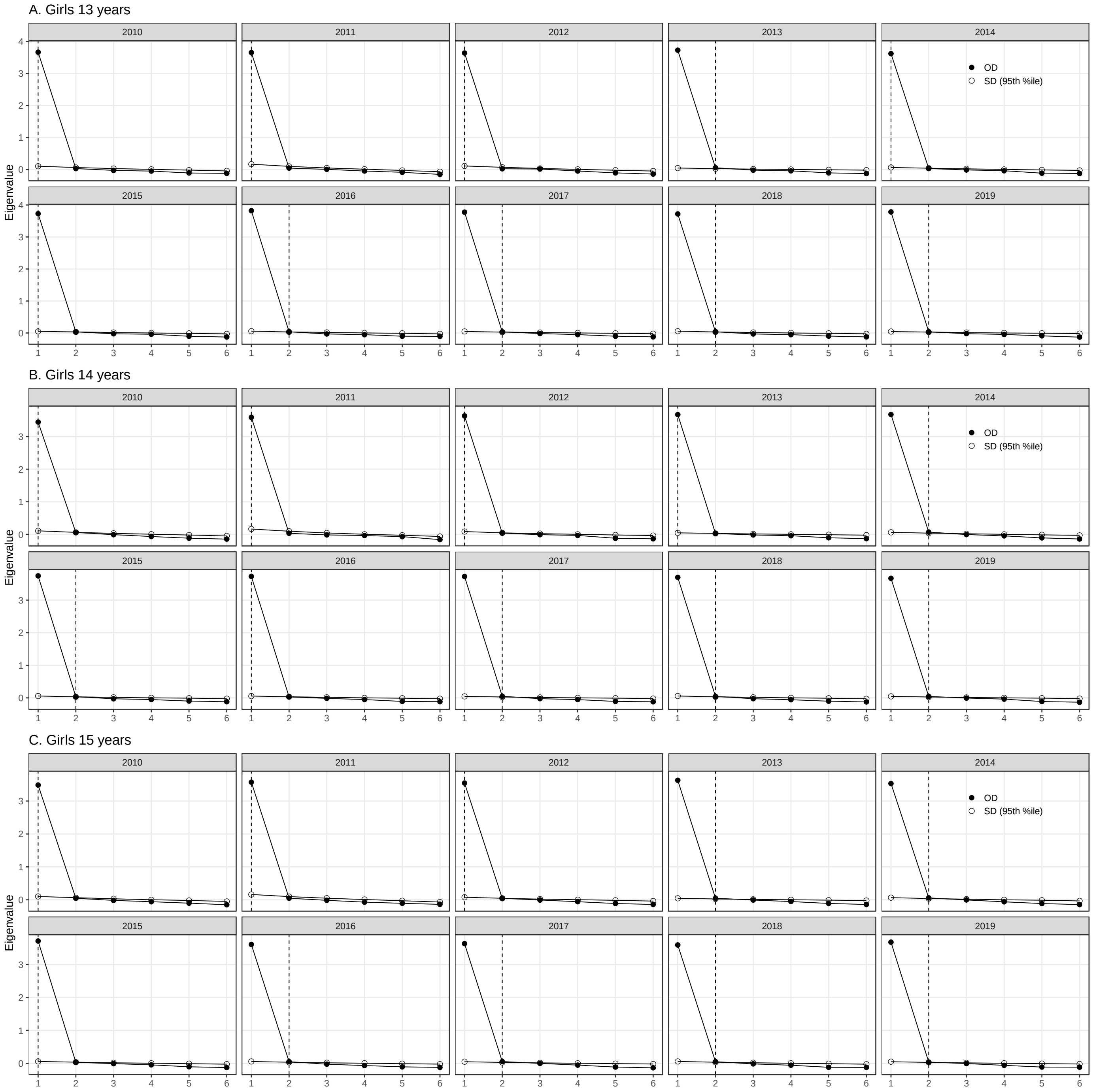

Supplementary Figure 3 Continued. Unidimensionality assessment: Parallel analysis by age groups and time for girls.

D. Girls 16 years

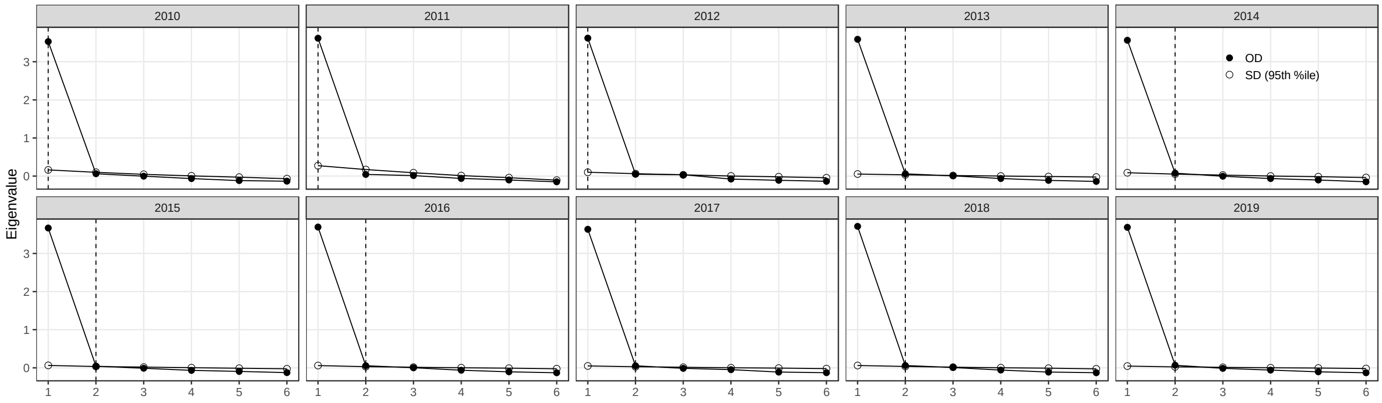

E. Girls 17−18 years

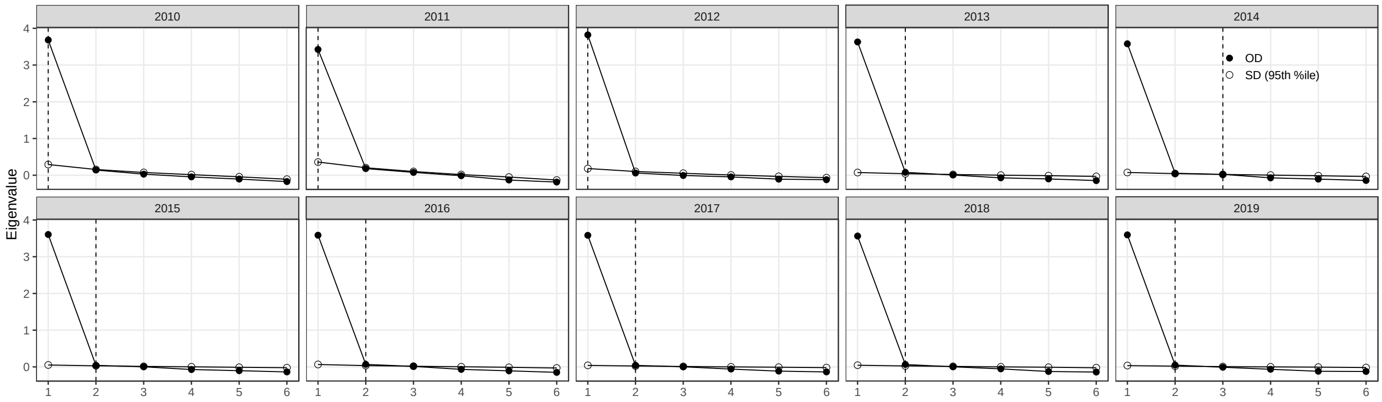

Note. OD = Observed data. SD = Simulated data. The dotted vertical line shows the recommended factors based on the parallel analyses.

Supplementary Figure 4. Unidimensionality assessment: Parallel analysis by age groups and time for boys.

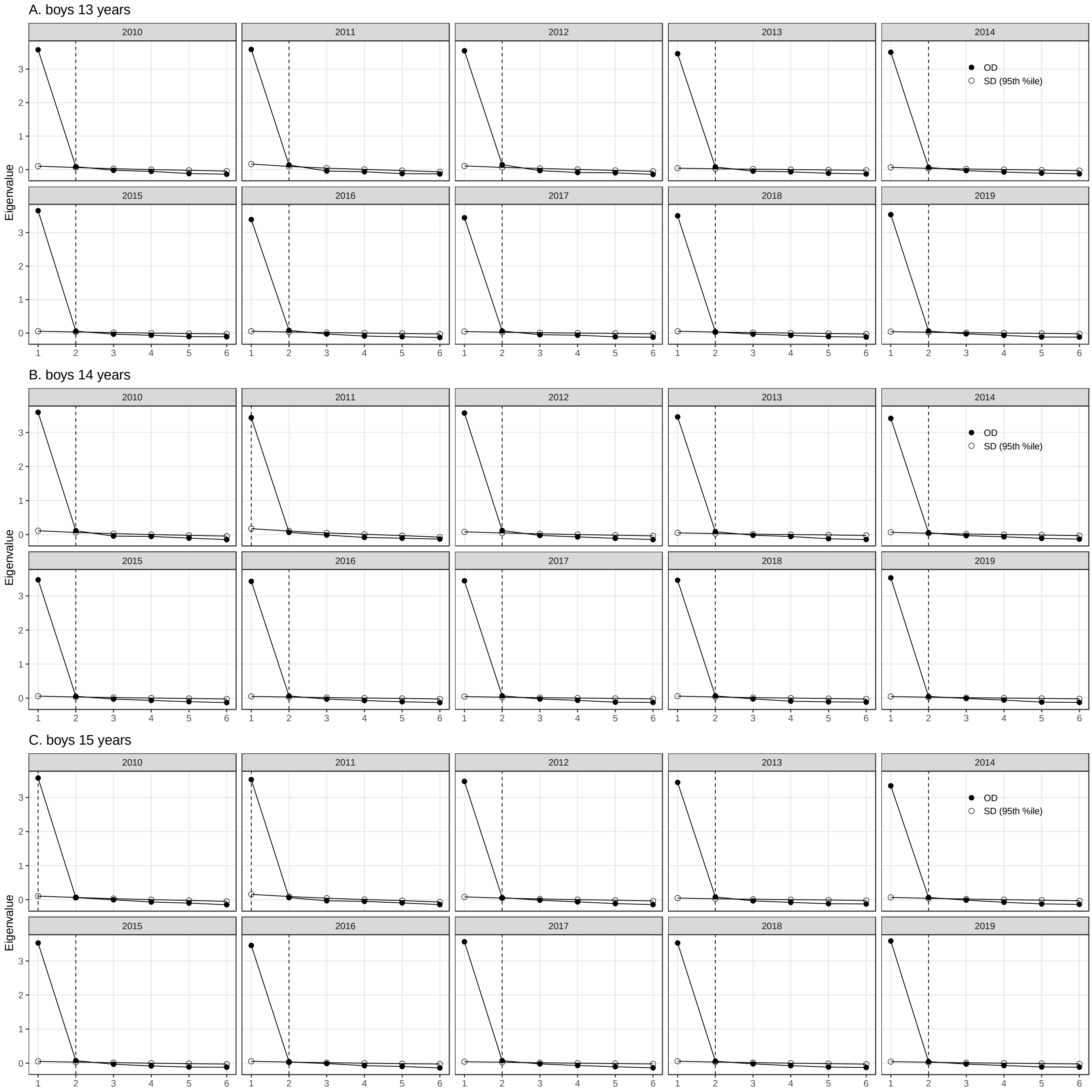

Supplementary Figure 4 Continued. Unidimensionality assessment: Parallel analysis by age groups and time for boys.

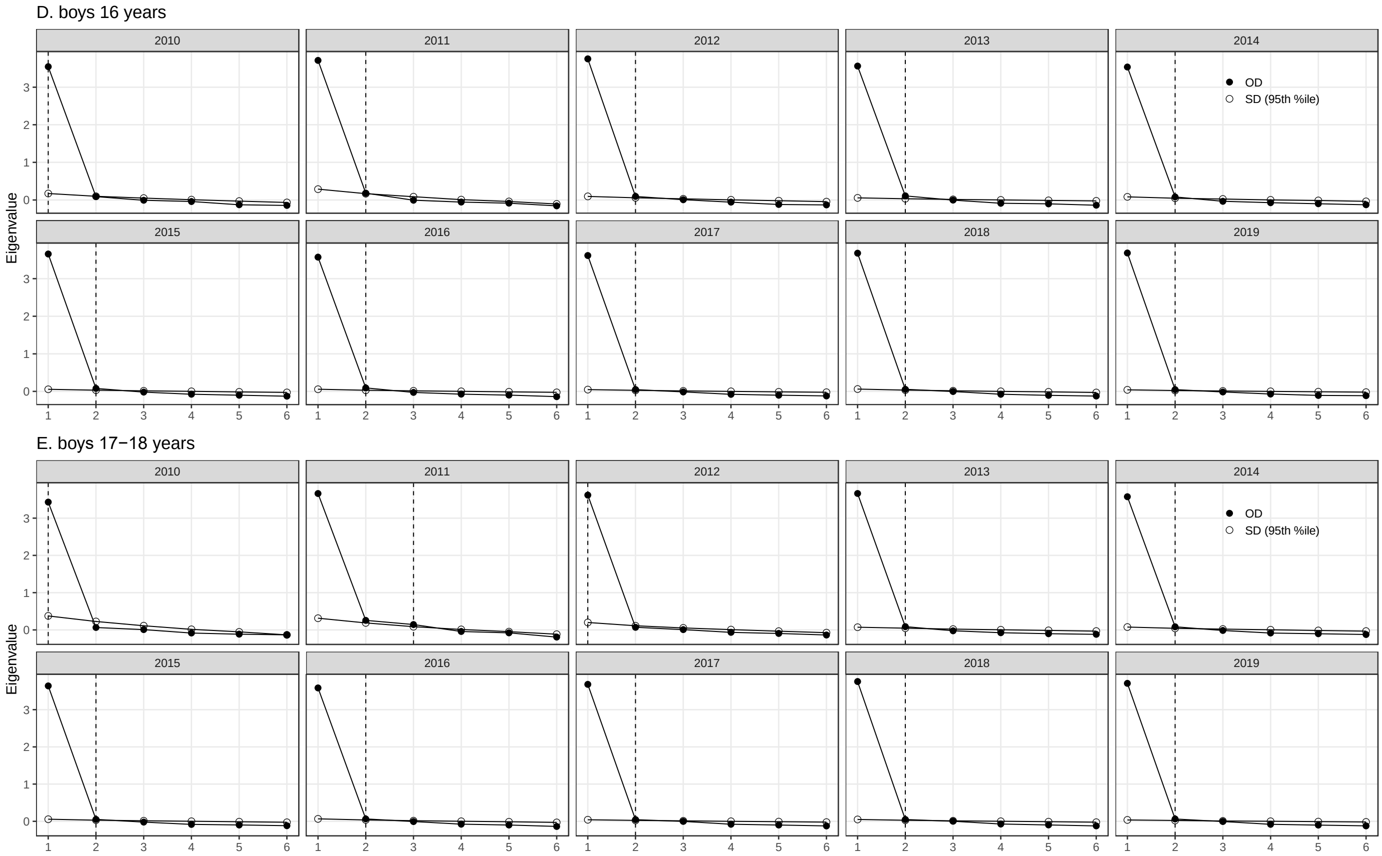

Note. OD = Observed data. SD = Simulated data. The dotted vertical line shows the recommended factors based on the parallel analyses.

Measurement invariance across time

Supplementary Figure 5. Factor loadings and thresholds from baseline (configural) models for boys and girls by survey year

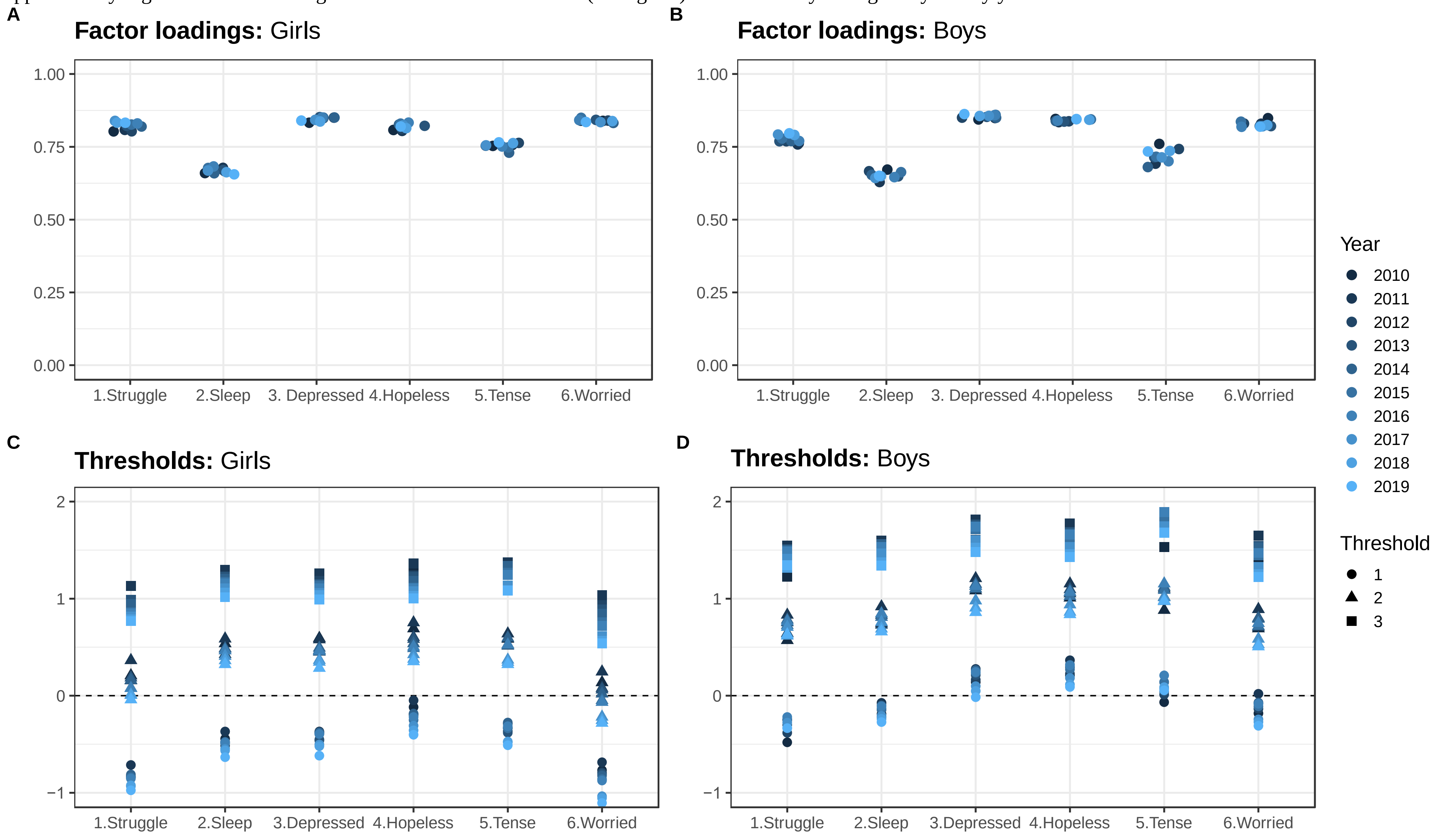

Note. This figure shows the factor loadings (**Fig. A – B**) and thresholds (**Fig C-D**) from configural MG-CFA analyses by survey year, separately for girls and boys.

Supplementary Table 4. Measurement invariance across survey years by age groups for girls

Model	$\chi2$ (df)	p	CFI	RMSEA (90 %CI)	TLI	SRMR	ΔCFI	ΔRMSEA
Age 13								
Baseline	1977.063 (90)	<.001	0.996	0.059 (0.057, 0.061)	0.993	0.020		
Baseline*	1122.017 (80)	<.001	0.998	0.047 (0.044, 0.049)	0.995	0.015		
Thresholds	1245.462 (134)	<.001	0.997	0.037 (0.035, 0.039)	0.997	0.015	-0.00019	-0.00914
Thresholds and loadings	1093.148 (179)	<.001	0.998	0.029 (0.028, 0.031)	0.998	0.015	0.00046	-0.00801
Thresholds, loadings, intercepts	1823.086 (224)	<.001	0.996	0.034 (0.033, 0.036)	0.998	0.016	-0.00159	0.00532
Age 14								
Baseline	2421.982 (90)	<.001	0.995	0.067 (0.064, 0.069)	0.991	0.021		
Baseline*	1248.336 (80)	<.001	0.997	0.050 (0.048, 0.052)	0.995	0.016		
Thresholds	1353.305 (134)	<.001	0.997	0.039 (0.038, 0.041)	0.997	0.016	-0.00012	-0.01053
Thresholds and loadings	1194.129 (179)	<.001	0.998	0.031 (0.029, 0.033)	0.998	0.016	0.00048	-0.00831
Thresholds, loadings, intercepts	1909.777 (224)	<.001	0.996	0.036 (0.034, 0.037)	0.997	0.016	-0.00158	0.00473
Age 15								
Baseline	2732.596 (90)	<.001	0.994	0.071 (0.068, 0.073)	0.989	0.023		
Baseline*	1384.154 (80)	<.001	0.997	0.053 (0.050, 0.055)	0.994	0.017		
Thresholds	1491.967 (134)	<.001	0.997	0.041 (0.040, 0.043)	0.996	0.017	-0.00013	-0.01113
Thresholds and loadings	1250.638 (179)	<.001	0.997	0.032 (0.030, 0.034)	0.998	0.017	0.00069	-0.0096
Thresholds, loadings, and intercepts	2267.667 (224)	<.001	0.995	0.039 (0.038, 0.041)	0.997	0.017	-0.00235	0.00747
Partial scalar invariance (free item 6 intercepts)	1661.05 (215)	<.001	0.997	0.034 (0.032, 0.035)	0.998	0.017	-0.0009	0.00191
Age 16								
Baseline girls	2740.275 (90)	<.001	0.993	0.076 (0.073, 0.078)	0.988	0.024		
Baseline*	1497.867 (80)	<.001	0.996	0.059 (0.056, 0.061)	0.993	0.018		
Thresholds	1580.27 (134)	<.001	0.996	0.046 (0.044, 0.048)	0.996	0.018	-0.00007	-0.01293
Thresholds and loadings	1390.287 (179)	<.001	0.997	0.036 (0.035, 0.038)	0.997	0.018	0.00061	-0.00956
Thresholds, loadings, and intercepts	2062.005 (224)	<.001	0.995	0.04 (0.038, 0.042)	0.997	0.019	-0.0014	0.00368
Age 17-18								
Baseline	2975.799 (90)	<.001	0.993	0.075 (0.073, 0.078)	0.988	0.024		
Baseline*	1840.861 (80)	<.001	0.996	0.062 (0.060, 0.065)	0.992	0.020		
Thresholds	1912.38 (134)	<.001	0.996	0.048 (0.046, 0.050)	0.995	0.020	-0.00005	-0.01392
Thresholds and loadings	1588.759 (179)	<.001	0.996	0.037 (0.036, 0.039)	0.997	0.020	0.00093	-0.01111
Thresholds, loadings, and intercepts	2195.11 (224)	<.001	0.995	0.039 (0.038, 0.041)	0.997	0.020	-0.00141	0.00213

Note. χ^2= chi-square goodness-of-fit based on the Satorra-Bentler correction; df = degrees of freedom; CFI; comparative fit index; RMSEA = root mean square error of approximation; CI = confidence interval. TLI = Tucker Lewis Index. SRMR = standardized root mean squared residual; **Δ**CFI/RMSEA = Change in CFI/RMSEA between models.
* Models accounting for one correlated error term between item 3 and 4.

Supplementary Table 5. Measurement invariance across survey years by age groups for boys

**Model**	χ2 (df)	p	CFI	RMSEA (90%CI)	TLI	SRMR	ΔCFI	ΔRMSEA
Age 13								
Baseline	1830.49121(90)	<.001	0.993	0.058 (0.055, 0.06)	0.989	0.024		
Baseline*	1194.12835(80)	<.001	0.996	0.049 (0.046, 0.051)	0.992	0.019		
Thresholds	1279.54843(134)	<.001	0.996	0.038 (0.036, 0.04)	0.995	0.019	-0.0001	-0.0106
Thresholds and loadings	1109.90592(179)	<.001	0.996	0.030 (0.028, 0.032)	0.997	0.019	0.0008	-0.0084
Thresholds, loadings, intercepts	1585.79685(224)	<.001	0.995	0.032 (0.031, 0.034)	0.996	0.020	-0.0017	0.0024
Age 14								
Baseline	2236.29542(90)	<.001	0.992	0.065 (0.062, 0.067)	0.987	0.025		
Baseline*	1369.04885(80)	<.001	0.995	0.053 (0.051, 0.056)	0.991	0.020		
Thresholds	1423.88596(134)	<.001	0.995	0.041 (0.039, 0.043)	0.995	0.020	<0.0001	-0.012
Thresholds and loadings	1217.20017(179)	<.001	0.996	0.032 (0.03, 0.034)	0.997	0.020	0.0009	-0.0092
Thresholds, loadings, and intercepts	1831.38844(224)	<.001	0.994	0.035 (0.034, 0.037)	0.996	0.021	-0.0021	0.0036
Partial scalar invariance (free item 5 intercepts)	1574.38411(215)	<.001	0.995	0.033 (0.032, 0.035)	0.997	0.020	-0.0012	0.0014
Age 15								
Baseline	2321.55045(90)	<.001	0.993	0.065 (0.063, 0.068)	0.988	0.024		
Baseline*	1306.14522(80)	<.001	0.996	0.051 (0.049, 0.054)	0.993	0.018		
Thresholds	1370.27339(134)	<.001	0.996	0.040 (0.038, 0.042)	0.996	0.018	<0.0001	-0.0115
Thresholds and loadings	1181.36749(179)	<.001	0.997	0.031 (0.029, 0.033)	0.997	0.018	0.0007	-0.0088
Thresholds, loadings, and intercepts	1997.50213(224)	<.001	0.994	0.037 (0.035, 0.038)	0.996	0.019	-0.0025	0.0059
Partial scalar invariance (free item 5 intercepts)	1556.61705(215)	<.001	0.996	0.033 (0.031, 0.034)	0.997	0.019	-0.0011	0.0017
Age 16								
Baseline	2433.69628(90)	<.001	0.993	0.071 (0.069, 0.074)	0.988	0.025		
Baseline*	1502.86096(80)	<.001	0.996	0.059 (0.056, 0.062)	0.992	0.020		
Thresholds	1580.93812(134)	<.001	0.996	0.046 (0.044, 0.048)	0.995	0.020	-0.0001	-0.013
Thresholds and loadings	1327.64316(179)	<.001	0.996	0.035 (0.034, 0.037)	0.997	0.020	0.0009	-0.0105
Thresholds, loadings, and intercepts	2040.22483(224)	<.001	0.994	0.040 (0.038, 0.041)	0.996	0.020	-0.0020	0.0044
Partial scalar invariance (free item 5 intercepts)	1679.04996(215)	<.001	0.996	0.037 (0.035, 0.038)	0.997	0.020	-0.0010	0.0011
Age 17-18								
Baseline	2265.54813(90)	<.001	0.994	0.069 (0.066, 0.071)	0.990	0.024		
Baseline*	1457.56551(80)	<.001	0.996	0.058 (0.056, 0.061)	0.993	0.019		
Thresholds	1471.78705(134)	<.001	0.996	0.044 (0.042, 0.046)	0.996	0.019	0.0001	-0.0139
Thresholds and loadings	1285.42899(179)	<.001	0.997	0.035 (0.033, 0.037)	0.997	0.019	0.0007	-0.0094
Thresholds, loadings, intercepts	1605.592(224)	<.001	0.996	0.035 (0.033, 0.036)	0.997	0.019	-0.0008	<0.0001

Note. χ^2= chi-square goodness-of-fit based on the Satorra-Bentler correction; df = degrees of freedom; CFI; comparative fit index; RMSEA = root mean square error of approximation; CI = confidence interval. TLI = Tucker Lewis Index. SRMR = standardized root mean squared residual; ΔCFI/RMSEA = Change in CFI/RMSEA between models.
* Models accounting for one correlated error term between item 3 and 4.

Supplementary Table 6. Trends in latent mean scores by age groups, girls

Year	β (95% CI)	p	β (95% CI)	p
	13-year-olds		14-year-olds	
2010	-0.046 (-0.102, 0.011)	0.113	0.021 (-0.037, 0.079)	0.480
2011	-0.053 (-0.133, 0.027)	0.193	-0.110 (-0.189, -0.032)	0.006
2012	-0.055 (-0.114, 0.005)	0.071	-0.029 (-0.077, 0.02)	0.244
2013	-0.010 (-0.048, 0.027)	0.585	0.054 (0.015, 0.092)	0.006
**2014**	**ref.**		**ref.**	
2015	-0.044 (-0.083, -0.005)	0.027	0.016 (-0.023, 0.055)	0.422
2016	-0.040 (-0.079, -0.002)	0.039	0.002 (-0.037, 0.042)	0.915
2017	0.123 (0.086, 0.161)	<0.001	0.195 (0.158, 0.232)	<0.001
2018	0.176 (0.136, 0.216)	<0.001	0.182 (0.143, 0.222)	<0.001
2019	0.282 (0.246, 0.319)	<0.001	0.238 (0.200, 0.275)	<0.001
	15-year-olds*		16-year-olds	
2010	-0.030 (-0.088, 0.028)	0.315	0.093 (0.012, 0.174)	0.024
2011	-0.111 (-0.190, -0.032)	0.006	-0.106 (-0.227, 0.014)	0.084
2012	0.008 (-0.042, 0.057)	0.764	0.071 (0.012, 0.13)	0.019
2013	0.044 (0.005, 0.083)	0.028	0.046 (-0.001, 0.093)	0.056
**2014**	**ref.**		**ref.**	
2015	0.053 (0.013, 0.093)	0.010	0.059 (0.012, 0.105)	0.013
2016	0.037 (-0.003, 0.077)	0.073	0.096 (0.051, 0.141)	<0.001
2017	0.123 (0.085, 0.161)	<0.001	0.176 (0.131, 0.221)	<0.001
2018	0.131 (0.089, 0.172)	<0.001	0.174 (0.128, 0.219)	<0.001
2019	0.141 (0.103, 0.178)	<0.001	0.200 (0.157, 0.242)	<0.001
	17-18-year-olds		Overall (13-18-year-olds)	
2010	0.091 (-0.0280, 0.210)	0.135	-0.049 (-0.078, -0.021)	0.001
2011	-0.257 (-0.405, -0.109)	0.001	-0.147 (-0.187, -0.106)	<0.001
2012	-0.047 (-0.130, 0.035)	0.263	0.015 (-0.009, 0.04)	0.225
2013	0.037 (-0.010, 0.084)	0.127	0.013 (-0.005, 0.031)	0.153
**2014**	**ref.**		**ref.**	
2015	0.083 (0.041, 0.125)	<0.001	0.057 (0.039, 0.075)	<0.001
2016	0.156 (0.111, 0.202)	<0.001	0.062 (0.044, 0.081)	<0.001
2017	0.199 (0.158, 0.239)	<0.001	0.190 (0.173, 0.208)	<0.001
2018	0.242 (0.201, 0.284)	<0.001	0.219 (0.2, 0.237)	<0.001
2019	0.286 (0.248, 0.325)	<0.001	0.278 (0.261, 0.295)	<0.001

Note. This table shows latent mean depressive symptom scores by age groups among boys. Ref. = reference group (survey year 2014). β = standardized regression coefficient. 95% CI =95 % confidence intervals of β .
*Partial scalar invariance models.

Supplementary Table 7. Trends in latent mean scores by age groups, boys

Year	β (95% CI)	p	β (95% CI)	p
	13-year-olds		14-year-olds*	
2010	0.172 (0.106, 0.238)	<0.001	0.239 (0.174, 0.303)	<0.001
2011	0.059 (-0.033, 0.151)	0.210	-0.026 (-0.116, 0.063)	0.567
2012	0.013 (-0.057, 0.084)	0.708	0.136 (0.081, 0.190)	<0.001
2013	0.027 (-0.017, 0.072)	0.231	0.070 (0.027, 0.114)	0.002
**2014**	**ref.**		**ref.**	
2015	-0.034 (-0.080, 0.013)	0.155	-0.018 (-0.062, 0.027)	0.437
2016	-0.083 (-0.130, -0.035)	0.001	-0.004 (-0.049, 0.040)	0.846
2017	0.131 (0.087, 0.175)	<0.001	0.158 (0.116, 0.199)	<0.001
2018	0.147 (0.101, 0.194)	<0.001	0.196 (0.151, 0.241)	<0.001
2019	0.230 (0.187, 0.273)	<0.001	0.194 (0.153, 0.234)	<0.001
	15-year-olds*		16-year-olds*	
2010	0.239 (0.177, 0.302)	<0.001	0.206 (0.117, 0.295)	<0.001
2011	-0.038 (-0.119, 0.042)	0.351	0.039 (-0.092, 0.169)	0.560
2012	0.138 (0.086, 0.191)	<0.001	0.185 (0.122, 0.249)	<0.001
2013	0.031 (-0.01, 0.073)	0.136	0.083 (0.034, 0.133)	0.001
**2014**	**ref.**		**ref.**	
2015	0.025 (-0.018, 0.068)	0.258	0.039 (-0.01, 0.087)	0.117
2016	-0.035 (-0.078, 0.007)	0.104	0.038 (-0.011, 0.086)	0.125
2017	0.129 (0.09, 0.169)	<0.001	0.128 (0.082, 0.173)	<0.001
2018	0.161 (0.118, 0.204)	<0.001	0.187 (0.139, 0.236)	<0.001
2019	0.190 (0.151, 0.229)	<0.001	0.196 (0.152, 0.239)	<0.001
	17-18-year-olds		Overall (13-18-year-olds)	
2010	0.308 (0.140, 0.476)	<0.001	0.169 (0.14, 0.199)	<0.001
2011	-0.146 (-0.281, -0.012)	0.033	-0.032 (-0.066, 0.002)	0.068
2012	-0.034 (-0.128, 0.061)	0.487	0.133 (0.106, 0.16)	<0.001
2013	0.089 (0.037, 0.14)	0.001	0.052 (0.032, 0.071)	<0.001
**2014**	**ref.**		**ref.**	
2015	0.043 (-0.002, 0.088)	0.063	0.036 (0.016, 0.056)	<0.001
2016	0.030 (-0.019, 0.079)	0.231	-0.004 (-0.024, 0.016)	0.689
2017	0.130 (0.088, 0.173)	<0.001	0.148 (0.13, 0.167)	<0.001
2018	0.185 (0.142, 0.227)	<0.001	0.196 (0.176, 0.215)	<0.001
2019	0.201 (0.161, 0.24)	<0.001	0.233 (0.215, 0.251)	<0.001

Note. This table shows latent mean depressive symptom scores by age groups among boys. Ref. = reference group (survey year 2014). β = standardized regression coefficient. 95% CI =95 % confidence intervals of β . *Partial scalar invariance models.

Supplementary Table 8. Intercept means and dmacs effect size of invariance, partial invariance model girls 15 year olds.

Year	Intercept mean (95% CI)	d_{MACS}
2010	ref.	ref.
2011	-0.019 (-0.059, 0.021)	0.048
2012	-0.066 (-0.119, -0.013)	0.057
2013	-0.077 (-0.111, -0.043)	0.045
2014	-0.065 (-0.092, -0.038)	0.019
2015	0.020 (-0.010, 0.049)	0.037
2016	0.089 (0.059, 0.119)	0.102
2017	0.104 (0.076, 0.132)	0.117
2018	0.124 (0.093, 0.155)	0.134
2019	0.116 (0.088, 0.144)	0.127

Table 9. Trends in latent mean scores among 15 year old girls. Comparing models not accounting (Intercepts fixed) and accounting (Partial invariance) for non-invariant intercepts of item 6.

	Intercepts fixed		Partial invaraince*	
Year	β (95% CI)	p	β (95% CI)	p
2010	-0.035 (-0.092, 0.022)	0.227	-0.030 (-0.088, 0.028)	0.315
2011	-0.129 (-0.207, -0.051)	0.001	-0.111 (-0.19, -0.032)	0.006
2012	-0.013 (-0.061, 0.035)	0.594	0.008 (-0.042, 0.057)	0.764
2014	**ref.**		**ref.**	
2013	0.027 (-0.011, 0.065)	0.159	0.044 (0.005, 0.083)	0.028
2015	0.058 (0.018, 0.097)	0.004	0.053 (0.013, 0.093)	0.010
2016	0.058 (0.019, 0.098)	0.004	0.037 (-0.003, 0.077)	0.073
2017	0.148 (0.111, 0.186)	<0.001	0.123 (0.085, 0.161)	<0.001
2018	0.160 (0.119, 0.201)	<0.001	0.131 (0.089, 0.172)	<0.001
2019	0.168 (0.131, 0.205)	<0.001	0.141 (0.103, 0.178)	<0.001

Note. Ref. = reference group (survey year 2014). β = standardized regression coefficient. 95% CI =95 % confidence intervals of β .

Supplementary Table 10. Intercept means and d_{MACS} effect size of invariance, partial invariance model boys 14 year olds.

Year	intercept mean (95% CI)	d_{MACS}
2010	**ref.**	**ref**.
2011	0.109 (0.057, 0.161)	0.060
2012	0.029 (-0.049, 0.108)	0.069
2013	0.039 (-0.007, 0.085)	0.022
2014	0.081 (0.043, 0.119)	0.080
2015	-0.007 (-0.048,0.035)	0.098
2016	-0.092 (-0.136,-0.047)	0.171
2017	-0.025 (-0.064,0.013)	0.123
2018	-0.079 (-0.121,-0.037)	0.175
2019	-0.087 (-0.126,-0.048)	0.184

Supplementary Table 11. Trends in latent mean scores among 14 year old boys. Comparing models not accounting (Intercepts fixed) and accounting (Partial invariance) for non-invariant intercepts of item 5.

	Intercepts fixed		Partial invariance	
Year	β (95% CI)	p	β (95% CI)	p
2010	0.255 (0.191, 0.319)	<0.001	0.239 (0.174, 0.303)	<0.001
2011	-0.022 (-0.110, 0.067)	0.634	-0.026 (-0.116, 0.063)	0.567
2012	0.142 (0.088, 0.196)	<0.001	0.136 (0.081, 0.190)	<0.001
2013	0.083 (0.04, 0.126)	<0.001	0.070 (0.027, 0.114)	0.002
**2014**	**ref.**		**ref.**	
2015	-0.018 (-0.062, 0.026)	0.412	-0.018 (-0.062, 0.027)	0.437
2016	-0.018 (-0.062, 0.026)	0.424	-0.004 (-0.049, 0.040)	0.846
2017	0.154 (0.113, 0.195)	<0.001	0.158 (0.116, 0.199)	<0.001
2018	0.184 (0.139, 0.228)	<0.001	0.196 (0.151, 0.241)	<0.001
2019	0.181 (0.141, 0.221)	<0.001	0.194 (0.153, 0.234)	<0.001

Note. Ref. = reference group (survey year 2014). β = standardized regression coefficient. 95% CI =95 % confidence intervals of β .

Supplementary Table 12. Intercept means and d_{MACS} effect size of invariance, partial invariance model boys 15 year olds.

Year	Intercept mean (95% CI)	d_{MACS}
2010	ref.	ref.
2011	0.099 (0.050, 0.147)	0.028
2012	0.074 (0.002, 0.146)	0.020
2013	0.073 (0.030, 0.117)	0.023
2014	0.079 (0.043, 0.116)	0.072
2015	-0.026 (-0.067, 0.014)	0.101
2016	-0.103 (-0.145, -0.061)	0.175
2017	-0.063 (-0.101, -0.024)	0.138
2018	-0.095 (-0.137, -0.053)	0.171
2019	-0.122 (-0.161, -0.084)	0.201

Supplementary Table 13. Trends in latent mean scores among 15 year old boys. Comparing models not accounting (Intercepts fixed) and accounting (Partial invariance) for non-invariant intercepts of item 5

	Intercepts fixed		Partial invariance	
**Year**	β (95% CI)	p	β (95% CI)	p
2010	0.255 (0.193, 0.317)	<0.001	0.239 (0.177, 0.302)	<0.001
2011	-0.028 (-0.108, 0.052)	0.492	-0.038 (-0.119, 0.042)	0.351
2012	0.150 (0.098, 0.202)	<0.001	0.138 (0.086, 0.191)	<0.001
2013	0.043 (0.002, 0.084)	0.038	0.031 (-0.01, 0.073)	0.136
**2014**	**ref.**		**ref.**	
2015	0.021 (-0.022, 0.064)	0.332	0.025 (-0.018, 0.068)	0.258
2016	-0.051 (-0.093, -0.009)	0.018	-0.035 (-0.078, 0.007)	0.104
2017	0.120 (0.081, 0.160)	<0.001	0.129 (0.090, 0.169)	<0.001
2018	0.147 (0.104, 0.190)	<0.001	0.161 (0.118, 0.204)	<0.001
2019	0.172 (0.133, 0.211)	<0.001	0.190 (0.151, 0.229)	<0.001

Note. Ref. = reference group (survey year 2014). β = standardized regression coefficient. 95% CI =95 % confidence intervals of β .

Supplementary Table 14. Intercept means and d_{MACS} effect size of invariance, partial invariance model boys 16 year olds.

Year	Intercept mean (95% CI)	d_{MACS}
2010	ref.	ref.
2011	0.159 (0.092, 0.227)	0.076
2012	0.218 (0.107, 0.328)	0.026
2013	0.144 (0.094, 0.193)	0.017
2014	0.162 (0.119, 0.205)	0.131
2015	-0.016 (-0.062, 0.030)	0.160
2016	-0.022 (-0.068, 0.024)	0.162
2017	0.004 (-0.040, 0.048)	0.142
2018	-0.026 (-0.073, 0.020)	0.167
2019	-0.025 (-0.068, 0.018)	0.174

Supplementary Table 15. Trends in latent mean scores among 16 year old boys. Comparing models not accounting (Intercepts fixed) and accounting (Partial invariance) for non-invariant intercepts of item 5

	Intercepts fixed		Partial invariance	
Year	β (95% CI)	p	β (95% CI)	p
2010	0.232 (0.143, 0.321)	<0.001	0.206 (0.117, 0.295)	<0.001
2011	0.069 (-0.061, 0.198)	0.298	0.039 (-0.092, 0.169)	0.560
2012	0.208 (0.145, 0.271)	<0.001	0.185 (0.122, 0.249)	<0.001
2013	0.108 (0.059, 0.157)	<0.001	0.083 (0.034, 0.133)	0.001
**2014**	**ref.**		**ref.**	
2015	0.036 (-0.012, 0.084)	0.142	0.039 (-0.01, 0.087)	0.117
2016	0.034 (-0.014, 0.082)	0.162	0.038 (-0.011, 0.086)	0.125
2017	0.128 (0.082, 0.173)	<0.001	0.128 (0.082, 0.173)	<0.001
2018	0.183 (0.135, 0.231)	<0.001	0.187 (0.139, 0.236)	<0.001
2019	0.192 (0.148, 0.235)	<0.001	0.196 (0.152, 0.239)	<0.001

Note. Ref. = reference group (survey year 2014). β = standardized regression coefficient. 95% CI =95 % confidence intervals of β .

Measurement invariance across age groups

Supplementary Table 16. Measurement invariance across age groups among boys and girls, pooled sample (2010 – 2019)

Model	χ2 (df)	p	CFI	RMSEA (90 %CI)	TLI	SRMR	ΔCFI	ΔRMSEA
Girls								
Baseline	12230.964 (54)	<.001	0.994	0.069 (0.068, 0.07)	0.990	0.022		
Baseline*	6509.174 (48)	<.001	0.997	0.053 (0.052, 0.054)	0.994	0.016		
Thresholds	7467.509 (78)	<.001	0.996	0.045 (0.044, 0.046)	0.996	0.016	-0.0004	-0.0086
Thresholds and loadings	6305.492 (103)	<.001	0.997	0.036 (0.035, 0.036)	0.997	0.017	0.0006	-0.0091
Thresholds, loadings, intercepts	17072.938 (128)	<.001	0.992	0.053 (0.052, 0.053)	0.994	0.017	-0.0052	0.0172
Partial invariance; free intercept item 3 & 2	8420.560 (118)	<.001	0.996	0.038 (0.038, 0.039)	0.997	0.017	-0.0010	0.0029
Boys								
Baseline	10594.592 (54)	<.001	0.993	0.065 (0.064, 0.066)	0.989	0.024		
Baseline*	6359.176 (48)	<.001	0.996	0.054 (0.052, 0.055)	0.992	0.018		
Thresholds	6742.982 (78)	<.001	0.996	0.043 (0.042, 0.044)	0.995	0.018	-0.0002	-0.0104
Thresholds and loadings	5298.761 (103)	<.001	0.997	0.033 (0.032, 0.034)	0.997	0.018	0.0009	-0.0100
Thresholds, loadings, intercepts	6884.378 (128)	<.001	0.996	0.034 (0.033, 0.035)	0.997	0.019	-0.0010	0.0008

Note. χ^2= chi-square goodness-of-fit based on the Satorra-Bentler correction; df = degrees of freedom; CFI; comparative fit index; RMSEA = root mean square error of approximation; CI = confidence interval. TLI = Tucker Lewis Index. SRMR = standardized root mean squared residual; ΔCFI/RMSEA = Change in CFI/RMSEA between models.
* Modelling correlated error terms between item 3 and 4.

Supplementary Table 17. Measurement invariance across age groups by survey years; Girls

**Model**	χ2 (df)	p	CFI	RMSEA (90 %CI)	TLI	SRMR	ΔCFI	ΔRMSEA
2010								
Baseline*	232.051 (40)	<.001	0.996	0.057 (0.050, 0.065)	0.992	0.020		
Thresholds	279.118 (64)	<.001	0.995	0.048 (0.042, 0.054)	0.994	0.020	-0.0005	-0.0094
Thresholds and loadings	247.828 (84)	<.001	0.996	0.037 (0.031, 0.042)	0.997	0.020	0.0011	-0.0114
Thresholds, loadings, intercepts	459.498 (104)	<.001	0.992	0.048 (0.044, 0.053)	0.994	0.021	-0.0042	0.0118
Partial scalar: free intercepts item 3 and 2	335.352 (96)	<.001	0.995	0.041 (0.037, 0.046)	0.996	0.020	-0.0017	0.0048
2011								
Baseline†	80.836 (40)	0.557	0.998	0.040 (0.027, 0.052)	0.996	0.017		
Thresholds	112.794 (64)	0.870	0.998	0.035 (0.024, 0.045)	0.997	0.017	-0.0004	-0.0054
Thresholds and loadings	144.945 (84)	0.571	0.997	0.034 (0.024, 0.043)	0.997	0.019	-0.0006	-0.0008
Thresholds, loadings, intercepts	252.708 (104)	<.001	0.993	0.047 (0.040, 0.055)	0.995	0.019	-0.0043	0.0136
Partial scalar: free intercept item 1 and 5	185.642 (96)	0.115	0.996	0.038 (0.030, 0.046)	0.997	0.019	-0.0014	0.0045
2012								
Baseline†	393.783 (40)	<.001	0.996	0.062 (0.056, 0.068)	0.992	0.019		
Thresholds	458.731 (64)	<.001	0.995	0.052 (0.047, 0.056)	0.994	0.019	-0.0005	-0.0102
Thresholds and loadings	409.026 (84)	<.001	0.996	0.041 (0.037, 0.045)	0.996	0.020	0.0009	-0.0107
Thresholds, loadings, intercepts	650.491 (104)	<.001	0.993	0.048 (0.044, 0.051)	0.995	0.020	-0.0028	0.0068
Partial scalar: free intercept item 3	537.776 (100)	<.001	0.994	0.044 (0.040, 0.047)	0.996	0.020	-0.0014	0.0026
2013								
Baseline†	1150.972 (40)	<.001	0.996	0.059 (0.056, 0.062)	0.993	0.018		
Thresholds	1262.832 (64)	<.001	0.996	0.049 (0.046, 0.051)	0.995	0.018	-0.0003	-0.0106
Thresholds and loadings	1092.172 (84)	<.001	0.996	0.039 (0.037, 0.041)	0.997	0.018	0.0007	-0.0097
Thresholds, loadings, intercepts	2489.964 (104)	<.001	0.992	0.054 (0.052, 0.056)	0.994	0.019	-0.0049	0.0149
Partial scalar: free intercept item 3 and 2	1409.653 (96)	<.001	0.995	0.042 (0.040, 0.044)	0.996	0.018	-0.0011	0.0026
2014								
Baseline*	655.657 (40)	<.001	0.996	0.06 (0.056, 0.064)	0.992	0.02		
Thresholds	732.415 (64)	<.001	0.995	0.05 (0.046, 0.053)	0.995	0.02	-0.0004	-0.0106
Thresholds and loadings	615.076 (84)	<.001	0.996	0.039 (0.036, 0.041)	0.997	0.02	0.001	-0.011
Thresholds, loadings, intercepts	1373.661 (104)	<.001	0.991	0.054 (0.051, 0.056)	0.994	0.021	-0.0051	0.015
Partial scalar: free intercept item 3 and 2	765.782 (96)	<.001	0.995	0.04 (0.038, 0.043)	0.996	0.02	-0.001	0.0019
2015								

Baseline*	713.838 (40)	<.001	0.997	0.05 (0.047, 0.053)	0.995	0.016		
Thresholds	824.236 (64)	<.001	0.997	0.042 (0.04, 0.045)	0.996	0.016	-0.0003	-0.008
Thresholds and loadings	720.842 (84)	<.001	0.997	0.034 (0.031, 0.036)	0.998	0.016	0.0005	-0.0084
Thresholds, loadings, intercepts	1842.777 (104)	<.001	0.993	0.05 (0.048, 0.052)	0.995	0.017	-0.0045	0.0163
Partial scalar: free intercept item 3 and 2	1038.953 (96)	<.001	0.996	0.038 (0.036, 0.04)	0.997	0.016	-0.0012	0.0046
2016								
Baseline*	660.711 (40)	<.001	0.997	0.049 (0.046, 0.052)	0.995	0.016		
Thresholds	856.681 (64)	<.001	0.997	0.044 (0.041, 0.047)	0.996	0.016	-0.0007	-0.0052
Thresholds and loadings	797.442 (84)	<.001	0.997	0.036 (0.034, 0.039)	0.997	0.016	0.0003	-0.0076
Thresholds, loadings, intercepts	2130.06 (104)	<.001	0.991	0.055 (0.053, 0.057)	0.994	0.017	-0.0056	0.0187
Partial scalar: free intercept item 3 and 2	1154.294 (96)	<.001	0.995	0.041 (0.039, 0.044)	0.996	0.016	-0.0015	0.0051
2017								
Baseline*	1014.386 (40)	<.001	0.997	0.051 (0.048, 0.053)	0.995	0.016		
Thresholds	1218.089 (64)	<.001	0.997	0.044 (0.041, 0.046)	0.996	0.016	-0.0005	-0.0071
Thresholds and loadings	1092.568 (84)	<.001	0.997	0.036 (0.034, 0.037)	0.997	0.016	0.0004	-0.008
Thresholds, loadings, intercepts	2845.766 (104)	<.001	0.992	0.053 (0.051, 0.054)	0.994	0.017	-0.005	0.0171
Partial scalar: free intercept item 3 and 2	1559.612 (96)	<.001	0.996	0.04 (0.038, 0.042)	0.997	0.016	-0.0013	0.0045
2018								
Baseline*	787.842 (40)	<.001	0.997	0.053 (0.05, 0.056)	0.994	0.017		
Thresholds	897.443 (64)	<.001	0.997	0.044 (0.041, 0.047)	0.996	0.017	-0.0004	-0.0087
Thresholds and loadings	782.701 (84)	<.001	0.997	0.035 (0.033, 0.037)	0.997	0.017	0.0006	-0.0088
Thresholds, loadings, intercepts	2079.341 (104)	<.001	0.992	0.053 (0.051, 0.055)	0.994	0.018	-0.0053	0.018
Partial scalar: free intercept item 3 and 2	1138.084 (96)	<.001	0.996	0.04 (0.038, 0.042)	0.997	0.017	-0.0014	0.005
2019								
Baseline	1238.06 (40)	<.001	0.997	0.052 (0.049, 0.054)	0.995	0.016		
Thresholds	1421.849 (64)	<.001	0.997	0.044 (0.042, 0.046)	0.996	0.016	-0.0004	-0.0082
Thresholds and loadings	1198.997 (84)	<.001	0.997	0.035 (0.033, 0.036)	0.998	0.016	0.0006	-0.0091
Thresholds, loadings, intercepts	3742.465 (104)	<.001	0.991	0.056 (0.055, 0.058)	0.994	0.017	-0.0061	0.0216
Partial scalar: free intercept item 3 and 2	1703.964 (96)	<.001	0.996	0.039 (0.037, 0.040)	0.997	0.016	-0.0012	0.0043

Note. χ^2= chi-square goodness-of-fit based on the Satorra-Bentler correction; df = degrees of freedom; CFI; comparative fit index; RMSEA = root mean square error of approximation; CI = confidence interval. TLI = Tucker Lewis Index. SRMR = standardized root mean squared residual; ΔCFI/RMSEA = Change in CFI/RMSEA between models.
† Models accounting for one correlated error term between item 3 and 5; * models accounting for one correlated error term between item 3 and 4

Supplementary Table 18. Measurement invariance across age groups by survey years; Boys

**Model**	χ2 (df)	p	CFI	RMSEA (90 %CI)	TLI	SRMR	ΔCFI	ΔRMSEA
2010								
Baseline (2-5)*	267.508 (40)	<.001	0.994	0.064 (0.057, 0.072)	0.989	0.022		
Thresholds	313.314 (64)	<.001	0.994	0.053 (0.047, 0.059)	0.993	0.022	-0.0006	-0.0111
Thresholds and loadings	284.978 (84)	<.001	0.995	0.042 (0.036, 0.047)	0.995	0.023	0.0012	-0.0115
Thresholds, loadings, intercepts	356.777 (104)	<.001	0.994	0.042 (0.037, 0.047)	0.995	0.023	-0.0013	0.0003
2011								
Baseline (3-5)*	127.341 (40)	0.022	0.995	0.058 (0.047, 0.069)	0.99	0.026		
Thresholds	142.981 (64)	0.405	0.995	0.044 (0.034, 0.053)	0.995	0.026	0.0005	-0.0144
Thresholds and loadings	132.652 (84)	0.742	0.997	0.03 (0.02, 0.039)	0.997	0.026	0.0018	-0.0137
Thresholds, loadings, intercepts	153.906 (104)	0.667	0.997	0.027 (0.017, 0.036)	0.998	0.027	-0.0001	-0.0027
2012								
Baseline (3-4, 2-6)*	318.004 (35)	<.001	0.995	0.061 (0.055, 0.067)	0.99	0.02		
Thresholds	342.596 (59)	<.001	0.995	0.047 (0.042, 0.052)	0.994	0.02	0	-0.0139
Thresholds and loadings	312.239 (79)	<.001	0.996	0.037 (0.032, 0.041)	0.996	0.02	0.0008	-0.0101
Thresholds, loadings, intercepts	450.308 (99)	<.001	0.994	0.04 (0.036, 0.044)	0.996	0.021	-0.0019	0.0035
2013								
Baseline (3-4)*	1132.557 (40)	<.001	0.994	0.06 (0.057, 0.063)	0.989	0.021		
Thresholds	1198.509 (64)	<.001	0.994	0.048 (0.046, 0.051)	0.993	0.021	-0.0002	-0.0116
Thresholds and loadings	954.101 (84)	<.001	0.995	0.037 (0.035, 0.039)	0.996	0.022	0.0014	-0.0114
Thresholds, loadings, intercepts	1148.746 (104)	<.001	0.995	0.036 (0.034, 0.038)	0.996	0.022	-0.0009	-0.0006
2014								
Baseline (3-4)*	452.237 (40)	<.001	0.996	0.05 (0.046, 0.054)	0.992	0.019		
Thresholds	486.858 (64)	<.001	0.996	0.04 (0.036, 0.043)	0.995	0.019	-0.0001	-0.0099
Thresholds and loadings	420.656 (84)	<.001	0.997	0.031 (0.028, 0.034)	0.997	0.019	0.0008	-0.0088
Thresholds, loadings, intercepts	540.967 (104)	<.001	0.996	0.032 (0.029, 0.034)	0.997	0.02	-0.001	0.0007
2015								
Baseline (3-4)*	723.191 (40)	<.001	0.996	0.052 (0.048, 0.055)	0.993	0.018		
Thresholds	1000.497 (64)	<.001	0.995	0.048 (0.045, 0.051)	0.994	0.021	-0.0015	-0.0039
Thresholds and loadings	826.076 (84)	<.001	0.996	0.037 (0.035, 0.04)	0.996	0.021	0.0011	-0.0107
Thresholds, loadings, intercepts	1034.581 (104)	<.001	0.995	0.037 (0.035, 0.04)	0.996	0.022	-0.0011	0.0002
2016								
Baseline (3-4)*	748.261 (40)	<.001	0.995	0.053 (0.05, 0.057)	0.991	0.02		
Thresholds	826.359 (64)	<.001	0.995	0.044 (0.041, 0.046)	0.994	0.02	-0.0004	-0.0096
Thresholds and loadings	674.891 (84)	<.001	0.996	0.034 (0.031, 0.036)	0.997	0.02	0.0011	-0.0101
Thresholds, loadings, intercepts	984.017 (104)	<.001	0.994	0.037 (0.035, 0.039)	0.996	0.021	-0.0019	0.0032

2017								
Baseline (3-4)*	962.959 (40)	<.001	0.997	0.05 (0.047, 0.052)	0.994	0.018		
Thresholds	1079.825 (64)	<.001	0.996	0.041 (0.039, 0.043)	0.996	0.018	-0.0003	-0.0085
Thresholds and loadings	890.175 (84)	<.001	0.997	0.032 (0.03, 0.034)	0.997	0.018	0.0008	-0.0092
Thresholds, loadings, intercepts	1138.389 (104)	<.001	0.996	0.033 (0.031, 0.034)	0.997	0.018	-0.0009	0.0006
2018								
Baseline (3-4)*	720.609 (40)	<.001	0.996	0.052 (0.049, 0.055)	0.993	0.018		
Thresholds	775.192 (64)	<.001	0.996	0.042 (0.039, 0.045)	0.996	0.018	-0.0002	-0.0099
Thresholds and loadings	641.092 (84)	<.001	0.997	0.032 (0.03, 0.035)	0.997	0.018	0.0008	-0.0095
Thresholds, loadings, intercepts	735.562 (104)	<.001	0.997	0.031 (0.029, 0.033)	0.998	0.018	-0.0004	-0.0014
2019								
Baseline (3-4)*	1180.672 (40)	<.001	0.997	0.052 (0.049, 0.054)	0.994	0.017		
Thresholds	1246.034 (64)	<.001	0.996	0.042 (0.04, 0.044)	0.996	0.017	-0.0001	-0.0101
Thresholds and loadings	1027.947 (84)	<.001	0.997	0.032 (0.031, 0.034)	0.998	0.017	0.0007	-0.0091
Thresholds, loadings, intercepts	1371.809 (104)	<.001	0.996	0.034 (0.032, 0.035)	0.997	0.018	-0.001	0.0014

Note. χ^2= chi-square goodness-of-fit based on the Satorra-Bentler correction; df = degrees of freedom; CFI; comparative fit index; RMSEA = root mean square error of approximation; CI = confidence interval. TLI = Tucker Lewis Index. SRMR = standardized root mean squared residual; ΔCFI/RMSEA = Change in CFI/RMSEA between models.
*Correlated error terms accounted for.

Supplementary Table 19. Intercept means and d_{MACS} effect size of noninvariant item 2 and 3, partial invariance model girls pooled sample.

Items	Age	Intercept mean (95% CI)	d_{MACS}
Item 2	13-year-olds	**ref.**	**ref.**
Item 2	14-year-olds	-0.11 (-0.12,-0.099)	0.109
Item 2	15-year-olds	-0.163 (-0.174,-0.153)	0.166
Item 2	16-year-olds	-0.175 (-0.186,-0.163)	0.176
Item 2	17-year-olds	-0.22 (-0.232,-0.208)	0.226
Item 2	18-year-olds	-0.262 (-0.276,-0.248)	0.273
Item 3	13-year-olds	**ref.**	**ref.**
Item 3	14-year-olds	-0.104 (-0.113,-0.095)	0.106
Item 3	15-year-olds	-0.2 (-0.209,-0.191)	0.209
Item 3	16-year-olds	-0.199 (-0.208,-0.19)	0.215
Item 3	17-year-olds	-0.274 (-0.284,-0.263)	0.297
Item 3	18-year-olds	-0.393 (-0.404,-0.381)	0.433

Supplementary Table 20. Comparing latent means across age groups in Girls (pooled sample). Comparing models not accounting (Intercepts fixed) and accounting for (partial invariance) non-invariant intercepts of items 2 and 3.

	Intercepts fixed		Partial invariance	
**Age**	β (95% CI)	p	β (95% CI)	p
13	ref.		ref.	
14	0.380 (0.367, 0.393)	<0.001	0.418 (0.404, 0.431)	<0.001
15	0.609 (0.595, 0.623)	<0.001	0.677 (0.663, 0.691)	<0.001
16	0.673 (0.658, 0.687)	<0.001	0.743 (0.728, 0.758)	<0.001
17	0.708 (0.691, 0.724)	<0.001	0.803 (0.786, 0.820)	<0.001
18	0.837 (0.817, 0.856)	<0.001	0.973 (0.952, 0.994)	<0.001

Note. Ref. = reference group (age group = 13). β = standardized regression coefficient. 95% CI =95 % confidence intervals of β .

Supplementary Table 21. Intercept means and d_{MACS} effect size of noninvariant item 3, partial invariance model girls 2010.

Items	Age	Intercept mean (95% CI)	d_{MACS}
Item 2	13-year-olds	**ref.**	**ref.**
Item 2	14-year-olds	-0.094 (-0.154,-0.035)	0.098
Item 2	15-year-olds	-0.151 (-0.211,-0.09)	0.155
Item 2	16-year-olds	-0.166 (-0.243,-0.089)	0.169
Item 2	17-18-year-olds	-0.184 (-0.28,-0.088)	0.209
Item 3	13-year-olds	**ref.**	**ref.**
Item 3	14-year-olds	-0.108 (-0.158,-0.058)	0.119
Item 3	15-year-olds	-0.210 (-0.262,-0.159)	0.226
Item 3	16-year-olds	-0.177 (-0.237,-0.117)	0.201
Item 3	17-18-year-olds	-0.309 (-0.395,-0.223)	0.349

Supplementary Table 22. Intercept means and d_{MACS} effect size of noninvariant item 3, partial invariance model girls 2011.

Items	Age	Intercept mean (95% CI)	d_{MACS}
Item 1	13-year-olds	**ref.**	**ref.**
Item 1	14-year-olds	0.217 (0.135,0.298)	0.211
Item 1	15-year-olds	0.208 (0.128,0.287)	0.209
Item 1	16-year-olds	0.188 (0.088,0.288)	0.192
Item 1	17-18-year-olds	0.238 (0.132,0.344)	0.242
Item 5	13-year-olds	**ref.**	**ref.**
Item 5	14-year-olds	-0.032 (-0.123,0.059)	0.027
Item 5	15-year-olds	0.010 (-0.077,0.098)	0.022
Item 5	16-year-olds	0.173 (0.069,0.278)	0.184
Item 5	17-18-year-olds	0.164 (0.033,0.294)	0.178

Supplementary Table 23. Intercept means and d_{MACS} effect size of noninvariant item 3, partial invariance model girls 2012.

Items	Age	Intercept mean (95% CI)	d_{MACS}
Item 3	13-year-olds	**ref.**	**ref.**
Item 3	14-year-olds	-0.115 (-0.162,-0.068)	0.113
Item 3	15-year-olds	-0.208 (-0.255,-0.160)	0.211
Item 3	16-year-olds	-0.220 (-0.271,-0.170)	0.235
Item 3	17-18-year-olds	-0.196 (-0.259,-0.133)	0.213

Supplementary Table 24. Intercept means and d_{MACS} effect size of noninvariant item 2 and 3, partial invariance model girls 2013.

Items	Age	Intercept mean (95% CI)	d_{MACS}
Item 2	13-year-olds	**ref.**	**ref.**
Item 2	14-year-olds	-0.086 (-0.113,-0.058)	0.087
Item 2	15-year-olds	-0.13 (-0.157,-0.104)	0.137
Item 2	16-year-olds	-0.15 (-0.179,-0.122)	0.158
Item 2	17-18-year-olds	-0.233 (-0.266,-0.2)	0.248
Item 3	13-year-olds	**ref.**	**ref.**
Item 3	14-year-olds	-0.118 (-0.141,-0.096)	0.123
Item 3	15-year-olds	-0.215 (-0.238,-0.192)	0.229
Item 3	16-year-olds	-0.232 (-0.256,-0.208)	0.256
Item 3	17-18-year-olds	-0.354 (-0.382,-0.327)	0.398

Supplementary Table 25. Intercept means and d_{MACS} effect size of noninvariant item 2 and 3, partial invariance model girls 2014.

Items	Age	Intercept mean (95% CI)	d_{MACS}
Item 2	13-year-olds	**ref.**	**ref.**
Item 2	14-year-olds	-0.098 (-0.136,-0.06)	0.103
Item 2	15-year-olds	-0.149 (-0.188,-0.111)	0.155
Item 2	16-year-olds	-0.199 (-0.244,-0.154)	0.197
Item 2	17-18-year-olds	-0.25 (-0.292,-0.208)	0.255
Item 3	13-year-olds	**ref.**	**ref.**
Item 3	14-year-olds	-0.097 (-0.129,-0.065)	0,099
Item 3	15-year-olds	-0.205 (-0.238,-0.172)	0,216
Item 3	16-year-olds	-0.200 (-0.236,-0.165)	0,216
Item 3	17-18-year-olds	-0.324 (-0.358,-0.289)	0,354

Supplementary Table 26. Intercept means and d_{MACS} effect size of noninvariant item 2 and 3, partial invariance model girls 2015.

Items	Age	Intercept mean (95% CI)	d_{MACS}
Item 2	13-year-olds	**ref.**	**ref.**
Item 2	14-year-olds	-0.105 (-0.137,-0.073)	0.109
Item 2	15-year-olds	-0.167 (-0.2,-0.135)	0.176
Item 2	16-year-olds	-0.15 (-0.184,-0.117)	0.157
Item 2	17-18-year-olds	-0.205 (-0.237,-0.173)	0.216
Item 3	13-year-olds	**ref.**	**ref.**
Item 3	14-year-olds	-0.092 (-0.118,-0.065)	0.097
Item 3	15-year-olds	-0.191 (-0.218,-0.164)	0.205
Item 3	16-year-olds	-0.162 (-0.189,-0.135)	0.183
Item 3	17-18-year-olds	-0.295 (-0.322,-0.268)	0.336

Supplementary Table 27. Intercept means and d_{MACS} effect size of noninvariant item 2 and 3, partial invariance model girls 2016.

Items	Age	Intercept mean (95% CI)	d_{MACS}
Item 2	13-year-olds	**ref.**	**ref.**
Item 2	14-year-olds	-0.133 (-0.165,-0.1)	0.132
Item 2	15-year-olds	-0.193 (-0.224,-0.161)	0.199
Item 2	16-year-olds	-0.226 (-0.259,-0.194)	0.234
Item 2	17-18-year-olds	-0.284 (-0.318,-0.249)	0.300
Item 3	13-year-olds	**ref.**	**ref.**
Item 3	14-year-olds	-0.138 (-0.165,-0.112)	0.144
Item 3	15-year-olds	-0.232 (-0.259,-0.205)	0.248
Item 3	16-year-olds	-0.228 (-0.254,-0.202)	0.251
Item 3	17-18-year-olds	-0.314 (-0.342,-0.285)	0.352

Supplementary Table 28. Intercept means and d_{MACS} effect size of noninvariant item 2 and 3, partial invariance model girls 2017.

Items	Age	Intercept mean (95% CI)	d_{MACS}
Item 2	13-year-olds	**ref.**	**ref.**
Item 2	14-year-olds	-0.125 (-0.152,-0.098)	0.122
Item 2	15-year-olds	-0.184 (-0.211,-0.157)	0.18
Item 2	16-year-olds	-0.178 (-0.206,-0.15)	0.177
Item 2	17-18-year-olds	-0.231 (-0.258,-0.204)	0.232
Item 3	13-year-olds	**ref.**	**ref.**
Item 3	14-year-olds	-0.113 (-0.136,-0.091)	0.113
Item 3	15-year-olds	-0.203 (-0.226,-0.18)	0.207
Item 3	16-year-olds	-0.186 (-0.209,-0.164)	0.198
Item 3	17-18-year-olds	-0.309 (-0.331,-0.287)	0.335

Supplementary Table 29. Intercept means and d_{MACS} effect size of noninvariant item 2 and 3, partial invariance model girls 2018.

**Items**	**Age**	Intercept mean (95% CI)	d_{MACS}
Item 2	13-year-olds	**ref.**	**ref.**
Item 2	14-year-olds	-0.111 (-0.143,-0.078)	0.109
Item 2	15-year-olds	-0.176 (-0.209,-0.143)	0.174
Item 2	16-year-olds	-0.188 (-0.222,-0.154)	0.185
Item 2	17-18-year-olds	-0.238 (-0.269,-0.207)	0.241
Item 3	13-year-olds	**ref.**	**ref.**
Item 3	14-year-olds	-0.065 (-0.092,-0.038)	0.066
Item 3	15-year-olds	-0.167 (-0.195,-0.139)	0.172

Item 3	16-year-olds	-0.169 (-0.196,-0.141)	0.178
Item 3	17-18-year-olds	-0.280 (-0.306,-0.254)	0.303

Supplementary Table 30. Intercept means and d_{MACS} effect size of noninvariant item 2 and 3, partial invariance model girls 2019.

Items	Age	Intercept mean (95% CI)	d_{MACS}
Item 2	13-year-olds	**ref.**	
Item 2	14-year-olds	-0.132 (-0.158,-0.106)	0.130
Item 2	15-year-olds	-0.181 (-0.206,-0.155)	0.181
Item 2	16-year-olds	-0.175 (-0.2,-0.15)	0.175
Item 2	17-18-year-olds	-0.234 (-0.257,-0.21)	0.235
Item 3	13-year-olds	**ref.**	
Item 3	14-year-olds	-0.118 (-0.139,-0.098)	0.122
Item 3	15-year-olds	-0.230 (-0.251,-0.208)	0.235
Item 3	16-year-olds	-0.229 (-0.25,-0.209)	0.241
Item 3	17-18-year-olds	-0.350 (-0.370,-0.330)	0.370

Supplementary Table 31. Latent means across age groups in Girls per survey year. Comparing models not accounting (Intercepts fixed) and accounting for (partial invariance) non-invariant parameters

		Intercepts fixed		Partial invariance	
Year	Age	β (95% CI)	p	β (95% CI)	p
2010	13	**ref.**		**ref.**	
	14	0.460 (0.387, 0.534)	<0.001	0.498 (0.423, 0.574)	<0.001
	15	0.648 (0.574, 0.723)	<0.001	0.718 (0.641, 0.795)	<0.001
	16	0.803 (0.703, 0.902)	<0.001	0.869 (0.766, 0.971)	<0.001
	17-18	0.810 (0.664, 0.955)	<0.001	0.911 (0.759, 1.062)	<0.001
2011	13	**ref.**		**ref.**	
	14	0.302 (0.198, 0.407)	<0.001	0.252 (0.146, 0.359)	<0.001
	15	0.553 (0.443, 0.662)	<0.001	0.495 (0.384, 0.605)	<0.001
	16	0.592 (0.446, 0.737)	<0.001	0.506 (0.359, 0.653)	<0.001
	17-18	0.511 (0.343, 0.679)	<0.001	0.408 (0.238, 0.578)	<0.001
2012	13	**ref.**		**ref.**	
	14	0.385 (0.321, 0.449)	<0.001	0.409 (0.344, 0.474)	<0.001
	15	0.659 (0.593, 0.726)	<0.001	0.705 (0.637, 0.773)	<0.001
	16	0.769 (0.694, 0.843)	<0.001	0.818 (0.741, 0.894)	<0.001
	17-18	0.660 (0.56, 0.761)	<0.001	0.703 (0.6, 0.806)	<0.001
2013	13	**ref.**		**ref.**	
	14	0.425 (0.392, 0.458)	<0.001	0.464 (0.43, 0.498)	<0.001
	15	0.649 (0.615, 0.682)	<0.001	0.719 (0.685, 0.754)	<0.001
	16	0.705 (0.667, 0.742)	<0.001	0.786 (0.746, 0.825)	<0.001
	17-18	0.717 (0.674, 0.76)	<0.001	0.845 (0.799, 0.89)	<0.001
2014	13	**ref.**		**ref.**	
	14	0.356 (0.312, 0.401)	<0.001	0.39 (0.344, 0.436)	<0.001
	15	0.617 (0.569, 0.665)	<0.001	0.685 (0.635, 0.734)	<0.001
	16	0.634 (0.58, 0.689)	<0.001	0.708 (0.653, 0.764)	<0.001
	17-18	0.65 (0.599, 0.701)	<0.001	0.763 (0.71, 0.815)	<0.001
2015	13	**ref.**		**ref.**	

	14	0.407 (0.369, 0.445)	<0.001	0.439 (0.4, 0.478)	<0.001
	15	0.688 (0.647, 0.729)	<0.001	0.751 (0.709, 0.793)	<0.001
	16	0.732 (0.689, 0.775)	<0.001	0.788 (0.744, 0.832)	<0.001
	17-18	0.79 (0.748, 0.831)	<0.001	0.887 (0.845, 0.93)	<0.001
2016	13	**ref.**		**ref.**	
	14	0.397 (0.358, 0.436)	<0.001	0.445 (0.405, 0.485)	<0.001
	15	0.704 (0.663, 0.746)	<0.001	0.783 (0.741, 0.826)	<0.001
	16	0.759 (0.717, 0.801)	<0.001	0.843 (0.8, 0.886)	<0.001
	17-18	0.863 (0.816, 0.911)	<0.001	0.981 (0.932, 1.03)	<0.001
2017	13	**ref.**		**ref.**	
	14	0.424 (0.392, 0.456)	<0.001	0.464 (0.431, 0.496)	<0.001
	15	0.62 (0.587, 0.654)	<0.001	0.689 (0.655, 0.724)	<0.001
	16	0.682 (0.647, 0.717)	<0.001	0.748 (0.712, 0.784)	<0.001
	17-18	0.733 (0.699, 0.767)	<0.001	0.838 (0.803, 0.873)	<0.001
2018	13	**ref.**		**ref.**	
	14	0.353 (0.314, 0.392)	<0.001	0.382 (0.343, 0.422)	<0.001
	15	0.571 (0.53, 0.613)	<0.001	0.634 (0.591, 0.676)	<0.001
	16	0.595 (0.553, 0.636)	<0.001	0.657 (0.615, 0.7)	<0.001
	17-18	0.708 (0.668, 0.747)	<0.001	0.809 (0.769, 0.849)	<0.001
2019	13	**ref.**		**ref.**	
	14	0.302 (0.271, 0.334)	<0.001	0.348 (0.316, 0.38)	<0.001
	15	0.465 (0.433, 0.497)	<0.001	0.544 (0.511, 0.576)	<0.001
	16	0.521 (0.489, 0.553)	<0.001	0.6 (0.567, 0.632)	<0.001
	17-18	0.631 (0.601, 0.662)	<0.001	0.751 (0.72, 0.783)	<0.001

Note. Ref. = reference group (age group = 13). β = standardized regression coefficient. 95% CI =95 % confidence intervals of β .

Supplementary Figure 6. The practical consequences of noninvariance on latent mean differences across age groups in girls, by survey year

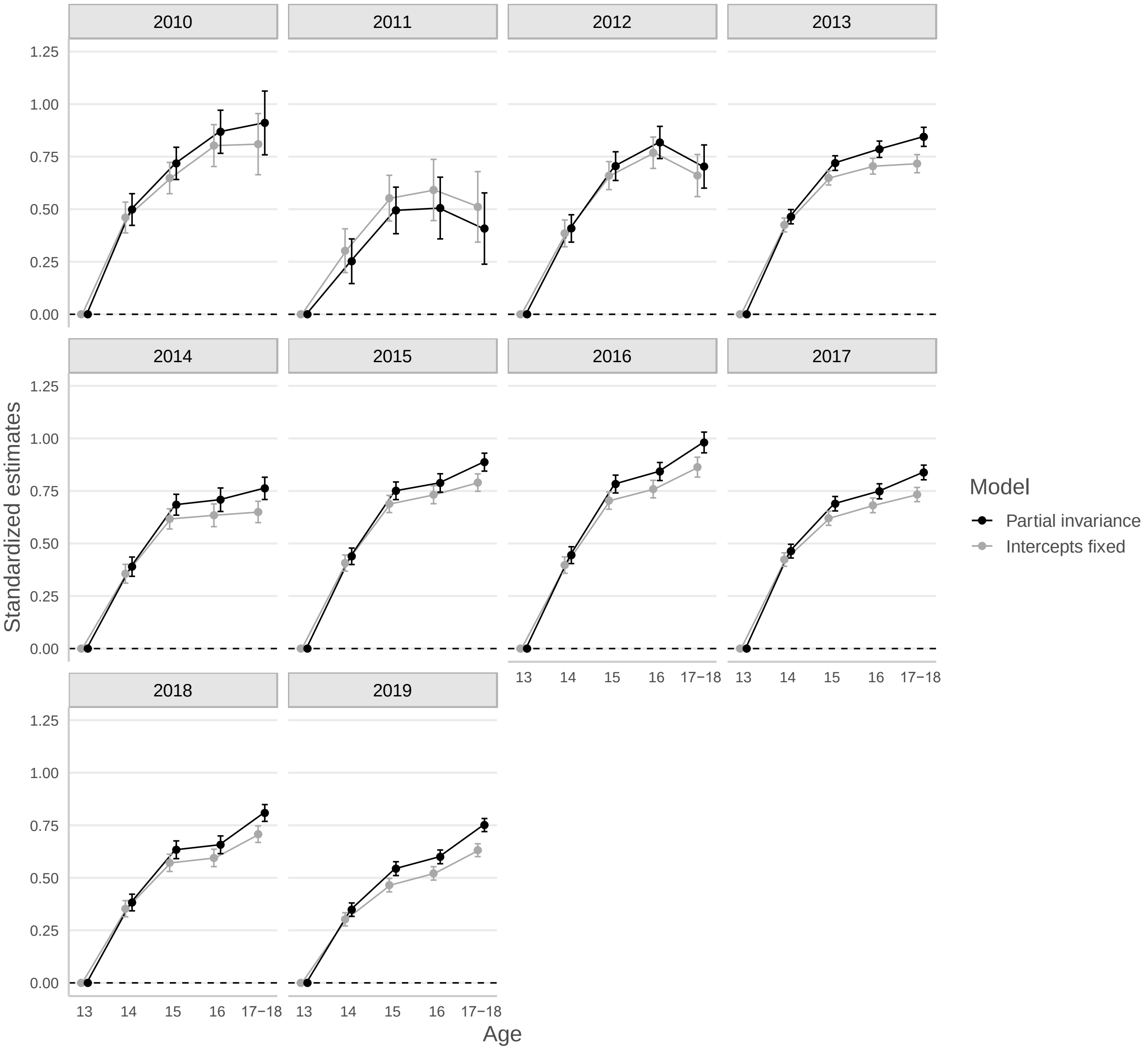

Note. This figure shows latent means across age groups (reference 13-year-olds), comparing models accounting (partial invariance; black) and not accounting (intercepts fixed) for non-invariant intercept(s)

Gender invariance

Supplementary Table 32. Measurement invariance across gender, pooled sample (across all survey years).

**Model**	$\chi2$ (df)	p	CFI	RMSEA (90 %CI)	TLI	SRMR	ΔCFI	ΔRMSEA
Baseline model	22402.254 (18)	<.001	0.994	0.065 (0.065, 0.066)	0.991	0.022		
Baseline model*	12782.368 (16)	<.001	0.997	0.052 (0.052, 0.053)	0.994	0.017	0.00242	-0.013
Thresholds	14084.968 (22)	<.001	0.996	0.047 (0.046, 0.048)	0.995	0.017	-0.00033	-0.005
Thresholds and loadings gender	13219.173 (27)	<.001	0.997	0.041 (0.040, 0.042)	0.996	0.017	0.00022	-0.006
Thresholds, loadings, intercepts gender	25976.473 (32)	<.001	0.993	0.053 (0.052, 0.053)	0.994	0.018	-0.0032	0.012
Partial scalar invariance; freeing intercepts item 6	18500.049 (31)	<.001	0.995	0.045 (0.045, 0.046)	0.996	0.018	-0.0013	0.004

Note. χ^2= chi-square goodness-of-fit based on the Satorra-Bentler correction; df = degrees of freedom; CFI; comparative fit index; RMSEA = root mean square error of approximation; CI = confidence interval. TLI = Tucker Lewis Index. SRMR = standardized root mean squared residual; ΔCFI/RMSEA = Change in CFI/RMSEA between models.
* Models accounting for one correlated error term between item 3 and 4 (both boys and girls).

Supplementary Table 33. Intercept means and d_{MACS} effect size of noninvariant intercepts of item 6, partial invariance model.

Items	Gender	Intercept mean (95% CI)	d_{MACS}
Item 6	Boys	**ref.**	**ref.**
Item 6	Girls	0.171 (0.167, 0.176)	0.178

Supplementary Table 34. Latent means by gender (pooled sample).
Comparing models not accounting (Intercepts fixed) and accounting (partial invariance) for non-invariant parameters

	Intercepts fixed		Partial invariance	
	β (95% CI)	p	β (95% CI)	p
Boys	**ref**		**ref**	
Girls	0.727 (0.721, 0.734)	<0.001	0.673 (0.666, 0.679)	<0.001

Note. Ref. = reference group (gender = Boys). β = standardized regression coefficient. 95% CI =95 % confidence intervals of β .

Supplementary Table 35. Measurement invariance across gender by survey year.

Model	χ2 (df)	p	CFI	RMSEA (90 %CI)	TLI	SRMR	ΔCFI	ΔRMSEA
**2010**								
Baseline	775.632 (18)	<.001	0.993	0.07 (0.066, 0.074)	0.989	0.023		
Baseline*	538.7 (16)	<.001	0.995	0.062 (0.057, 0.066)	0.991	0.02		
Thresholds	592.547 (22)	<.001	0.995	0.055 (0.051, 0.059)	0.993	0.02	-0.0004	-0.0067
Thresholds and loadings	525.609 (27)	<.001	0.996	0.046 (0.043, 0.05)	0.995	0.02	0.0006	-0.0086
Thresholds. loadings and intercepts	938.17 (32)	<.001	0.992	0.058 (0.054, 0.061)	0.993	0.02	-0.0036	0.0111
Thresholds. loadings and intercepts freeing intercept item 6	704.384 (31)	<.001	0.994	0.05 (0.047, 0.054)	0.994	0.02	-0.0015	0.0039
**2011**								
Baseline	408.163 (18)	<.001	0.994	0.061 (0.056, 0.067)	0.991	0.022		
Baseline*	331.907 (16)	<.001	0.995	0.059 (0.053, 0.064)	0.991	0.02		
Thresholds	342.26 (22)	<.001	0.995	0.05 (0.046, 0.055)	0.994	0.02	-0.0001	-0.0083
Thresholds and loadings	352.229 (27)	<.001	0.995	0.046 (0.042, 0.05)	0.995	0.02	-0.0001	-0.0046
Thresholds, loadings, intercepts	595.719 (32)	<.001	0.992	0.055 (0.051, 0.059)	0.992	0.021	-0.0035	0.0096
Thresholds, loadings, intercepts freeing intercepts item 6	479.636 (31)	<.001	0.993	0.05 (0.046, 0.054)	0.994	0.021	-0.0018	0.0044
**2012**								
Baseline	1173.025 (18)	<.001	0.993	0.074 (0.07, 0.078)	0.988	0.024		
Baseline*	796.061 (16)	<.001	0.995	0.064 (0.061, 0.068)	0.991	0.02		
Thresholds	853.053 (22)	<.001	0.995	0.057 (0.054, 0.06)	0.993	0.02	-0.0003	-0.0077
Thresholds and loadings	740.273 (27)	<.001	0.995	0.047 (0.045, 0.05)	0.995	0.02	0.0008	-0.0093
Thresholds, loadings, intercepts	1232.869 (32)	<.001	0.992	0.057 (0.054, 0.059)	0.993	0.021	-0.0031	0.0091
Thresholds, loadings, intercepts freeing intercepts item 2 & 6	944.603 (30)	<.001	0.994	0.051 (0.048, 0.054)	0.994	0.021	-0.0013	0.0035
**2013**								
Baseline	3531.003 (18)	<.001	0.993	0.07 (0.068, 0.072)	0.989	0.024		
Baseline*	2301.628 (16)	<.001	0.996	0.06 (0.058, 0.062)	0.992	0.019		
Thresholds	2467.233 (22)	<.001	0.995	0.053 (0.051, 0.055)	0.994	0.019	-0.0003	-0.0071
Thresholds and loadings	2115.185 (27)	<.001	0.996	0.044 (0.043, 0.046)	0.996	0.019	0.0007	-0.0088
Thresholds, loadings, intercepts	3761.722 (32)	<.001	0.993	0.054 (0.053, 0.056)	0.993	0.02	-0.0031	0.01
Thresholds, loadings, intercepts freeing intercepts item 6	2856.47 (31)	<.001	0.995	0.048 (0.046, 0.049)	0.995	0.02	-0.0014	0.0038
**2014**								
Baseline	1690.43 (18)	<.001	0.994	0.065 (0.063, 0.068)	0.99	0.023		
Baseline*	1031.087 (16)	<.001	0.996	0.054 (0.051, 0.057)	0.993	0.018		
Thresholds	1129.878 (22)	<.001	0.996	0.048 (0.046, 0.051)	0.994	0.018	-0.0004	-0.0059

Thresholds and loadings	1073.969 (27)	<.001	0.996	0.042 (0.04, 0.044)	0.996	0.019	0.0002	-0.0059
Thresholds, loadings and intercepts	1829.213 (32)	<.001	0.993	0.051 (0.049, 0.053)	0.994	0.02	-0.0028	0.0086
Thresholds, loadings, intercepts freeing intercepts item 6	1322.508 (31)	<.001	0.995	0.044 (0.042, 0.046)	0.995	0.019	-0.0009	0.0015
**2015**								
Baseline	2180.68 (18)	<.001	0.995	0.06 (0.058, 0.062)	0.992	0.02		
Baseline*	1323.1 (16)	<.001	0.997	0.05 (0.047, 0.052)	0.995	0.016		
Thresholds	1421.294 (22)	<.001	0.997	0.044 (0.042, 0.046)	0.996	0.016	-0.0002	-0.0058
Thresholds and loadings	1323.019 (27)	<.001	0.997	0.038 (0.036, 0.04)	0.997	0.016	0.0002	-0.0057
Thresholds, loadings, intercepts	2670.506 (32)	<.001	0.994	0.05 (0.048, 0.051)	0.995	0.017	-0.0029	0.0118
Thresholds, loadings, intercepts freeing intercepts item 6	1956.949 (31)	<.001	0.996	0.043 (0.042, 0.045)	0.996	0.017	-0.0014	0.0052
**2016**								
Baseline	2324.364 (18)	<.001	0.995	0.063 (0.061, 0.065)	0.991	0.022		
Baseline*	1294.273 (16)	<.001	0.997	0.05 (0.047, 0.052)	0.994	0.017		
Thresholds	1386.896 (22)	<.001	0.997	0.044 (0.042, 0.046)	0.996	0.017	-0.0002	-0.0059
Thresholds and loadings	1350.321 (27)	<.001	0.997	0.039 (0.037, 0.041)	0.997	0.017	0.0001	-0.0049
Thresholds, loadings, intercepts	2764.655 (32)	<.001	0.994	0.051 (0.05, 0.053)	0.994	0.019	-0.0033	0.0125
Thresholds, loadings, intercepts freeing intercepts item 6	1967.336 (31)	<.001	0.996	0.044 (0.042, 0.046)	0.996	0.018	-0.0014	0.005
**2017**								
Baseline	3752.388 (18)	<.001	0.994	0.066 (0.064, 0.067)	0.991	0.022		
Baseline*	1909.698 (16)	<.001	0.997	0.05 (0.048, 0.051)	0.995	0.016		
Thresholds	2201.458 (22)	<.001	0.997	0.045 (0.044, 0.047)	0.996	0.016	-0.0004	-0.0042
Thresholds and loadings	2113.584 (27)	<.001	0.997	0.04 (0.039, 0.041)	0.997	0.016	0.0001	-0.0053
Thresholds, loadings, and intercepts	4997.786 (32)	<.001	0.993	0.057 (0.055, 0.058)	0.993	0.018	-0.0043	0.0167
Thresholds, loadings, and intercepts partial:free intercepts 2 & 6	2963.01 (30)	<.001	0.996	0.045 (0.044, 0.046)	0.996	0.017	-0.0013	0.005
**2018**								
Baseline	2665.612 (18)	<.001	0.994	0.066 (0.064, 0.069)	0.99	0.021		
Baseline**	1398.595 (16)	<.001	0.997	0.051 (0.049, 0.053)	0.994	0.016		
Thresholds	1548.7 (22)	<.001	0.997	0.046 (0.044, 0.048)	0.996	0.016	-0.0003	-0.0053
Thresholds and loadings	1463.097 (27)	<.001	0.997	0.04 (0.038, 0.042)	0.997	0.016	0.0002	-0.0057
Thresholds, loadings, intercepts	3589.377 (32)	<.001	0.992	0.058 (0.056, 0.059)	0.993	0.018	-0.0046	0.0178
Thresholds, loadings, intercepts partial free intercepts item 2 & 6	1968.388 (30)	<.001	0.996	0.044 (0.042, 0.046)	0.996	0.017	-0.0011	0.0041
**2019**								
Baseline	4509.661 (18)	<.001	0.994	0.067 (0.065, 0.069)	0.991	0.021		
Baseline*	2296.886 (16)	<.001	0.997	0.051 (0.049, 0.052)	0.995	0.016		
Thresholds	2610.573 (22)	<.001	0.997	0.046 (0.045, 0.048)	0.996	0.016	-0.0004	-0.0046

Thresholds and loadings	2532.635 (27)	<.001	0.997	0.041 (0.04, 0.042)	0.996	0.016	0.0001	-0.0052
Thresholds loadings intercepts	5840.526 (32)	<.001	0.993	0.057 (0.056, 0.058)	0.993	0.018	-0.0042	0.0163
Thresholds, loadings, intercepts partial free intercepts item 2 & 6	3552.022 (30)	<.001	0.996	0.046 (0.045, 0.047)	0.996	0.017	-0.0013	0.0051

Note. χ^2= chi-square goodness-of-fit based on the Satorra-Bentler correction; df = degrees of freedom; CFI; comparative fit index; RMSEA = root mean square error of approximation; CI = confidence interval. TLI = Tucker Lewis Index. SRMR = standardized root mean squared residual; ΔCFI/RMSEA = Change in CFI/RMSEA between models.

* Models accounting for one correlated error term between item 3 and 4.

Supplementary Table 36. Partial gender invariance across survey years: Intercept means and d_{MACS} effect size of noninvariant intercepts

Year	Items	Gender	Intercept mean (95% CI)	d_{MACS}
2010	Item 6	Girls (ref. Boys)	0.171 (0.147, 0.195)	0.180
2011	Item 6	Girls (ref. Boys)	0.164 (0.132, 0.196)	0.170
2012	Item 2	Girls (ref. Boys)	-0.114 (-0.139, -0.089)	0.115
	Item 6	Girls (ref. Boys)	0.111 (0.090, 0.133)	0.116
2013	Item 6	Girls (ref. Boys)	0.168 (0.156, 0.179)	0.173
2014	Item 6	Girls (ref. Boys)	0.173 (0.156, 0.189)	0.178
2015	Item 6	Girls (ref. Boys)	0.159 (0.146, 0.172)	0.165
2016	Item 6	Girls (ref. Boys)	0.176 (0.163, 0.190)	0.181
2017	Item 2	Girls (ref. Boys)	-0.131 (-0.144, -0.118)	0.136
	Item 6	Girls (ref. Boys)	0.157 (0.146, 0.168)	0.161
2018	Item 2	Girls (ref. Boys)	-0.124 (-0.139, -0.109)	0.128
	Item 6	Girls (ref. Boys)	0.174 (0.161, 0.187)	0.181
2019	Item 2	Girls (ref. Boys)	-0.122 (-0.133, -0.110)	0.125
	Item 6	Girls (ref. Boys)	0.154 (0.144,0.164)	0.162

Supplementary Table 37. Latent means by gender per survey year. Comparing models not accounting (Intercepts fixed) and accounting (partial invariance) for non-invariant parameters

		Intercepts fixed		Partial invariance	
Year	Gender	β (95% CI)	p	β (95% CI)	p
2010	Boys	ref.		ref.	
	Girls	0.510 (0.474, 0.547)	<0.001	0.456 (0.419, 0.493)	<0.001
2011	Boys	ref.		ref.	
	Girls	0.634 (0.589, 0.678)	<0.001	0.585 (0.539, 0.63)	<0.001
2012	Boys	ref.		ref.	
	Girls	0.626 (0.594, 0.657)	<0.001	0.614 (0.582, 0.646)	<0.001
2013	Boys	ref.		ref.	
	Girls	0.699 (0.682, 0.716)	<0.001	0.648 (0.631, 0.665)	<0.001
2014	Boys	ref.		ref.	
	Girls	0.729 (0.706, 0.752)	<0.001	0.674 (0.650, 0.697)	<0.001
2015	Boys	ref.		ref.	
	Girls	0.729 (0.711, 0.748)	<0.001	0.678 (0.659, 0.697)	<0.001
2016	Boys	ref.		ref.	
	Girls	0.767 (0.748, 0.786)	<0.001	0.711 (0.692, 0.73)	<0.001
2017	Boys	ref.		ref.	
	Girls	0.765 (0.749, 0.780)	<0.001	0.736 (0.720, 0.752)	<0.001
2018	Boys	ref.		ref.	
	Girls	-0.709 (-0.727, -0.692)	<0.001	0.697 (0.678, 0.716)	<0.001
2019	Boys	ref.		ref.	
	Girls	0.762 (0.748, 0.777)	<0.001	0.734 (0.719, 0.749)	<0.001

Note. Ref. = reference group (gender = Boys). β = standardized regression coefficient. 95% CI =95 % confidence intervals of β .

Supplementary Figure 7. The practical consequences of non-invariance on differences in latent means between boys and girls, by survey year.

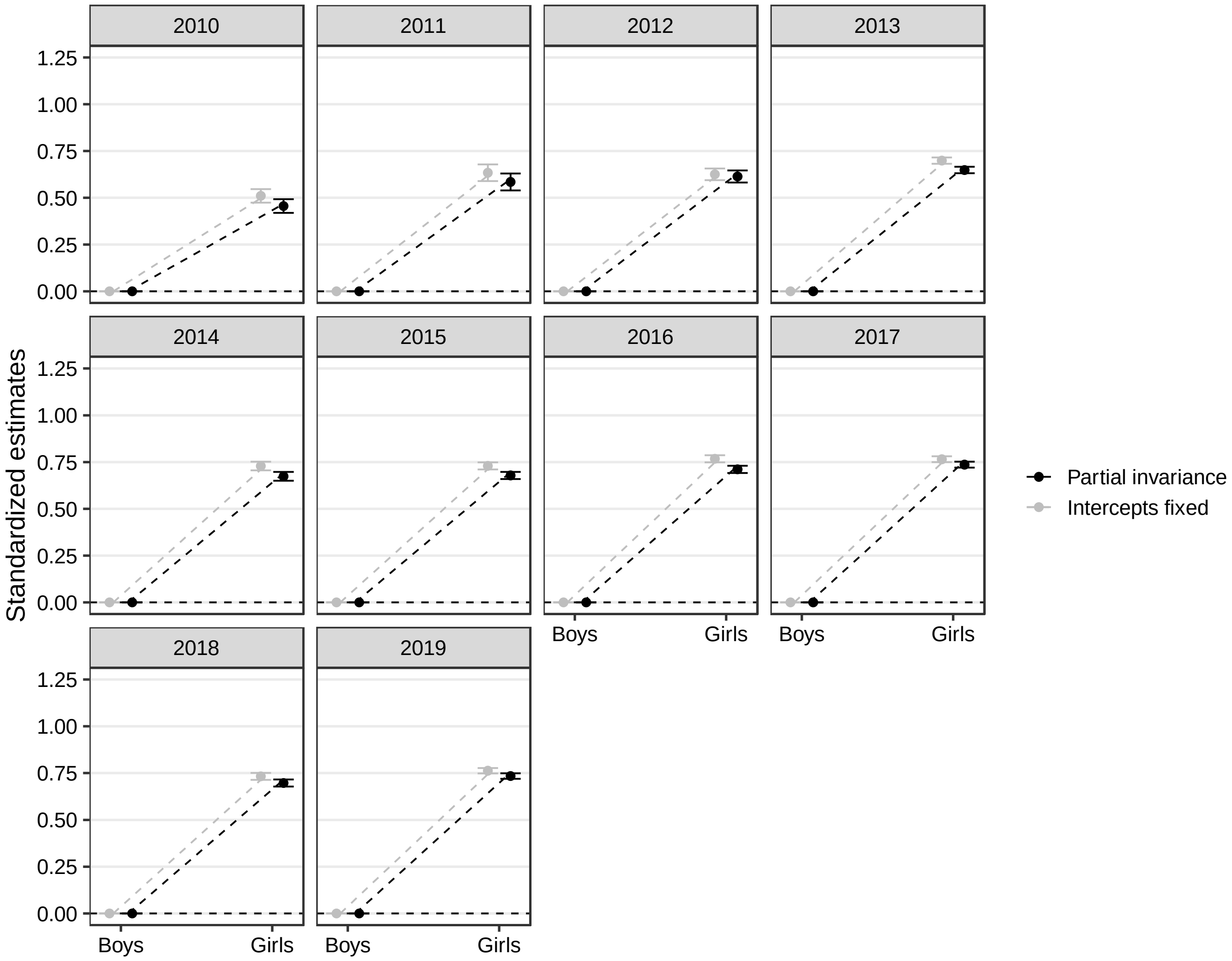

Note. This figure shows differences latent means (in standardized deviation units) by gender (reference Boys), comparing models accounting (partial invariance; black) and not accounting (intercepts fixed) for non-invariant intercept(s)

Sensitivity analyses

Supplementary Table 38. Sensitivity analyses: Measurement invariance across time by age groups among girls when excluding year 2010

**Model**	χ^2 (*df*)	p	CFI	RMSEA (90% CI)	TLI	SRMR	ΔCFI	ΔRMSEA
Age 13								
Baseline	1918.29964(81)	<.001	0.996	0.059 (0.057, 0.062)	0.993	0.020		
Baseline*	1078.77407(72)	<.001	0.998	0.047 (0.044, 0.049)	0.995	0.015		
Thresholds	1175.34715(120)	<.001	0.997	0.037 (0.035, 0.039)	0.997	0.015	-0.0001	-0.0097
Thresholds and loadings	1030.24532(160)	<.001	0.998	0.029 (0.027, 0.031)	0.998	0.015	0.0004	-0.0079
Thresholds, loadings, intercepts	1714.21565(200)	<.001	0.996	0.034 (0.033, 0.036)	0.998	0.016	-0.0015	0.0052
Age 14								
Baseline	2342.63168(81)	<.001	0.995	0.067 (0.064, 0.069)	0.991	0.021		
Baseline*	1181.17774(72)	<.001	0.997	0.050 (0.047, 0.052)	0.995	0.016		
Thresholds	1278.72522(120)	<.001	0.997	0.039 (0.037, 0.041)	0.997	0.016	-0.0001	-0.0103
Thresholds and loadings	1127.37033(160)	<.001	0.998	0.031 (0.029, 0.033)	0.998	0.016	0.0005	-0.0082
Thresholds, loadings, intercepts	1821.53036(200)	<.001	0.996	0.036 (0.034, 0.037)	0.997	0.016	-0.0016	0.0049
Age 15								
Baseline	2643.49877(81)	<.001	0.994	0.071 (0.068, 0.073)	0.989	0.023		
Baseline*	1332.93084(72)	<.001	0.997	0.053 (0.050, 0.055)	0.994	0.017		
Thresholds	1426.51936(120)	<.001	0.997	0.042 (0.040, 0.043)	0.996	0.017	-0.0001	-0.0111
Thresholds and loadings	1197.34136(160)	<.001	0.997	0.032 (0.030, 0.034)	0.998	0.017	0.0007	-0.0095
Thresholds, loadings, intercepts	2143.10952(200)	<.001	0.995	0.039 (0.038, 0.041)	0.997	0.017	-0.0023	0.0072
Partial scalar invariance	1546.92984(192)	<.001	0.997	0.033 (0.032, 0.035)	0.998	0.017	-0.0008	0.0014
Age 16								
Baseline	2696.00887(81)	<.001	0.993	0.076 (0.074, 0.078)	0.988	0.024		
Baseline*	1463.23452(72)	<.001	0.996	0.059 (0.056, 0.061)	0.993	0.018		
Thresholds	1526.83283(120)	<.001	0.996	0.046 (0.044, 0.048)	0.996	0.018	<-0.0001	-0.013
Thresholds and loadings	1345.86854(160)	<.001	0.997	0.036 (0.035, 0.038)	0.997	0.018	0.0006	-0.0094
Thresholds, loadings, intercepts	1965.92522(200)	<.001	0.995	0.040 (0.038, 0.041)	0.997	0.019	-0.0015	0.0033
Age 17-18								
Baseline	2928.30946(81)	<.001	0.993	0.075 (0.073, 0.077)	0.988	0.024		
Baseline*	1811.34914(72)	<.001	0.996	0.062 (0.060, 0.065)	0.992	0.020		
Thresholds	1885.3213(120)	<.001	0.996	0.048 (0.047, 0.050)	0.995	0.020	-0.0001	-0.0136
Thresholds and loadings	1562.76253(160)	<.001	0.996	0.037 (0.036, 0.039)	0.997	0.020	0.0009	-0.0111

Thresholds, loadings, intercepts	2123.27415(200)	<.001	0.995	0.039 (0.038, 0.041)	0.997	0.020	-0.0013	0.0018

Note. χ^2= chi-square goodness-of-fit based on the Satorra-Bentler correction; df = degrees of freedom; CFI; comparative fit index; RMSEA = root mean square error of approximation; CI = confidence interval. TLI = Tucker Lewis Index. SRMR = standardized root mean squared residual; ΔCFI/RMSEA = Change in CFI/RMSEA between models. * Models accounting for one correlated error term between item 3 and 4.

Supplementary Table 39. Sensitivity analyses: Measurement invariance across time by age groups among boys when excluding year 2010.

**Model**	χ^2 (*df*)	p	CFI	RMSEA (90% CI)	TLI	SRMR	ΔCFI	ΔRMSEA
Age 13								
Baseline	1733.46054(81)	<.001	0.993	0.057 (0.055, 0.06)	0.989	0.024		
Baseline*	1114.02297(72)	<.001	0.996	0.048 (0.046, 0.051)	0.992	0.019		
Thresholds	1173.47053(120)	<.001	0.996	0.038 (0.036, 0.039)	0.995	0.019	<-0.0001	-0.0106
Thresholds and loadings	999.29993(160)	<.001	0.997	0.029 (0.027, 0.031)	0.997	0.019	0.0009	-0.0085
Thresholds, loadings, intercepts	1393.02292(200)	<.001	0.995	0.031 (0.029, 0.032)	0.997	0.020	-0.0014	0.0019
Age 14								
Baseline	2108.80341(81)	<.001	0.992	0.064 (0.061, 0.066)	0.987	0.025		
Baseline*	1263.57763(72)	<.001	0.996	0.052 (0.049, 0.054)	0.992	0.019		
Thresholds	1310.19269(120)	<.001	0.996	0.04 (0.038, 0.042)	0.995	0.019	<-0.0001	-0.0117
Thresholds and loadings	1130.90602(160)	<.001	0.996	0.031 (0.03, 0.033)	0.997	0.020	0.0008	-0.0088
Thresholds, loadings, intercepts	1626.7697(200)	<.001	0.995	0.034 (0.033, 0.036)	0.996	0.021	-0.0017	0.0026
Age 15								
Baseline	2228.79333(81)	<.001	0.993	0.065 (0.063, 0.068)	0.988	0.024		
Baseline*	1237.00835(72)	<.001	0.996	0.051 (0.048, 0.053)	0.993	0.018		
Thresholds	1292.17644(120)	<.001	0.996	0.04 (0.038, 0.042)	0.996	0.018	<-0.0001	-0.0114
Thresholds and loadings	1079.43781(160)	<.001	0.997	0.03 (0.029, 0.032)	0.997	0.018	0.0008	-0.0092
Thresholds, loadings, intercepts	1755.14764(200)	<.001	0.995	0.035 (0.034, 0.037)	0.997	0.019	-0.0021	0.005
Partial scalar invariance	1369.67567(192)	<.001	0.996	0.031 (0.03, 0.033)	0.997	0.019	-0.0008	0.001
Age 16								
Baseline	2373.79141(81)	<.001	0.993	0.071 (0.069, 0.074)	0.988	0.025		
Baseline*	1460.05843(72)	<.001	0.996	0.059 (0.056, 0.061)	0.992	0.020		
Thresholds	1510.61225(120)	<.001	0.996	0.046 (0.044, 0.048)	0.995	0.020	<-0.0001	-0.0132
Thresholds and loadings	1249.07511(160)	<.001	0.997	0.035 (0.033, 0.037)	0.997	0.020	0.0009	-0.0106
Thresholds, loadings, intercepts	1882.68404(200)	<.001	0.995	0.039 (0.037, 0.04)	0.996	0.020	-0.0018	0.0039
Age 17-18								
Baseline	2249.8873(81)	<.001	0.994	0.069 (0.066, 0.071)	0.99	0.024		
Baseline*	1448.88257(72)	<.001	0.996	0.058 (0.056, 0.061)	0.993	0.019		

Thresholds	1461.69114(120)	<.001	0.996	0.044 (0.042, 0.047)	0.996	0.019	0.0001	-0.0137
Thresholds and loadings	1276.66966(160)	<.001	0.997	0.035 (0.033, 0.037)	0.997	0.019	0.0006	-0.0093
Thresholds, loadings, intercepts	1520.6923(200)	<.001	0.996	0.034 (0.033, 0.036)	0.997	0.019	-0.0006	-0.001

Note. χ^2= chi-square goodness-of-fit based on the Satorra-Bentler correction; df = degrees of freedom; CFI; comparative fit index; RMSEA = root mean square error of approximation; CI = confidence interval. TLI = Tucker Lewis Index. SRMR = standardized root mean squared residual; ΔCFI/RMSEA = Change in CFI/RMSEA between models. * Models accounting for one correlated error term between item 3 and 4.

Supplementary Table 40. Sensitivity analyses: Measurement invariance comparing 2010 and 2019 among girls.

**Model**	χ^2(df)	p	CFI	RMSEA (90%CI)	TLI	SRMR	ΔCFI	ΔRMSEA
Age 13								
Baseline*	369.85976(16)	<.001	0.998	0.047 (0.043, 0.051)	0.996	0.015		
Thresholds	394.85768(22)	<.001	0.998	0.041 (0.037, 0.044)	0.997	0.015	-0.0001	-0.0058
Thresholds and loadings	370.96601(27)	<.001	0.998	0.035 (0.032, 0.039)	0.997	0.015	0.0002	-0.0054
Thresholds, loadings, intercepts	558.04561(32)	<.001	0.997	0.04 (0.037, 0.043)	0.997	0.015	-0.0012	0.0048
Age 15								
Baseline*	493.78065(16)	<.001	0.997	0.056 (0.051, 0.06)	0.994	0.017		
Thresholds	499.35442(22)	<.001	0.997	0.047 (0.044, 0.051)	0.995	0.017	<0.0001	-0.0082
Thresholds and loadings	417.93334(27)	<.001	0.997	0.039 (0.035, 0.042)	0.997	0.017	0.0006	-0.0087
Thresholds, loadings, intercepts	705.02924(32)	<.001	0.995	0.047 (0.044, 0.05)	0.996	0.017	-0.002	0.0079
Age 17-18								
Baseline*	623.94801(16)	<.001	0.996	0.062 (0.058, 0.067)	0.992	0.018		
Thresholds	634.2912(22)	<.001	0.996	0.053 (0.05, 0.057)	0.994	0.018	<0.0001	-0.009
Thresholds and loadings	532.62029(27)	<.001	0.996	0.044 (0.041, 0.047)	0.996	0.018	0.0008	-0.0096
Thresholds, loadings, intercepts	698.47301(32)	<.001	0.995	0.046 (0.043, 0.049)	0.995	0.018	-0.0012	0.0024

Note. χ^2= chi-square goodness-of-fit based on the Satorra-Bentler correction; df = degrees of freedom; CFI; comparative fit index; RMSEA = root mean square error of approximation; CI = confidence interval. TLI = Tucker Lewis Index. SRMR = standardized root mean squared residual; ΔCFI/RMSEA = Change in CFI/RMSEA between models.
* Models accounting for one correlated error term between item 3 and 4.

Supplementary Table 41. Sensitivity analyses: Measurement invariance comparing 2010 and 2019 among boys.

**Model**	χ^2(df)	p	CFI	RMSEA (90%CI)	TLI	SRMR	ΔCFI	ΔRMSEA
Age 13								
Baseline*	453.01037(16)	<.001	0.995	0.053 (0.049, 0.057)	0.991	0.020		
Thresholds	475.8948(22)	<.001	0.995	0.046 (0.042, 0.049)	0.993	0.020	-0.0002	-0.0069
Thresholds and loadings	405.64666(27)	<.001	0.996	0.038 (0.035, 0.041)	0.995	0.021	0.0008	-0.008
Thresholds, loadings, intercepts	672.16527(32)	<.001	0.993	0.045 (0.042, 0.048)	0.993	0.022	-0.0029	0.0073
Partial scalar invariance (free intercept item 5)	540.81732(31)	<.001	0.994	0.041 (0.038, 0.044)	0.994	0.021	-0.0015	0.0031
Age 15								
Baseline*	382.74171(16)	<.001	0.996	0.049 (0.045, 0.054)	0.993	0.017		
Thresholds	386.31212(22)	<.001	0.997	0.042 (0.038, 0.046)	0.995	0.017	<0.0001	-0.0074
Thresholds and loadings	316.67543(27)	<.001	0.997	0.034 (0.03, 0.037)	0.997	0.017	0.0007	-0.0082
Thresholds, loadings, intercepts	722.53277(32)	<.001	0.993	0.048 (0.045, 0.051)	0.994	0.018	-0.0039	0.0141
Partial scalar invariance (free intercept item 5)	448.54596(31)	<.001	0.996	0.038 (0.035, 0.041)	0.996	0.017	-0.0012	0.0041
Age 17-18								
Baseline*	525.17449(16)	<.001	0.996	0.06 (0.055, 0.064)	0.993	0.019		
Thresholds	512.47829(22)	<.001	0.996	0.05 (0.046, 0.054)	0.995	0.019	0.0001	-0.0097
Thresholds and loadings	427.82888(27)	<.001	0.997	0.041 (0.037, 0.044)	0.997	0.019	0.0007	-0.0092
Thresholds, loadings, intercepts	538.47929(32)	<.001	0.996	0.042 (0.039, 0.045)	0.996	0.019	-0.0008	0.0013

Note. χ^2= chi-square goodness-of-fit based on the Satorra-Bentler correction; df = degrees of freedom; CFI; comparative fit index; RMSEA = root mean square error of approximation; CI = confidence interval. TLI = Tucker Lewis Index. SRMR = standardized root mean squared residual; ΔCFI/RMSEA = Change in CFI/RMSEA between models.
* Models accounting for one correlated error term between item 3 and 4.

Supplementary Table 42. Sensitivity analyses: Intercept means and d_{MACS} effect size of noninvariant intercepts of item 5, partial invariance model. Comparing 2013 and 2019 among 13 and 15 year old boys.

Year	Intercept mean (95% CI)	d_{MACS}	Age
2010	ref.	ref.	
2019	-0.171 (-0.205,-0.136)	0.151	13
2019	-0.206 (-0.237,-0.176)	0.200	15

Supplementary Table 43. Sensitivity analyses: Latent means among boys between years 2013 and 2019. Comparing models not accounting (Intercepts fixed) and accounting (partial invariance) for non-invariant parameters.

		Intercepts fixed		Partial invariance	
Age	Year	β (95% CI)	p	β (95% CI)	p
13-year-olds	2013	ref.		ref.	
	2019	0.206 (0.171, 0.241)	<0.001	0.229 (0.194, 0.264)	<0.001
15-year-olds	2013	ref.		ref.	
	2019	0.132 (0.100, 0.164)	<0.001	0.161 (0.129, 0.193)	<0.001

Note. Ref. = reference group (survey year = 2013). β = standardized regression coefficient. 95% CI =95 % confidence intervals of β .

Missing data assessment

Our main analyses assessing measurement invariance across time/survey years by gender and age groups, were re-evaluated using multiple imputed data, to assess the extent to which our main results were sensitive to different methods of dealing with missing data.

Using the *mice* R-package (van Buuren & Groothuis-Oudshoorn, 2011) under the missing at random (MAR) assumption, we performed 15 imputations with 10 iterations each. The following auxiliary variables were added to inform the imputation model, in addition to survey year, age, gender and the DMI items:

- Maternal and paternal education: *Did your father and mother go to university or to a university college? (Yes/no)*. Separate answers for mother and father given.
- Perceived family finances: *Financially, has your family been well off, or badly off, over the past two years? (We have been well off the whole time, We have generally been well off, We have neither been well off nor badly off, We have generally been badly off, We have been badly off the whole time*).
- Geographical region (*Eastern Norway, Western Norway, Northern Norway, Central Norway, and Southern Norway*)
- Municipality population size (*categorized into <5,000, 5000–9,999, 10,000–19,999, 20,000–49,999, 50,000 +*)

The municipality level indicator (nominal variable with 418 levels) were not added to the imputation model, as it would render the imputation model too complex to estimate. Proportional odds models were used to predict missing values for the DMI items, perceived family finances, municipality population size and age, to properly account for their ordinal nature, whereas (polytomous) logistic regression models were used to predict missing values for the nominal variables geograpical region and gender.

Convergence of the imputation model was checked by traceplots and by checking that no impossible values were imputed, none of which suggested any problems.

Analytical strategy

As an initial check of whether scores on the DMI items varied between multiple imputed data and observed data (i.e., complete case analysis), we compared means and standard deviations of each items between the two approaches. As shown in Supplementary Table 47 and 48 (below), the item scores were almost identical.

We next re-estimated our main Multi-group confirmatory factor analysis (MGCFA) assessing measurement invariance across time/survey years using multiple imputed data. Functions from the R-packages *lavaan* (Rosseel, 2012) and *semTools* (Jorgensen et al., 2020) were used to fit the MG-CFA models on the imputed datasets and to extract pooled fit indices and parameter estimates following Rubin's rules (Rubin, 1987).

Fit indices of the nested models assessing measurement invariance among girls and boys are shown in Supplementary Tables 46-47. Trends in latent mean scores among girls and boys by age groups are shown in Supplementary Figures 8 and 9, and the numerical estimates of differences in standardized latent means across survey years are shown in Supplementary Tables 48-49.

Compared to the main results reported in the paper (based on pairwise deletion), the results based on multiple imputed data were nearly identical in terms of latent mean trends across time for boys and girls and across age groups. Model fit indices were also highly similar, although three extra partial invariance models were examined (among 13-year old boys and 15- and 17-18 year old girls). However, none of these models led to any new information, as the magnitude of noninvariance was again very small and had virtually no impact on trend estimates in latent mean scores.

Supplementary Table 44. Sensitivity analyses: Comparing imputed item values with observed item values across time; **Boys**.

	2010	2011	2012	2013	2014	2015	2016	2017	2018	2019
**Item 1 (M[SD])**										
Imputed	2.08 (0.96)	1.86 (0.88)	2.00 (0.94)	1.91 (0.90)	1.89 (0.90)	1.91 (0.93)	1.89 (0.91)	1.94 (0.93)	2.00 (0.96)	1.99 (0.95)
Observed	2.08 (0.96)	1.86 (0.88)	2.00 (0.94)	1.91 (0.90)	1.88 (0.90)	1.90 (0.92)	1.88 (0.91)	1.93 (0.93)	1.99 (0.96)	1.98 (0.95)
**Item 2 (M[SD])**										
Imputed	1.89 (0.95)	1.76 (0.87)	1.90 (0.94)	1.83 (0.90)	1.80 (0.89)	1.83 (0.91)	1.81 (0.90)	1.88 (0.92)	1.93 (0.95)	1.96 (0.96)
Observed	1.89 (0.95)	1.76 (0.87)	1.90 (0.93)	1.82 (0.90)	1.80 (0.89)	1.83 (0.90)	1.81 (0.90)	1.88 (0.92)	1.92 (0.95)	1.95 (0.96)
**Item 3 (M[SD])**										
Imputed	1.64 (0.85)	1.54 (0.79)	1.62 (0.83)	1.59 (0.82)	1.57 (0.82)	1.59 (0.83)	1.58 (0.82)	1.69 (0.88)	1.73 (0.91)	1.78 (0.92)
Observed	1.64 (0.85)	1.54 (0.78)	1.61 (0.82)	1.58 (0.81)	1.56 (0.81)	1.59 (0.83)	1.57 (0.82)	1.68 (0.87)	1.73 (0.90)	1.77 (0.92)
**Item 4 (M[SD])**										
Imputed	1.63 (0.89)	1.52 (0.81)	1.61 (0.86)	1.57 (0.84)	1.57 (0.85)	1.61 (0.88)	1.58 (0.86)	1.67 (0.91)	1.73 (0.94)	1.75 (0.95)
Observed	1.63 (0.88)	1.52 (0.81)	1.61 (0.86)	1.57 (0.84)	1.56 (0.85)	1.60 (0.87)	1.57 (0.85)	1.66 (0.91)	1.72 (0.94)	1.74 (0.95)
**Item 5 (M[SD])**										
Imputed	1.78 (0.90)	1.64 (0.80)	1.71 (0.85)	1.68 (0.84)	1.61 (0.80)	1.62 (0.81)	1.58 (0.79)	1.68 (0.84)	1.68 (0.86)	1.70 (0.86)
Observed	1.78 (0.90)	1.64 (0.80)	1.71 (0.85)	1.68 (0.83)	1.61 (0.80)	1.62 (0.81)	1.57 (0.78)	1.67 (0.83)	1.67 (0.85)	1.69 (0.85)
**Item 6 (M[SD])**										
Imputed	1.90 (0.96)	1.73 (0.88)	1.86 (0.94)	1.81 (0.91)	1.81 (0.92)	1.86 (0.95)	1.84 (0.94)	1.98 (0.98)	2.02 (1.01)	2.05 (1.01)
Observed	1.90 (0.96)	1.73 (0.88)	1.86 (0.94)	1.80 (0.91)	1.81 (0.92)	1.85 (0.95)	1.83 (0.94)	1.97 (0.98)	2.01 (1.00)	2.04 (1.01)
**Mean sum score (M[SD])**										
Imputed	1.82 (0.71)	1.68 (0.63)	1.78 (0.69)	1.73 (0.66)	1.71 (0.66)	1.74 (0.68)	1.71 (0.67)	1.81 (0.71)	1.85 (0.73)	1.87 (0.74)
Observed	1.81 (0.71)	1.67 (0.63)	1.78 (0.68)	1.73 (0.66)	1.70 (0.65)	1.73 (0.68)	1.70 (0.66)	1.79 (0.70)	1.84 (0.72)	1.86 (0.73)

Note. This figure shows means (M) and standard deviations (SD) of each item, comparing imputed data and observed data among boys.

Supplementary Table 45. Sensitivity analyses: Comparing imputed item values with observed item values across time; **Girls.**

	2010	2011	2012	2013	2014	2015	2016	2017	2018	2019
**Item 1**										
Imputed	2.39 (0.99)	2.25 (0.96)	2.40 (0.99)	2.39 (0.99)	2.40 (1.00)	2.47 (1.02)	2.47 (1.02)	2.53 (1.01)	2.54 (1.02)	2.57 (1.01)
Observed	2.38 (0.99)	2.25 (0.96)	2.40 (0.98)	2.38 (0.98)	2.40 (1.00)	2.46 (1.02)	2.47 (1.02)	2.53 (1.01)	2.54 (1.02)	2.57 (1.01)
**Item 2**										
Imputed	2.08 (0.97)	2.02 (0.96)	2.16 (0.99)	2.11 (0.97)	2.11 (0.98)	2.13 (0.99)	2.13 (1.00)	2.19 (1.00)	2.22 (1.02)	2.27 (1.02)
Observed	2.07 (0.97)	2.02 (0.96)	2.15 (0.99)	2.11 (0.97)	2.11 (0.98)	2.12 (0.99)	2.13 (0.99)	2.18 (1.00)	2.22 (1.01)	2.27 (1.02)
**Item 3**										
Imputed	2.03 (0.98)	2.04 (0.97)	2.12 (1.00)	2.13 (1.01)	2.09 (1.02)	2.10 (1.02)	2.11 (1.03)	2.20 (1.03)	2.22 (1.04)	2.29 (1.03)
Observed	2.03 (0.97)	2.03 (0.97)	2.12 (0.99)	2.13 (1.01)	2.08 (1.02)	2.10 (1.02)	2.11 (1.03)	2.19 (1.03)	2.21 (1.03)	2.28 (1.03)
**Item 4**										
Imputed	1.89 (0.99)	1.84 (0.97)	1.96 (1.01)	1.97 (1.02)	1.99 (1.04)	2.05 (1.06)	2.03 (1.06)	2.10 (1.06)	2.15 (1.07)	2.18 (1.07)
Observed	1.89 (0.99)	1.83 (0.97)	1.96 (1.01)	1.97 (1.02)	1.99 (1.04)	2.04 (1.05)	2.03 (1.06)	2.10 (1.06)	2.14 (1.07)	2.18 (1.07)
**Item 5**										
Imputed	2.01 (0.97)	1.96 (0.95)	2.05 (0.98)	2.06 (0.98)	1.98 (0.97)	2.04 (0.99)	2.03 (1.00)	2.17 (1.02)	2.19 (1.03)	2.21 (1.03)
Observed	2.01 (0.97)	1.96 (0.95)	2.04 (0.98)	2.05 (0.98)	1.97 (0.97)	2.03 (0.99)	2.03 (1.00)	2.17 (1.01)	2.18 (1.03)	2.21 (1.03)
**Item 6**										
Imputed	2.41 (1.02)	2.31 (1.01)	2.43 (1.01)	2.46 (1.02)	2.49 (1.03)	2.55 (1.04)	2.57 (1.05)	2.71 (1.02)	2.74 (1.03)	2.77 (1.02)
Observed	2.40 (1.02)	2.31 (1.00)	2.43 (1.01)	2.45 (1.02)	2.49 (1.03)	2.55 (1.04)	2.57 (1.05)	2.71 (1.02)	2.74 (1.03)	2.77 (1.02)
**Mean sum score**										
Imputed	2.13 (0.77)	2.07 (0.77)	2.19 (0.79)	2.19 (0.79)	2.18 (0.79)	2.22 (0.81)	2.23 (0.82)	2.32 (0.81)	2.34 (0.82)	2.38 (0.81)
Observed	2.13 (0.77)	2.07 (0.76)	2.19 (0.78)	2.18 (0.79)	2.17 (0.79)	2.22 (0.81)	2.22 (0.82)	2.31 (0.81)	2.34 (0.82)	2.38 (0.81)

Note. This figure shows means (M) and standard deviations (SD) of each item, comparing imputed data and observed data among girls.

Supplementary Table 46. Sensitivity analyses: Measurement invariance across time by age groups using multiple imputed data: **Girls**

Model	χ^2(df)	p	CFI	RMSEA (90%CI)	TLI	SRMR	ΔCFI	ΔRMSEA
Age 13								
Baseline	1981.776 (90)	<.001	0.994	0.058 (0.056, 0.061)	0.990	0.024	-	-
Baseline*	1126.408 (80)	<.001	0.997	0.046 (0.044, 0.049)	0.994	0.018	-	-
Thresholds	1242.554 (134)	<.001	0.996	0.037 (0.035, 0.039)	0.996	0.018	< 0.001	-0.009
Thresholds and loadings	1086.23 (179)	<.001	0.997	0.029 (0.027, 0.030)	0.998	0.018	0.001	-0.008
Thresholds, loadings, intercepts	1816.144 (224)	<.001	0.995	0.034 (0.033, 0.035)	0.997	0.019	-0.002	0.005
Age 14								
Baseline	2434.364 (90)	<.001	0.993	0.066 (0.064, 0.068)	0.989	0.025		
Baseline*	1239.425 (80)	<.001	0.997	0.049 (0.047, 0.052)	0.994	0.019		
Thresholds	1336.501 (134)	<.001	0.996	0.039 (0.037, 0.041)	0.996	0.019	< 0.001	-0.011
Thresholds and loadings	1180.094 (179)	<.001	0.997	0.031 (0.029, 0.032)	0.998	0.019	< 0.001	-0.008
Thresholds, loadings, intercepts	1901.946 (224)	<.001	0.995	0.035 (0.034, 0.037)	0.997	0.019	-0.002	0.005
Age 15								
Baseline	2642.696 (90)	<.001	0.992	0.069 (0.066, 0.071)	0.986	0.027	-	-
Baseline*	1354.043 (80)	<.001	0.996	0.051 (0.049, 0.054)	0.992	0.020	-	-
Thresholds	1453.508 (134)	<.001	0.996	0.040 (0.039, 0.042)	0.995	0.020	< 0.001	-0.0110
Thresholds and loadings	1224.651 (179)	<.001	0.997	0.031 (0.030, 0.033)	0.997	0.020	< 0.001	-0.009
Thresholds, loadings, intercepts	2219.465 (224)	<.001	0.993	0.038 (0.037, 0.04)	0.996	0.020	-0.003	0.007
Partial Invariance (free intercepts item 6)	1633.721 (215)	<.001	0.995	0.033 (0.032, 0.035)	0.997	0.020	-0.001	0.002
Age 16								
Baseline	2668.61 (90)	<.001	0.988	0.073 (0.071, 0.076)	0.980	0.028	-	-
Baseline*	1407.049 (80)	<.001	0.994	0.056 (0.053, 0.059)	0.988	0.021	-	-
Thresholds	1485.462 (134)	<.001	0.994	0.044 (0.042, 0.046)	0.993	0.021	< 0.001	-0.012
Thresholds and loadings	1330.315 (179)	<.001	0.995	0.035 (0.033, 0.037)	0.995	0.022	< 0.001	-0.009
Thresholds, loadings, intercepts	1999.627 (224)	<.001	0.992	0.039 (0.037, 0.040)	0.994	0.022	-0.003	0.004
Partial Invariance (free intercepts item 6)	1627.024 (215)	<.001	0.993	0.035 (0.034, 0.037)	0.995	0.022	-0.001	< 0.001
Age 17-18					.			
Baseline	2899.119 (90)	<.001	0.985	0.073 (0.070, 0.075)	0.975	0.029	-	-
Baseline*	1739.977 (80)	<.001	0.991	0.059 (0.057, 0.062)	0.983	0.023	-	-
Thresholds	1804.076 (134)	<.001	0.991	0.046 (0.044, 0.048)	0.99	0.023	< 0.001	-0.013
Thresholds and loadings	1511.457 (179)	<.001	0.993	0.036 (0.034, 0.037)	0.994	0.023	0.001	-0.010
Thresholds, loadings, intercepts	2122.731 (224)	<.001	0.990	0.038 (0.036, 0.039)	0.993	0.023	-0.003	0.002
Partial Invariance (free intercepts item 6)	1884.649 (215)	<.001	0.991	0.036 (0.035, 0.038)	0.994	0.023	-0.002	< 0.001

Note. χ^2= chi-square goodness-of-fit based on the Satorra-Bentler correction; df = degrees of freedom; CFI; comparative fit index; RMSEA = root mean square error of approximation; CI = confidence interval. TLI = Tucker Lewis Index. SRMR = standardized root mean squared residual; ΔCFI/RMSEA = Change in CFI/RMSEA between models.

* Models accounting for one correlated error term between item 3 and 4.

Supplementary Table 47. Sensitivity analyses: Measurement invariance across time by age groups using multiple imputed data: **Boys**

Model	χ^2(df)	p	CFI	RMSEA (90%CI)	TLI	SRMR	ΔCFI	ΔRMSEA
Age 13								
Baseline	1590.780 (90)	<.001	0.990	0.052 (0.050, 0.054)	0.983	0.024	-	-
Baseline*	1058.490 (80)	<.001	0.994	0.045 (0.042, 0.047)	0.988	0.019	-	-
Thresholds	1141.272 (134)	<.001	0.993	0.035 (0.033, 0.037)	0.993	0.019	< 0.001	-0.010
Thresholds and loadings	1018.400 (179)	<.001	0.994	0.028 (0.026, 0.029)	0.995	0.019	0.001	-0.007
Thresholds, loadings, intercepts	1500.191 (224)	<.001	0.992	0.031 (0.029, 0.032)	0.994	0.020	-0.003	0.003
Partial Invariance (free intercepts item 5)	1308.312 (215)	<.001	0.993	0.029 (0.027, 0.030)	0.995	0.020	-0.002	0.001
Age 14								
Baseline	2086.146 (90)	<.001	0.987	0.061 (0.058, 0.063)	0.979	0.025	-	-
Baseline*	1282.797 (80)	<.001	0.992	0.050 (0.048, 0.052)	0.986	0.019	-	-
Thresholds	1307.282 (134)	<.001	0.993	0.038 (0.036, 0.04)	0.992	0.019	< 0.001	-0.012
Thresholds and loadings	1129.082 (179)	<.001	0.994	0.030 (0.028, 0.031)	0.995	0.019	0.001	-0.008
Thresholds, loadings, intercepts	1663.248 (224)	<.001	0.991	0.033 (0.031, 0.034)	0.994	0.020	-0.003	0.003
Partial Invariance	1482.874 (215)	<.001	0.992	0.031 (0.030, 0.033)	0.994	0.020	-0.002	0.002
Age 15								
Baseline	2281.326 (90)	<.001	0.987	0.063 (0.061, 0.065)	0.978	0.024	-	-
Baseline*	1278.470 (80)	<.001	0.993	0.050 (0.047, 0.052)	0.987	0.018	-	-
Thresholds	1334.972 (134)	<.001	0.993	0.038 (0.037, 0.040)	0.992	0.018	< - 0.001	-0.011
Thresholds and loadings	1163.515 (179)	<.001	0.994	0.030 (0.028, 0.032)	0.995	0.018	0.001	-0.008
Thresholds, loadings, intercepts	1876.546 (224)	<.001	0.990	0.035 (0.033, 0.036)	0.993	0.019	-0.004	0.0048
Partial Invariance (free intercepts item 5)	1521.590 (215)	<.001	0.992	0.032 (0.030, 0.033)	0.995	0.018	-0.002	0.0015
Age 16								
Baseline	2397.961 (90)	<.001	0.983	0.068 (0.066, 0.070)	0.972	0.025	-	-
Baseline*	1355.996 (80)	<.001	0.991	0.054 (0.051, 0.056)	0.982	0.019	-	-
Thresholds	1431.457 (134)	<.001	0.990	0.042 (0.040, 0.044)	0.989	0.019	< - 0.001	-0.012
Thresholds and loadings	1262.426 (179)	<.001	0.992	0.033 (0.031, 0.035)	0.993	0.019	0.002	-0.009
Thresholds, loadings, intercepts	1862.385 (224)	<.001	0.988	0.036 (0.035, 0.038)	0.992	0.020	-0.0041	0.003
Partial Invariance (free intercepts item 5)	1554.419 (215)	<.001	0.990	0.033 (0.032, 0.035)	0.993	0.020	-0.002	< 0.001
Age 17-18								
Baseline	1921.715 (90)	<.001	0.986	0.060 (0.058, 0.063)	0.976	0.023	-	-
Baseline*	1369.937 (80)	<.001	0.990	0.054 (0.051, 0.056)	0.981	0.019	-	-
Thresholds	1382.350 (134)	<.001	0.990	0.041 (0.039, 0.043)	0.989	0.019	< -0.001	-0.013
Thresholds and loadings	1188.661 (179)	<.001	0.992	0.032 (0.030, 0.034)	0.993	0.019	0.002	-0.009
Partial Invariance (free intercepts item 5)	1537.787 (224)	<.001	0.990	0.032 (0.031, 0.034)	0.993	0.019	-0.002	< 0.001

Note. χ^2= chi-square goodness-of-fit based on the Satorra-Bentler correction; df = degrees of freedom; CFI; comparative fit index; RMSEA = root mean square error of approximation; CI = confidence interval. TLI = Tucker Lewis Index. SRMR = standardized root mean squared residual; ΔCFI/RMSEA = Change in CFI/RMSEA between models.

* Models accounting for one correlated error term between item 3 and 4.

Supplementary Figure 8. Sensitivity analyses: Trends in latent mean scores based on multiple imputed data: **Girls**

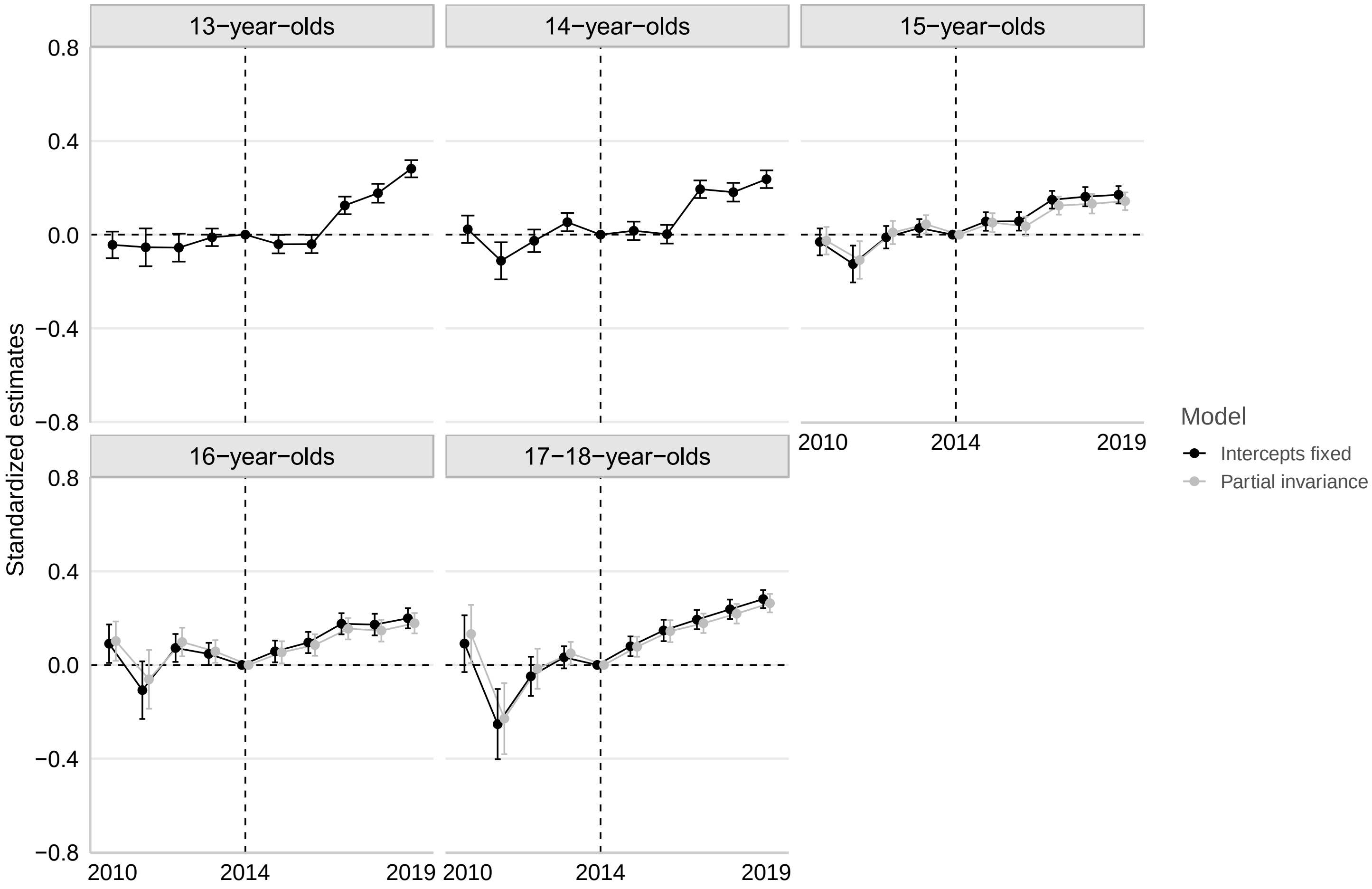

Note. This figure shows trends in latent mean scores (in standardized units) from the above described analysis based on 15 multiple imputed datasets. Intercepts fixed = Estimates based on scalar invariance model (fixing thresholds, loadings and intercepts). Partial invariance = Estimates based on partial scalar invariance models allowing intercepts for item 6 (worry) to freely estimated. Note that the partial invariance estimates are only shown for age groups where full scalar invariance was not achieved. The estimates from the two models have been slightly dodged horizontally to improve readability.

Supplementary Figure 9. Sensitivity analyses: Trends in latent mean scores based on multiple imputed data: **Boys**

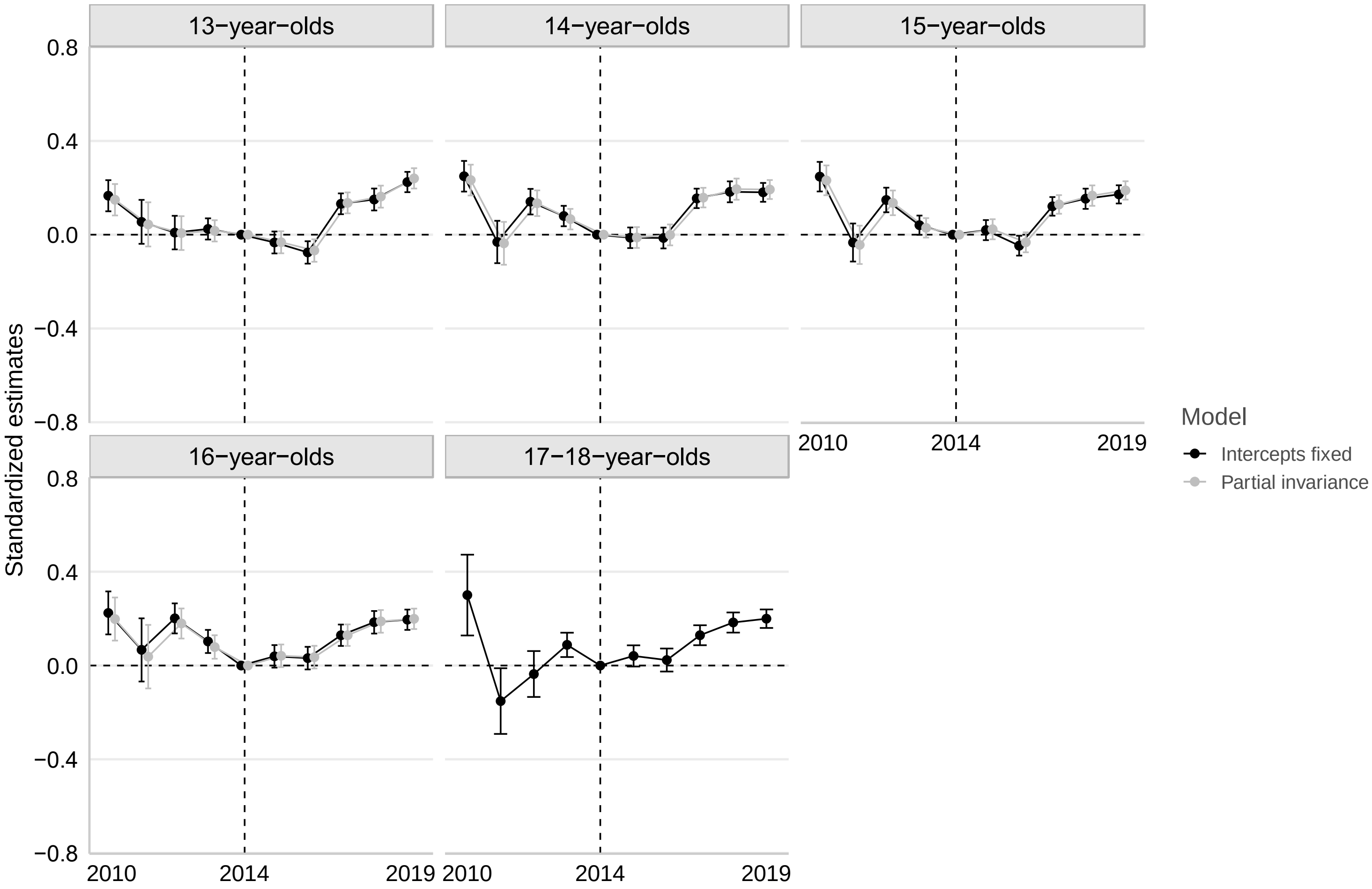

Note. This figure shows trends in latent mean scores (in standardized units) from the above described analysis based on 15 multiple imputed datasets. Intercepts fixed = Estimates based on scalar invariance model (fixing thresholds, loadings and intercepts). Partial invariance = Estimates based on partial scalar invariance models allowing intercepts for item 5 (stiff/tense) to freely estimated. Note that the partial invariance estimates are only shown for age groups where full scalar invariance was not achieved (based on a criteria of . The estimates from the two models have been slightly dodged horizontally to improve readability.

Supplementary Table 48. Latent means across survey years by age groups in **Girls**, from models based on multiple imputed data. Comparing models not accounting (Intercepts fixed) and accounting (partial invariance) for non-invariant parameters.

	Scalar invariance		Partial invariance*	
**Year**	β (95% CI)	p	β (95% CI)	p
**13-year-olds**				
2010	-0.044 (-0.101, 0.013)	0.134		
2011	-0.054 (-0.135, 0.027)	0.191		
2012	-0.055 (-0.115, 0.005)	0.070		
2013	-0.011 (-0.049, 0.026)	0.561		
2014	**ref.**			
2015	-0.041 (-0.08, -0.001)	0.043		
2016	-0.04 (-0.079, -0.002)	0.042		
2017	0.125 (0.087, 0.163)	<0.001		
2018	0.177 (0.137, 0.218)	<0.001		
2019	0.282 (0.245, 0.319)	<0.001		
**14-year-olds**				
2010	0.023 (-0.036, 0.082)	0.440		
2011	-0.112 (-0.191, -0.032)	0.006		
2012	-0.026 (-0.075, 0.022)	0.289		
2013	0.053 (0.015, 0.092)	0.007		
2014	**ref.**			
2015	0.017 (-0.022, 0.056)	0.403		
2016	0.002 (-0.037, 0.042)	0.913		
2017	0.194 (0.157, 0.232)	<0.001		
2018	0.181 (0.141, 0.222)	<0.001		
2019	0.237 (0.199, 0.275)	<0.001		
**15-year-olds**				
2010	-0.031 (-0.088, 0.027)	0.295	-0.026 (-0.084, 0.033)	0.390
2011	-0.125 (-0.204, -0.047)	0.002	-0.108 (-0.188, -0.028)	0.008
2012	-0.011 (-0.059, 0.037)	0.655	0.009 (-0.04, 0.059)	0.708
2013	0.028 (-0.01, 0.067)	0.145	0.044 (0.005, 0.084)	0.026
2014	**ref.**		**ref.**	
2015	0.056 (0.017, 0.096)	0.005	0.052 (0.011, 0.092)	0.012
2016	0.057 (0.017, 0.097)	0.005	0.036 (-0.005, 0.076)	0.082
2017	0.149 (0.111, 0.187)	<0.001	0.124 (0.086, 0.163)	<0.001
2018	0.162 (0.121, 0.203)	<0.001	0.132 (0.091, 0.174)	<0.001
2019	0.170 (0.133, 0.208)	<0.001	0.143 (0.105, 0.18)	<0.001
**16-year-olds**				
2010	0.091 (0.008, 0.173)	0.031	0.102 (0.018, 0.186)	0.017
2011	-0.108 (-0.231, 0.015)	0.086	-0.062 (-0.187, 0.064)	0.334
2012	0.072 (0.013, 0.132)	0.018	0.098 (0.037, 0.159)	0.002
2013	0.047 (0, 0.094)	0.052	0.057 (0.009, 0.106)	0.020
2014	**ref.**		**ref.**	
2015	0.058 (0.011, 0.104)	0.015	0.054 (0.006, 0.101)	0.026
2016	0.096 (0.05, 0.141)	<0.001	0.085 (0.039, 0.131)	<0.001
2017	0.176 (0.131, 0.221)	<0.001	0.154 (0.109, 0.2)	<0.001
2018	0.172 (0.126, 0.218)	<0.001	0.147 (0.1, 0.193)	<0.001
2019	0.199 (0.156, 0.242)	<0.001	0.178 (0.135, 0.222)	<0.001
**17-18 year-olds**				
2010	0.091 (-0.03, 0.212)	0.141	0.132 (0.008, 0.257)	0.037
2011	-0.253 (-0.403, -0.103)	<0.001	-0.229 (-0.381, -0.078)	0.003
2012	-0.049 (-0.132, 0.035)	0.256	-0.016 (-0.102, 0.069)	0.706
2013	0.032 (-0.015, 0.08)	0.180	0.05 (0.001, 0.098)	0.045
2014	**ref.**			
2015	0.080 (0.037, 0.122)	<0.001	0.078 (0.035, 0.121)	<0.001
2016	0.147 (0.102, 0.193)	<0.001	0.144 (0.098, 0.191)	<0.001

2017	0.193 (0.152, 0.234)	<0.001	0.178 (0.136, 0.219)	<0.001
2018	0.238 (0.196, 0.279)	<0.001	0.219 (0.177, 0.261)	<0.001
2019	0.281 (0.243, 0.32)	<0.001	0.264 (0.225, 0.303)	<0.001

Note. β = standardized regression coefficient. 95% CI =95 % confidence intervals of β. * Partial invariance models allowed intercepts of item 6 (worry) to be freely estimated. Note that for partial invariance, estimates are only available for models failing to achieve full scalar invariance based on model fit indices.

Supplementary Table 49. Latent means across survey years by age groups in **Boys**, from models based on multiple imputed data. Comparing models not accounting (Intercepts fixed) and accounting (partial invariance) for non-invariant parameters.

	**Scalar invariance**		**Partial invariance***	
**Year**	β (95% CI)	p	β (95% CI)	p
**13-year-olds**				
2010	0.166 (0.1, 0.233)	<0.001	0.149 (0.082, 0.216)	<0.001
2011	0.055 (-0.039, 0.149)	0.252	0.044 (-0.05, 0.139)	0.361
2012	0.009 (-0.062, 0.08)	0.805	0.007 (-0.065, 0.079)	0.848
2013	0.025 (-0.02, 0.07)	0.284	0.017 (-0.029, 0.062)	0.473
2014	**ref.**		**ref.**	
2015	-0.033 (-0.08, 0.014)	0.165	-0.032 (-0.079, 0.015)	0.179
2016	-0.076 (-0.124, -0.028)	0.002	-0.068 (-0.116, -0.019)	0.006
2017	0.132 (0.088, 0.176)	<0.001	0.136 (0.091, 0.18)	<0.001
2018	0.15 (0.104, 0.197)	<0.001	0.163 (0.116, 0.21)	<0.001
2019	0.225 (0.182, 0.268)	<0.001	0.24 (0.197, 0.284)	<0.001
**14-year-olds**				
2010	0.25 (0.184, 0.315)	<0.001	0.233 (0.167, 0.299)	<0.001
2011	-0.031 (-0.122, 0.06)	0.502	-0.037 (-0.128, 0.055)	0.431
2012	0.141 (0.086, 0.196)	<0.001	0.134 (0.079, 0.189)	<0.001
2013	0.08 (0.036, 0.123)	<0.001	0.067 (0.023, 0.11)	0.003
2014	**ref.**		**ref.**	
2015	-0.013 (-0.057, 0.031)	0.565	-0.012 (-0.056, 0.033)	0.601
2016	-0.014 (-0.058, 0.031)	0.541	-0.001 (-0.045, 0.044)	0.973
2017	0.155 (0.113, 0.197)	<0.001	0.158 (0.116, 0.2)	<0.001
2018	0.183 (0.138, 0.228)	<0.001	0.194 (0.149, 0.239)	<0.001
2019	0.181 (0.14, 0.221)	<0.001	0.193 (0.152, 0.233)	<0.001
**15-year-olds**				
2010	0.248 (0.185, 0.311)	<0.001	0.232 (0.168, 0.296)	<0.001
2011	-0.033 (-0.115, 0.048)	0.422	-0.043 (-0.125, 0.039)	0.302
2012	0.148 (0.096, 0.2)	<0.001	0.136 (0.083, 0.189)	<0.001
2013	0.041 (0, 0.082)	0.051	0.029 (-0.012, 0.071)	0.170
2014	**ref.**		**ref.**	
2015	0.02 (-0.023, 0.062)	0.367	0.023 (-0.02, 0.066)	0.288
2016	-0.047 (-0.089, -0.004)	0.030	-0.032 (-0.075, 0.01)	0.136
2017	0.121 (0.081, 0.161)	<0.001	0.129 (0.089, 0.169)	<0.001
2018	0.154 (0.111, 0.197)	<0.001	0.167 (0.123, 0.21)	<0.001
2019	0.172 (0.133, 0.211)	<0.001	0.189 (0.15, 0.228)	<0.001
**16-year-olds**				
2010	0.224 (0.133, 0.316)	<0.001	0.198 (0.106, 0.29)	<0.001

2011	0.067 (-0.068, 0.201)	0.332	0.038 (-0.098, 0.173)	0.586
2012	0.201 (0.137, 0.265)	<0.001	0.179 (0.115, 0.244)	<0.001
2013	0.103 (0.053, 0.152)	<0.001	0.079 (0.029, 0.129)	0.002
2014	**ref.**		**ref.**	
2015	0.039 (-0.009, 0.087)	0.111	0.041 (-0.007, 0.09)	0.093
2016	0.032 (-0.017, 0.08)	0.199	0.036 (-0.013, 0.084)	0.151
2017	0.129 (0.083, 0.175)	<0.001	0.129 (0.083, 0.175)	<0.001
2018	0.184 (0.136, 0.232)	<0.001	0.188 (0.14, 0.236)	<0.001
2019	0.195 (0.152, 0.239)	<0.001	0.199 (0.156, 0.243)	<0.001
**17-18 year-olds**				
2010	0.3 (0.128, 0.473)	<0.001		
2011	-0.152 (-0.292, -0.012)	0.034		
2012	-0.037 (-0.134, 0.061)	0.464		
2013	0.088 (0.036, 0.14)	<0.001		
2014	**ref.**			
2015	0.041 (-0.005, 0.086)	0.079		
2016	0.023 (-0.026, 0.072)	0.352		
2017	0.129 (0.086, 0.172)	<0.001		
2018	0.183 (0.141, 0.226)	<0.001		
2019	0.2 (0.16, 0.239)	<0.001		

Note. β = standardized regression coefficient. 95% CI =95 % confidence intervals of β. * Partial invariance models allowed intercepts of item 5 (stiff/tense) to be freely estimated. Note that for partial invariance, estimates are only available for models failing to achieve full scalar invariance based on model fit indices.

References

Jorgensen, T. D., Pornprasertmanit, S., Schoemann, A. M., & Rosseel, Y. (2020). *semTools: Useful tools for structural equation modeling*. https://CRAN.R-project.org/package=semTools

Rosseel, Y. (2012). lavaan: An R Package for Structural Equation Modeling. *Journal of Statistical Software, 48*(2), 1–36.

Rubin, D. B. (1987). *Multiple imputation for survey nonresponse*. Wiley.

van Buuren, S., & Groothuis-Oudshoorn, K. (2011). mice: Multivariate Imputation by Chained Equations in R. *Journal of Statistical Software, 45*(3), 1–67. https://doi.org/10.18637/jss.v045.i03
